# Older fathers' children have lower evolutionary fitness across four centuries and in four populations

**DOI:** 10.1098/rspb.2017.1562

**Published:** 2017-09-13

**Authors:** Ruben C. Arslan, Kai P. Willführ, Emma M. Frans, Karin J. H. Verweij, Paul-Christian Bürkner, Mikko Myrskylä, Eckart Voland, Catarina Almqvist, Brendan P. Zietsch, Lars Penke

**Affiliations:** 1 Biological Personality Psychology, Georg Elias Müller Institute of Psychology, University of Göttingen, 37073 Göttingen, Germany; 2 Leibniz ScienceCampus Primate Cognition, 37073 Göttingen, Germany; 3 Max Planck Institute for Demographic Research, 18057 Rostock, Germany; 4 Department of Psychiatry, University of Oxford, Warneford Hospital, Oxford OX3 7JX, UK; 5 Department of Medical Epidemiology and Biostatistics, Karolinska Institutet, 171 77 Stockholm, Sweden; 6 Department of Biological Psychology, VU University, 1081 BT Amsterdam, The Netherlands; 7 School of Psychology, University of Queensland, St. Lucia, Brisbane, Queensland 4072, Australia; 8 Department of Psychology, University of Münster, 48149 Münster, Germany; 9 Department of Social Policy, London School of Economics and Political Science, London WC2A 2AE, UK; 10 Population Research Unit, University of Helsinki, 00100 Helsinki, Finland; 11 Department of Biophilosophy, Justus Liebig University Gießen, 35390 Gießen, Germany; 12 Pediatric Allergy and Pulmonology Unit at Astrid Lindgren Children's Hospital, Karolinska University Hospital, Stockholm, Sweden; 13 Genetic Epidemiology, QIMR Berghofer Medical Research Institute, Brisbane, Queensland 4006, Australia

**Keywords:** paternal age, evolutionary fitness, mutation, genetic load, reproductive success

## Abstract

Higher paternal age at offspring conception increases de novo genetic mutations. Based on evolutionary genetic theory we predicted older fathers' children, all else equal, would be less likely to survive and reproduce, i.e. have lower fitness. In sibling control studies, we find support for negative paternal age effects on offspring survival and reproductive success across four large populations with an aggregate *N* > 1.4 million. Three populations were pre-industrial (1670–1850) Western populations and showed negative paternal age effects on infant survival and offspring reproductive success. In twentieth-century Sweden, we found minuscule paternal age effects on survival, but found negative effects on reproductive success. Effects survived tests for key competing explanations, including maternal age and parental loss, but effects varied widely over different plausible model specifications and some competing explanations such as diminishing paternal investment and epigenetic mutations could not be tested. We can use our findings to aid in predicting the effect increasingly older parents in today's society will have on their children's survival and reproductive success. To the extent that we succeeded in isolating a mutation-driven effect of paternal age, our results can be understood to show that de novo mutations reduce offspring fitness across populations and time periods.

## 1. Background

A child carries on average about 60 genetic de novo single nucleotide mutations (SNMs), which were not present in either of the biological parents' genomes [1,2]. Of those that are not functionally neutral, most reduce evolutionary fitness, as random changes to well-calibrated systems usually do [3,4]. Importantly, de novo mutations can be dominantly lethal or sterility-inducing early in life, unlike inherited deleterious variants. The older a father is, the more de novo mutations his child will tend to carry. This is dictated by the fundamental fact that cell replication engenders errors [5], and male spermatogonial, but not female oogonial, stem cells replicate frequently, beginning a regular schedule of one division per 16 days in puberty [6].

Kong *et al*. sequenced the genomes of parent–child triplets and quartets, so that they could pinpoint mutations and their parental origin [1]. They found that a child's number of de novo SNMs could be predicted very well (94% non-stochastic variance explained) by the father's age at the child's birth, henceforth *paternal age*. Mothers appear to transmit only a third to half as many SNMs per year as fathers [4,7]. Thus, paternal age appears to be the main predictor of varying offspring de novo mutation load, in part because of its causal role and to a lesser extent because of its correlation with maternal age. SNMs are the most common mutational event, but copy number variants also increase with paternal age; other structural variants tend to come from the father too [8]. Aneuploidies (aberrant chromosome counts) are a well-known exception: they occur more often when older mothers conceive [2]. Subsequent studies have confirmed the central role of paternal age for mutations [4,6].

In clinical research, paternal age has shown usefulness as a placeholder variable for de novo mutations: after initial epidemiological studies reported paternal age effects on autism [9], sibling comparison studies confirmed they were not due to inherited dispositions [10]. Then, exome-sequencing studies corroborated the paternal age effects by directly counting mutations that were not present in either parent's exome and found a higher mutational burden in autistic children than in unaffected siblings [11]. These findings elucidated disease aetiology both from an evolutionary and a clinical standpoint, by explaining how an early-onset disease linked to very low reproductive success could linger in the face of natural selection.

Given the links enumerated above, paternal age should, via increased mutations, decrease offspring fitness. By fitness, we mean each offspring's average contribution to the gene pool of successive generations. We can approximate this contribution through the offspring's number of descendants [12].

So far, most paternal age effect studies have focused on medical, psychological and behavioural traits, such as physical and psychiatric disease, or intelligence [10,13–16]. Though many of these traits plausibly affect evolutionary fitness now, it is not always clear how they affected fitness before the twentieth century. Moreover, there are scant records on such traits from this time, and they are not necessarily comparable to modern records. Births and deaths, or baptisms and burials, on the other hand, have been meticulously recorded in churches. Survival and reproductive success were and still are good measures of evolutionary fitness. Fitness is the most ‘downstream’ phenotype of all, in the sense that all non-neutral mutations affect it by definition [17].

Paternal age effects on mutations should in principle be universal across species, but non-human animal studies have thus far been restricted to birds [18,19] and have, with one exception [19], been studied under the broader topic of senescence, without attempts to separate mutational or epigenetic effects from behavioural effects of parental senescence on breeding capability. Studies on humans have examined isolated fitness components such as infant survival, longevity, marriage or reproduction in single populations in one place and at one time [20–23]. Some such studies have focused on longevity, which has an ambiguous relationship to evolutionary fitness owing to life-history trade-offs, such as trading off higher early-life reproduction for earlier mortality [24]. Some have examined maternal age or birth order, but ignored paternal age [25]. Some focused on environmental explanations, such as decreased parental investment [26], but these are not necessarily sufficient to explain paternal age effects. In wild house sparrows, the age of the biological parents had negative consequences even in a cross-fostering experiment [19]. Such experiments are not possible in humans, but we can statistically adjust for proxy measures of parental investment. In all, owing to variable methodology and sample sizes across studies, we cannot reliably compare findings to discover theoretically meaningful moderators.

### (a) The present study

Here we investigated paternal age effects on offspring fitness, focusing on the offspring's reproductive success, i.e. their number of children. To be able to compare all children of a father, we also included children who had no children themselves, even if they died young. Reproductive success is a good predictor of an individual's contribution to the next generation's gene pool [12]. In addition, we separately examined early survival, marriage success and reproductive success as successive episodes across the lifespan during which natural and sexual selection occur. Based on evolutionary genetic theory, we predicted that in aggregate we would find small, negative effects of paternal age on offspring fitness throughout the lifespan [27]. Some de novo mutations will have large negative effects early on, but many more will be (nearly) neutral. In aggregate, on the population level, this implies a small stochastically variable increase in deleterious effects with paternal age.

Because humans do not time their reproduction randomly, paternal age effects may be confounded by social and genetic factors [28–30] that are associated with both age of reproduction and offspring reproductive success. Because we aimed to isolate *mutation-driven* effects of paternal age as thoroughly as possible, we analysed the paternal age effect within full biological sibships and adjusted for a between-family effect. This effectively controls for many potential confounds. Full siblings share a parental gene pool, so that genetic load, which accumulated over generations, is distributed across them randomly. Siblings also usually share much of their early environment, and access to resources such as wealth and land. Because social convention may additionally link inheritance to birth order, we also adjusted for other social factors, such as birth order and parental loss. Additionally, we examined grandpaternal age effects where possible.

In doing so, we try to accomplish two goals: first, to isolate a potential biological, mutation-driven effect of paternal age on offspring fitness, and second, to compare different populations in different times and places, with high statistical power and comparable methodology.

## 2. Methods

### (a) Populations

To test our hypotheses before the turn of the twentieth century, we used genealogies drawn from church records in the Saint-Lawrence valley, Québec (Canada), the Krummhörn (Germany) and four historical Swedish regions. To compare these populations with twentieth-century Sweden, we used a population-based linkage study from Swedish national health registers. To ensure minimal censoring we drew subsets with adequately complete records.

We used computerized and linked registers of births (and baptisms), deaths (and burials) and marriages to reconstruct family pedigrees and life histories for individuals. We call the individuals whose father's age we compared with their siblings' ‘anchors’ wherever it aids comprehension. Further descriptive statistics can be found in table 1 and on the online supplementary website at https://rubenarslan.github.io/paternal_age_fitness/ [31].

**Table 1.** Descriptive statistics. RS, reproductive success; IS, infant survival. Numbers in parentheses are standard deviations. Years refer to the birth years of the anchors. For twentieth-century Sweden, fertility-related numbers are from 1947 to 1959 (first *N* given) and mortality numbers are from 1969 to 2000 (second *N* given).

	1720–1850 Krummhörn	1670–1750 Québec	1760–1850 Sweden	twentieth-century Sweden
population *N*	80 808	459 591	271 130	8 201 968
anchor *N*	14 034	79 895	56 947	1 419 282/3 428 225
anchors/families (RS models)	9447/2186	68 724/12 205	56 663/14 746	1 408 177/884 975
anchors/families (IS models)	9447/2186	61 493/11 940	56 010/14 708	363 744/200 000
paternal age	35.23 (7.56)	36.28 (8.48)	34.37 (7.69)	31.84 (7.05)
maternal age	31.53 (5.88)	29.58 (6.66)	31.54 (6.32)	28.34 (6.11)
female/male infant mortality	11.1/12.9%	19.0/23.2%	12.0/14.1%	0.5/0.7%
fertility (married women)	3.66 (2.89)	7.71 (4.57)	3.6 (3.17)	2.15 (1.11)
male age at first child	29.29 (5.36)	27.92 (5.29)	28.13 (5.18)	28.07 (5.6)
male age at last child	39.6 (7.5)	44.19 (8.59)	37.52 (8.29)	33.57 (6.14)

The first population are inhabitants of the Krummhörn in contemporary Germany [32]. They were quite isolated and had a stable population size. We focused on the 14 034 anchors born between 1720 and 1835. Married female anchors from this period had on average 3.7 children.

The second population are the French settlers of the Saint-Lawrence valley in contemporary Québec, Canada [33,34]. They were an isolated frontier population in a harsh climate but they also had access to abundant resources and unsettled land. We focused on the 79 895 anchors born between 1670 and 1740. Married female anchors from this period had on average 7.7 children. In this dataset, we had access to deep pedigrees, allowing us to compare not only siblings for paternal age, but also cousins for grandpaternal age in a within-extended-family design.

The third population are Swedes in the Sundsvall, Northern Inland (Karesuando to Undersåker, includes Sami people), Linköping and Skellefteå regions [35,36]. All individuals in Skellefteå and most individuals in Sundsvall were linked between church parishes. In the other regions, some individuals appeared in more than one parish. We focused on the 56 947 anchors born between 1737 and 1850. Married female anchors from this period had on average 3.6 children.

Our modern data are the whole population of Sweden. The Swedish Multi-Generation Register includes records of individuals born after 1932 and alive by 1962, as well as their parents. The dataset was linked to the Cause of Death register that includes death dates. Information about marriages was derived from the population register and the Longitudinal Integration Database for Health Insurance and Labour Market Studies [37]. Individuals who ever had the civil status of married, widowed or divorced were counted as *ever married*. Because of censoring in this dataset, we focused on the 1 419 282 anchors born between 1947 and 1959 for reproductive outcomes and the 3 428 225 anchors born between 1969 and 2000 for survival outcomes. Ever married female anchors from the earlier period had on average 2.2 children (never married: 1.1). Hormonal contraception was widely available to and used by anchors born between 1947 and 1959.

### (b) Statistical approach

We employed generalized mixed-effect regressions with a group-level effect per family to compare full biological siblings within families. We used the R package brms [38] to fit Bayesian regression models using the probabilistic programming language Stan [39], and adjusted for average paternal age within families to isolate the effect of paternal age differences between siblings. We adjusted for birth cohort in 5-year groupings (small groupings at the edge of the range were lumped) to account for secular changes in mortality and fertility, as well as residual censoring. We adjusted for parental deaths in the first 45 years of life to remove effects related to orphanhood and parental senescence (0–1, 2–5, 6–10, … , 45+, unknown). We adjusted for maternal age (up to 20, 21–34, 35+), which we *binned* to reduce multicollinearity with paternal age and to capture nonlinear effects. We also adjusted for number of siblings, number of older siblings (0–5, 5+), and being born last. We used weakly informative priors that are documented in detail in the online electronic supplementary material. The modelling assumptions reflected herein were tested for robustness, as documented below.

We analysed reproductive success for *all* offspring, including those who died in childhood or never married. We used a two-process hurdle-Poisson family with a log link. In such a model, zeroes in the outcome variable are modelled as arising from a different process, e.g. not clearing the *hurdle* of survival and marriage before attempting reproduction. In the twentieth-century Swedish data, we fitted a simpler Poisson model because child mortality was very low.

We separated effects into four successive episodes of natural and sexual selection. To separate the episodes, we adjusted for success in the preceding episode: *e1* survival of the first year; *e2* survival until age 15 conditional on *e1* survival of the first year; *e3* marriage conditional on *e2*; and *e4* number of children, conditional on *e3*. For *e4,* we included only ever-married anchors and adjusted for their number of spouses. In twentieth-century Sweden, we also examined *e5* divorce, conditional on *e3*, even though this is arguably not clearly an episode of selection. All models were fitted using a Bernoulli regression with a cauchit link to decrease the influence of extreme values [40], except *e4* which was fitted using a Poisson regression with a log link. In twentieth-century Sweden, we could not fit our survival models to the whole available dataset for computational reasons and hence used a randomly drawn subset (approx. 10% of the 3.4 million available).

We used approximate leave-one-out cross-validation [41] as implemented in brms to compare four models: *m1* with a linear effect of paternal age, without the group-level effect for family; *m2* without a paternal age effect, but with the group-level effect; *m3* like *m2* but with a linear paternal age effect; and *m4,* like *m3,* but additionally with a thin-plate spline smooth [42] on the paternal age effect to capture nonlinearity. Comparing *m1* and *m3* allows us to assess the usefulness of group-level effects; comparing *m2* and *m3*, we test whether the inclusion of paternal age improves the model fit; comparing *m3* and *m4,* we test the paternal age effect for nonlinearity.

After this, we ran several robustness checks to test the modelling assumptions in our main models, using *m3* as the baseline model. We carried out the following analyses: *r1* relaxed exclusion criteria (not in twentieth-century Sweden); *r2* had only birth cohort as a covariate; *r3* adjusted for birth order continuously; *r4* adjusted for number of dependent siblings (younger than 5, alive at anchor birth) instead of birth order; *r5* interacted birth order with number of siblings; *r6* did not adjust for birth order; *r7* adjusted only for parental loss in the first 5 years; *r8* adjusted for being the first- or last-born adult son; *r9* adjusted for a continuous nonlinear thin-plate spline smooth [42] for birth year instead of 5-year bins; *r10* added a group-level slope for paternal age; *r11* included separate group-level effects for each parent instead of one per marriage; *r12* added a moderation by anchor sex; *r13* adjusted for paternal age at first birth; *r14* compared a model with linear group fixed effects; *r15* added a moderator by region and group-level effects by church parish (not in twentieth-century Sweden); *r16* was restricted to the region Skellefteå (only in historical Sweden); *r17* tested whether hypothetical cases of Down's syndrome could explain the effects; *r18* reversed hurdle Poisson and Poisson distribution for the respective populations; *r19* assumed a normal distribution for the outcome; *r20* did not adjust for maternal age; *r21* adjusted for maternal age continuously; *r22* relaxed exclusion criteria and included 30 more years of birth cohorts, allowing for more potential censoring; *r23* used different weakly informative priors; *r24* used non-informative priors (comparable with maximum likelihood); *r25* controlled for migration status (not in twentieth-century Sweden); *r26* separated parental age contributions (only in twentieth-century Sweden). More detailed descriptions of all robustness analyses can be found in the electronic supplementary material §6.2, code and detailed results are on the online supplementary website [31].

For the twentieth-century Sweden data, we used a random subset of 80 000 families in the robustness analyses for computational reasons. We re-ran analyses with all data if the paternal age effect deviated strongly from the *m3* estimate.

We also ran two sensitivity analyses to test whether results could be explained by late-life mortality or reproductive timing of the anchors. To contextualize contemporary reproductive timing trends, we also compared reproductive timing across populations.

Effect sizes were calculated as the median effect estimate of a 10-year increase in paternal age with a 95% credibility interval.

## 3. Results

**Figure 1.**
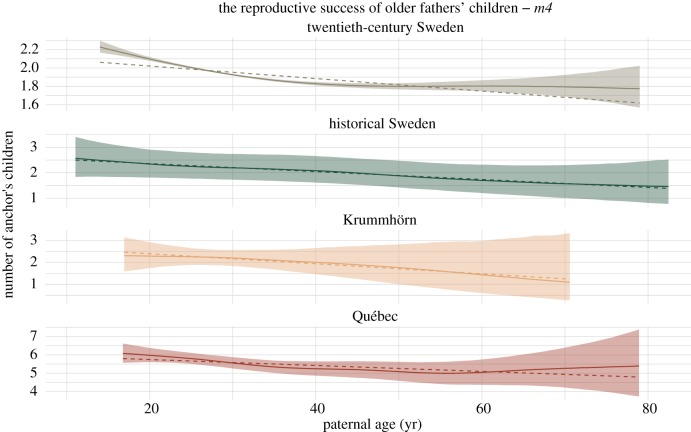
Paternal age effects on number of surviving children. Marginal effect plots for paternal age effect splines estimated in *m4*. Covariates were set to their mean or reference level, respectively. The solid lines show the posterior median; the dashed line is a linear line fit over the spline and inversely weighted by standard error to examine whether the spline fit deviates from linearity. The shaded areas show the 95% credibility intervals for the reference individuals and include uncertainty related to covariate effect sizes.

In our main model *m3*, we found negative effects of paternal age on anchor's number of children in all four populations: a decrease per decade of paternal age of −3.0% (95% credibility interval [−6.1, 0.2]) in Québec, −3.4% [−5.9, −0.9] in twentieth-century Sweden, −7.3% [−13.4, −1.1] in historical Sweden, and −8.4% [−24.8, 12.0] in the Krummhörn. These effects appeared to be fairly linear in *m4* (figure 1), although visual inspection and approximate leave-one-out cross-validation [41] showed the effect tapering off after age 45 in twentieth-century Sweden (approx. 4% of children were born to fathers older than 45, see electronic supplementary material, §5.4.5.1) and after age 50 in Québec in (approx. 8% of children, see electronic supplementary material, §3.4.5.1). In historical Sweden, paternal age had a slight positive effect in *m1* before using sibling comparisons, in the other populations the effect was negative in all models. In the Krummhörn population, the effects of birth order, maternal and paternal age could not be disentangled well, as credible intervals were very wide when these covariates were considered together. Credible intervals (95%) for paternal age excluded zero for *m3* in both Swedish populations and for *m4* in Québec and twentieth-century Sweden. These main models are detailed in the electronic supplementary material, §§2–5.

**Figure 2.**
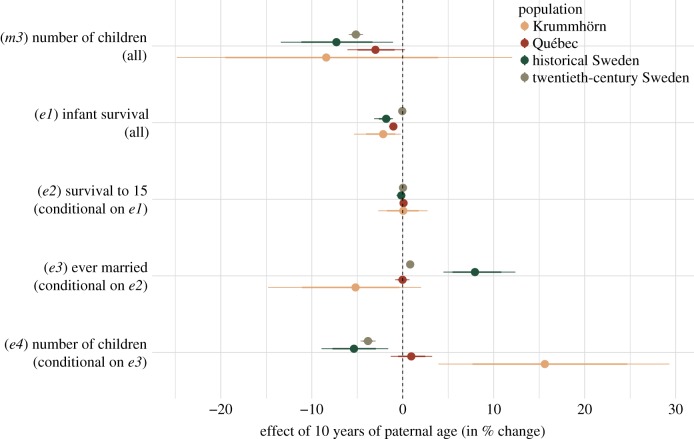
Paternal age effects on subsequent selective episodes. Estimated percentage changes in the respective selective episode (comparing children of 25- to 35-year-old fathers) with 80% and 95% credibility intervals.

In our selective episode analyses (figure 2), we consistently found small negative associations between paternal age and anchor's survival to the first year of life in the pre-industrial populations (*e1*). Comparing children of 25- and 35-year-old fathers yielded percentage decreases of −2.1 (95% credible interval [−0.2, −5.4]), −1.0 [−0.7, −1.5], and −1.8 [−1.1, −3.1] in the Krummhörn, Québec and historical Sweden respectively. In the twentieth-century Swedish population, infant mortality was very low, and the effect size of paternal age on infant survival, though negative, was correspondingly small (−0.05 [−0.03, −0.06]). Survival to age 15 years (*e2*) was not associated with paternal age (effects ranging from −0.2 to 0.1). Probability of ever marrying (*e3*) was inconsistently associated with paternal age, negatively in the Krummhörn population (−5.2), positively in historical Sweden (7.9), with negligible associations in Québec and modern Sweden (0.0 and 0.8), and the association in historical and twentieth-century Sweden turned negative when not accounting for parental loss (not shown). Number of children (*e4*), after accounting for marriage success, was negatively associated with paternal age in twentieth-century Sweden (−3.8 [−4.6; −3.0]) and historical Sweden (−5.4 [−8.9; −1.6]), but non-robustly positively associated in the Krummhörn population (15.62, negatively when not adjusting for birth order, not shown) and negligibly associated in Québec (0.9 [−1.3; 3.2]). Paternal age did not predict probability of divorce in twentieth-century Sweden (−0.3 [−0.78; 0.17]).

In the grandpaternal age analyses in Québec, we found negative effects of both the paternal and maternal grandfather's age, which were roughly equal in size (paternal grandfather: −7% [−4, −9%], maternal grandfather: −5% [−2, −8%] fewer children).

**Figure 3.**
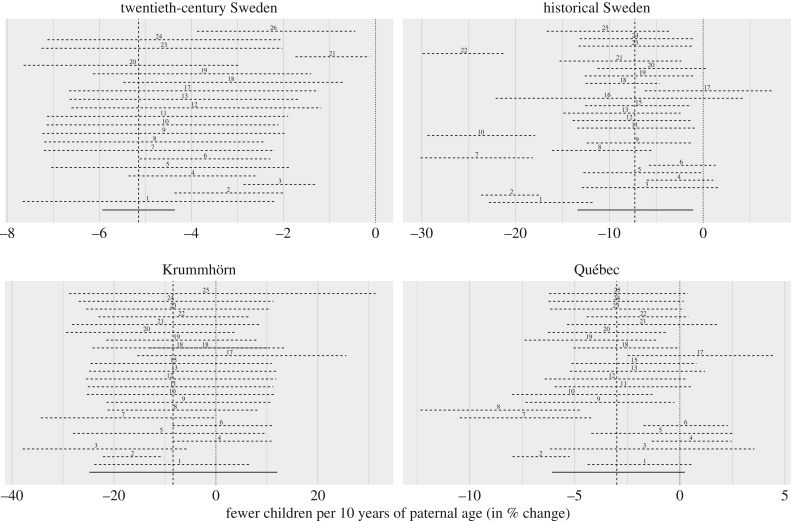
Robustness checks across 26 models. Estimates of the effect of a 10-year difference in paternal age on number of children from model *m3* and up to 26 variations on this basic model (described in the Methods section and in further detail on the electronic supplementary material website). The horizontal dashed and solid lines show 95% credibility intervals. The point and vertical dashed lines show the estimate from *m3*. The distance of the numbers to the vertical dashed line shows how much estimates can vary depending on the model specification. Estimates for the analyses in twentieth-century Sweden are based on a subset of the data for computational reasons (except models *m3*, *r3*, *r21*, and *r26*).

In our robustness analyses (figure 3), estimated paternal age effect sizes varied with our modelling assumptions. The paternal age effect was negative throughout almost all models in the two Swedish populations, and varied more widely in the Québec and Krummhörn models. In the Krummhörn, only the simplest model *r2* clearly supported a negative paternal age effect, but across robustness checks the estimate tended to be negative.

In our sensitivity analyses, we found mortality could mostly account for any paternal age effects on reproductive success in the two non-Swedish populations, but not in the Swedish populations. Among those who ever reproduced, paternal age did not predict reproductive success after accounting for anchor's age at first and last birth (confer supplement [31]).

Further details, including effect sizes and marginal effect plots for all covariates, model summaries, and R code for each of the models, can be found on the online supplementary website at https://rubenarslan.github.io/paternal_age_fitness/ [31].

## 4. Discussion

We found robust evidence for negative paternal age effects on reproductive success in all four populations. Results held up after adjusting for numerous covariates that capture alternative non-genetic explanations, including offspring sex, birth cohort, number of siblings, number of older siblings, maternal age, and loss of either parent up to age 45, and after checking robustness across 26 alternative models. In historical Sweden, a slight positive effect turned negative after we used sibling comparisons, showing that systematic confounding between reproductive timing and unobserved familial characteristics could obscure an effect. In all populations, effects were consistent with a roughly linear dose–response relationship between paternal age and number of children. Effects were largest in the Krummhörn (although estimates were uncertain in this smallest population), followed by historical Sweden, and similarly sized effects in Québec and twentieth-century Sweden. These differences seemed to be mainly driven by differences in the first selective episode, survival of the first year. The 95% credibility intervals for all effect sizes overlapped across populations.

Even across three generations, we found negative grandpaternal age effects on offspring reproductive success for both grandfathers in Québec.

When we separately examined the selective episodes along the lifespan, paternal age effects on survival to the first year were negative across all historical populations (−1% in Québec to −2% in the Krummhörn and historical Sweden), but negligibly small in twentieth-century Sweden (−0.05%). We found no robust pattern of effects on survival to age 15 and the odds of getting married. Some selective episode effects changed substantially depending on certain covariates, which may have resulted from adjusting for a collider, mediator, or highly collinear variable. Therefore, we advocate only cautious interpretation of the analyses where the estimate changed substantially upon removal of a covariate, especially in the Krummhörn. In the Swedish populations, the number of children was negatively associated with paternal age after adjusting for marriage success and survival to age 15. Consistent with this, our sensitivity analyses showed that mortality could not explain the paternal age effect in the Swedish populations. This may, however, reflect a mere difference in statistical power to detect remaining effects, as opposed to a substantive difference between populations.

In twentieth-century Sweden, the effect in the last selective episode, on number of children, was much stronger than the effect on infant mortality. Infant mortality in Sweden is among the lowest in the world. Because more than 99% of children brought to term in the years 1969 to 1999 survived, there was little room for selection during this selective episode. Future research should examine whether conditions that used to cause infant mortality, such as preterm birth, are simply no longer harmful thanks to advances in peri- and postnatal care, or whether selection has been partly displaced to before birth or to later in life. We might expect displaced selection to take place before birth in some cases, as abortions end one-fifth of all known pregnancies in Western Europe [43]. Most are elective, not therapeutic [44], but even women electing to have an abortion may do so selectively after considering their own age and paternal characteristics, including age [45]. Some paternal-age-linked conditions such as developmental disorders [4] might be detected in prenatal screening. Some diseases that would have led to early death in our historical populations might also put the afflicted at a disadvantage in later episodes of selection in twentieth-century Sweden, e.g. people with paternal-age-associated [4] developmental disorders might be less likely to marry and have children.

We tried to adjust for all non-biological explanations that could be modelled using our data. Still, it is possible that, for example, parental investment declines with paternal age in such a manner that our adjustments for parental loss, mother's age, birth order and various other covariates in our robustness analyses could only insufficiently correct for this. Such residual confounding might lead to inflated estimates of any biological paternal age effect.

Moreover, several non-genetic biological explanations for paternal age effects have been suggested in the literature. Eisenberg *et al*. [46] linked advanced paternal age to longer offspring telomeres, but it remains unclear whether this association is causal, whether it would differ between siblings and whether it could mediate phenotypic effects. Some authors [47,48] have also speculated that advanced paternal age might lead to errors in epigenetic regulation or might be linked to imprinting. Because preimplantation embryos undergo extensive demethylation and reprogramming [49,50], such transgenerational effects are controversial. Still, researchers [51–53] have searched for associations between paternal age and the methylation of certain genes in sperm and fetal cord blood. The use of small, clinical samples renders early work hard to generalize, but some associations have been reported.

Maternal age is another matter: its effects on aneuploidies are well established in the literature [54]. Although we adjusted for maternal age effects, parents' ages within families increase in lockstep. Their effects are thus difficult to separate in the largely pre-industrial monogamous populations. Even though maternal age is linked to aneuploidies, most aneuploid conceptions are not carried to term and even live-born children rarely get old. Only children with Down's syndrome live longer, but they are rarely fertile. Our robustness checks suggest Down's syndrome cannot fully explain the reported effects. In modern epidemiological data, specific syndromes could be easily excluded to test their contribution. Recent studies also estimated small effects of maternal age on single nucleotide de novo mutations [4,7]. Better understanding the mechanisms by which parental age is linked to offspring outcomes therefore seems to be a more worthwhile and achievable goal than perfectly separating each parent's contribution. Still, in modern Sweden we could separate parents' ages better, and in our robustness analyses paternal age still negatively predicted number of children after accounting for maternal age continuously, the average parental age for each parent and a dummy variable for teenage mothers.

Apart from these substantive alternative explanations, we also considered several methodological concerns. First and foremost, the highly collinear covariates maternal age, birth order and parental loss made it difficult to separate their contributions from that of paternal age. Standard errors were wide and different defensible operationalizations resulted in non-negligible effect size changes in our robustness analyses. Previous work rarely adjusted for parental loss to the extent that we did. This adjustment is debatable, because parental death can be both a cause and a consequence of offspring death. Still, from our robustness checks, we concluded that adjusting for parental loss is usually sensible and results of such adjustments should be reported in future work. Birth order, on the other hand, had little effect in most of our models, but adjusting for it often led to an increase in the paternal age effect size. Second, our church record data in particular have some shortcomings. Some children who died before baptism may have gone unrecorded, death records may be missing and migration might lead to unobserved censoring [55]. Fortunately, judging from the consistency of our robustness analyses, it is at least plausible that these problems are unrelated to paternal age after adjusting for covariates in our models, and we assume that by using four different populations we limited bias.

After all these adjustments, we still found negative paternal age effects on several measures of evolutionary fitness across populations. But what can explain these effects? The work of Kong *et al*. and others [1,6] has demonstrated a strong and likely causal effect of paternal age on de novo genetic mutations, but it is not clear that the paternal age effects reported here and in the literature are driven predominantly by de novo mutations [56]. One approach is to adjust for confounders, as we discuss above. Another is to derive expected effect size estimates from evolutionary genetic calculations. Gratten *et al*. [56] made the point that many reported paternal age effects in the psychiatric literature are implausibly large and calculated plausible effect sizes for mutational components of paternal age effects. Hayward *et al*. [22] estimated a paternal age effect on fitness components and attempted to compare their effect size with published estimates of the genome-wide deleterious mutation rate per generation (*U*) [3] times the mean selection effect against a deleterious mutation (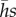), yielding the estimated mutation-caused decrease in fitness as a percentage [27]. As paternal age does not perfectly predict the number of de novo mutations per generation, any estimate of paternal age effects on fitness would be expected to be slightly lower than 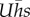. Unfortunately, no mean selection effect has been estimated for non-coding mutations yet and many unknowns and approximately-knowns enter the equation for estimates of the genome-wide deleterious mutation rate. Thus, only a range of plausible values can be drawn from the literature. Hayward *et al*. estimated values for 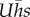 based only on non-synonymous mutations ranging from 0.016 to 0.031 [22,27,57]. Estimates including mutations at all functional sites are even less certain; 0.11–0.22 are high estimates based on assuming the same mean selection as against deleterious non-synonymous mutations. If we now assume an increase of two mutations per year of paternal age [1] and estimate the per-generation decline in fitness from de novo mutations by comparing the child of an average father aged 30 years, transmitting 60 mutations, with the child of a hypothetical father transmitting no mutations, for our models *m3* in all four populations, we obtain 0.16, 0.07, 0.20, and 0.14 in the Krummhörn, Québec, historical and twentieth-century Sweden respectively. Using the arguably better estimate from our robustness analysis *r26*, in which we could better adjust for maternal age in twentieth-century Sweden, we obtain an estimate of 0.065. Given the imperfect correlation between paternal age and de novo count, the variability of estimates in our robustness checks, sampling error and the plausibility of residual confounding, we think our estimates are on the high side of the real value, but not completely at odds with Hayward *et al*.'s calculations of 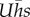 and consistent with their own estimated value of 0.12. We have also explored the relevant parameter space from Gratten *et al*. [56] and found the resulting effect sizes broadly consistent with the results from our infant survival models. These plausibility checks are documented in greater detail in the online supplement [31].

### (a) Implications and conclusions

Across four large population-based datasets, we found robust support for the prediction that higher paternal age linearly decreases offspring fitness. Although we cannot be sure that we succeeded in isolating an effect of de novo mutations given the multiple alternative explanations and methodological caveats, the effects are detectable in all four populations and hence plausibly caused to some extent by paternal age. Depending on their cause, but not only if that cause is mutational, paternal age effects could have implications for policy: descriptive data show a fall from 1930 to 1970 and a steady rise in maternal and paternal ages since 1970 in Sweden. However, average parental ages in 2010 were still lower than in 1737–1880 (electronic supplementary material, §7). Although people start reproducing later, they also stop earlier. Contrary to common news and lay scientific accounts, contemporary parents do not reproduce unprecedentedly late *on average* [1,45,58]. While advanced parental ages at *first* birth may entail smaller families, pre-industrial populations had similar average ages at birth and were not overwhelmed by mutational stress. So, we do not predict that contemporary reproductive timing will lead to unprecedented or unbearable de novo mutational loads and concomitant changes in the prevalence of genetic disorders. The decline in fitness with paternal age suggests that purifying selection is still effective in a modern population with hormonal contraception, social transfers and modern medicine. This runs counter to oft-repeated predictions of mutational doom by relaxed selection [3,59–61].

Although our design is not ideal for separating the influence of maternal and paternal age, many secular trends and policies will affect both. Future research could use genome-sequenced families with functionally annotated and phased mutations to better characterize the contribution of paternal age [4]. Future research could also isolate a biological paternal age effect on early mortality in non-human animals with large recorded pedigrees, such as artificially inseminated breeding cattle. This would rule out most social confounds by design, but the much shorter breeding lifespan might limit generalizability to humans.

## Supplementary Material

Model documentation, reproductive timing

## Data accessibility

Because of identification concerns and licensing restrictions, the data are only available from the dataset maintainers and underlie some usage restrictions.
