# Supplementary material for: Older fathers' children have lower evolutionary fitness across four centuries and in four populations
Source: Proc Biol Sci. 2017 Sep 13;284(1862):20171562. doi: 10.1098/rspb.2017.1562 (PMC5597845; doi:10.1098/rspb.2017.1562)

Paternal age and offspring fitness

Contents

1 Model description **2**
- 1.1 Estimation . . . 2
- 1.2 Covariates . . . 2
- 1.3 Model stratification . . . 2
- 1.4 Priors . . . 2
- 1.5 Robustness tests . . . 2

2 Krummhörn **3**
- 2.1 *m1*: No sibling comparison . . . 3
- 2.2 *m2*: Sibling comparison, no paternal age effect . . . 6
- 2.3 *m3*: Sibling comparison, linear paternal age effect . . . 7
- 2.4 *m4*: Sibling comparison, nonlinear paternal age effect . . . 11
- 2.5 Main model comparison . . . 14
- 2.6 *e1*: Selective episode: offspring survival of the first year . . . 15

3 Québec **18**
- 3.1 *m1*: No sibling comparison . . . 18
- 3.2 *m2*: Sibling comparison, no paternal age effect . . . 21
- 3.3 *m3*: Sibling comparison, linear paternal age effect . . . 22
- 3.4 *m4*: Sibling comparison, nonlinear paternal age effect . . . 26
- 3.5 Main model comparison . . . 29
- 3.6 *e1*: Selective episode: offspring survival of the first year . . . 30

4 Historical Sweden **33**
- 4.1 *m1*: No sibling comparison . . . 33
- 4.2 *m2*: Sibling comparison, no paternal age effect . . . 36
- 4.3 *m3*: Sibling comparison, linear paternal age effect . . . 37
- 4.4 *m4*: Sibling comparison, nonlinear paternal age effect . . . 41
- 4.5 Main model comparison . . . 44
- 4.6 *e1*: Selective episode: offspring survival of the first year . . . 45

5 20th-century Sweden **48**
- 5.1 *m1*: No sibling comparison . . . 48
- 5.2 *m2*: Sibling comparison, no paternal age effect . . . 51
- 5.3 *m3*: Sibling comparison, linear paternal age effect . . . 52
- 5.4 *m4*: Sibling comparison, nonlinear paternal age effect . . . 55
- 5.5 Main model comparison . . . 58
- 5.6 *e1*: Selective episode: offspring survival of the first year . . . 59

6 Robustness analyses documentation **62**
- 6.1 Table . . . 62
- 6.2 Model descriptions . . . 64

7 Reproductive timing in Sweden **69**

Title: Older fathers' children have lower evolutionary fitness across four centuries and in four populations
Authors: Arslan, R. C., Willführ, K. P., Frans, E., Verweij, K. J. H., Bürkner, P.-C., Myrskylä, M., Voland, E., Almqvist, C., Zietsch, B. P., Penke, L. Proceedings of the Royal Society B. Biological Sciences.

See also full supplementary website: https://rubenarslan.github.io/paternal_age_fitness/.

1 Model description

All of the models described below have the following in common. Only the robustness check models deviate from this in the way described in section 6.

1.1 Estimation

We fit all models using `brms` v. 1.2.0, a Bayesian regression analysis statistical package. `brms` uses `Stan`, a probabilistic programming langugage to fit models using Hamiltonian Monte Carlo.

1.2 Covariates

We adjusted for average paternal age within families to isolate the effect of paternal age differences between siblings. We further adjusted for birth cohort in five-year groupings (small groupings at the edge of the range were lumped) to account for secular changes in mortality and fertility, as well as residual censoring. We adjusted for parental deaths in the first 45 years of life to remove effects related to orphanhood and parental senescence (in categories of 0-1, 2-5, 6-10, . . . , 45+, unknown) for both parents separately. Parental loss at 45+ served as the reference category. We adjusted for maternal age (up to 20, 21-34, 35+), which we binned to reduce multicollinearity with paternal age and to capture nonlinear effects. A maternal age of 21-34 served as the reference category. We also adjusted for number of siblings continuously, number of older siblings (0-5, 5+), and being born last. Being first-born served as the reference category.

1.3 Model stratification

Except in model *m1*, we added group-level effects for each family (father-mother dyad) and then controlled for the average paternal age in the family. Hence, the effect of paternal age within families can be isolated from the effect between families. We are interested in the effect of paternal age within families, as this effect cannot be explained by e.g. genetic propensities of the father to reproduce later.

1.4 Priors

We used weakly informative normal priors with a standard deviation of 5 on the regression coefficients, Student's t priors with 3 degrees of freedom and a scale of 5 for the group-level standard deviations, and Student's t priors with 3 degrees of freedom and a scale of 10 for the splines.

1.5 Robustness tests

The modelling assumptions, including covariate choices and prior choices, reflected in the modelling approach above were tested for robustness, as documented in section 6 below.

2 Krummhörn

2.1 *m1*: No sibling comparison

Here, we ignore the pedigree structure of the data to see whether it matters for the estimation of the paternal age effect.

2.1.1 Model summary

Data: 9447 individuals.

Formula (Wilkinson notation): children ~ paternalage + birth_cohort + male + maternalage.factor + paternal_loss + maternal_loss + older_siblings + nr.siblings + last_born.

- **family**: hurdle_poisson
- **link**: log

2.1.2 Priors

prior	class
normal(0,5)	b

2.1.3 Population-level effects

Effect	Hurdle Odds ratio	Zero-truncated Poisson Hazard ratio
birth cohort 1760-1765	1.00 [0.90;1.12]	0.97 [0.72;1.33]
birth cohort 1765-1770	0.88 [0.80;0.97]	0.76 [0.58;0.99]
birth cohort 1770-1775	0.90 [0.82;0.99]	0.94 [0.73;1.23]
birth cohort 1775-1780	0.98 [0.89;1.07]	0.83 [0.64;1.07]
birth cohort 1780-1785	0.88 [0.80;0.97]	0.78 [0.60;1.00]
birth cohort 1785-1790	0.91 [0.83;1.00]	0.68 [0.53;0.88]
birth cohort 1790-1795	0.94 [0.86;1.02]	0.75 [0.60;0.96]
birth cohort 1795-1800	0.90 [0.83;0.97]	0.66 [0.52;0.83]
birth cohort 1800-1805	0.89 [0.82;0.96]	0.60 [0.49;0.75]
birth cohort 1805-1810	0.87 [0.80;0.95]	0.77 [0.62;0.97]
birth cohort 1810-1815	0.91 [0.84;0.98]	0.66 [0.54;0.82]
birth cohort 1815-1820	0.86 [0.80;0.93]	0.52 [0.42;0.64]
birth cohort 1820-1825	0.83 [0.77;0.90]	0.61 [0.50;0.76]
birth cohort 1825-1830	0.82 [0.76;0.89]	0.61 [0.50;0.75]
birth cohort 1830-1835	0.84 [0.78;0.91]	0.60 [0.49;0.74]
Intercept	5.34 [4.76;6.03]	0.69 [0.50;0.96]
last born	0.95 [0.91;0.99]	1.08 [0.96;1.22]
male	1.08 [1.05;1.12]	1.30 [1.19;1.41]
maternal loss 0-1	1.11 [0.97;1.26]	4.51 [3.18;6.55]
maternal loss 1-5	1.00 [0.92;1.08]	1.77 [1.44;2.19]
maternal loss 10-15	1.04 [0.97;1.11]	1.59 [1.32;1.94]
maternal loss 15-20	1.00 [0.94;1.08]	1.34 [1.10;1.63]
maternal loss 20-25	1.01 [0.95;1.08]	1.30 [1.09;1.56]
maternal loss 25-30	0.99 [0.94;1.05]	1.20 [1.02;1.41]
maternal loss 30-35	0.96 [0.91;1.02]	1.22 [1.06;1.43]

Effect	Hurdle Odds ratio	Zero-truncated Poisson Hazard ratio
maternal loss 35-40	0.98 [0.92;1.03]	1.07 [0.93;1.23]
maternal loss 40-45	0.97 [0.92;1.03]	1.29 [1.10;1.52]
maternal loss 5-10	1.07 [1.00;1.14]	1.59 [1.31;1.93]
maternalage factor 14-20	0.93 [0.78;1.09]	1.30 [0.83;2.04]
maternalage factor 35-50	1.01 [0.96;1.06]	1.16 [1.02;1.31]
nr siblings	1.00 [0.99;1.01]	1.09 [1.06;1.11]
older siblings 1	1.03 [0.99;1.08]	1.03 [0.91;1.16]
older siblings 2	0.98 [0.93;1.03]	0.93 [0.80;1.07]
older siblings 3	0.97 [0.92;1.03]	0.93 [0.79;1.10]
older siblings 4	0.96 [0.90;1.03]	0.94 [0.78;1.13]
older siblings 5+	0.99 [0.92;1.06]	0.72 [0.59;0.89]
paternal loss 0-1	0.86 [0.76;0.98]	1.76 [1.28;2.43]
paternal loss 1-5	0.97 [0.89;1.06]	1.67 [1.33;2.11]
paternal loss 10-15	1.00 [0.94;1.07]	1.16 [0.96;1.38]
paternal loss 15-20	0.90 [0.85;0.96]	1.09 [0.92;1.31]
paternal loss 20-25	0.88 [0.82;0.94]	1.15 [0.96;1.36]
paternal loss 25-30	0.99 [0.93;1.05]	1.05 [0.89;1.25]
paternal loss 30-35	0.96 [0.90;1.01]	0.98 [0.84;1.16]
paternal loss 35-40	0.97 [0.92;1.03]	0.98 [0.84;1.15]
paternal loss 40-45	0.99 [0.92;1.05]	1.15 [0.95;1.38]
paternal loss 5-10	0.93 [0.87;1.00]	1.21 [1.00;1.49]
paternalage	1.00 [0.97;1.03]	1.09 [1.01;1.17]

2.1.4 Paternal age effect

This is the effect of 10 years of paternal age within families on number of children, combined over the hurdle and Zero-truncated Poisson component, expressed as a change in percentage `((predicted value at t + 10y)/(predicted value at t)) - 1`.

effect	median_estimate	ci_95	ci_80
percentage change	-4.87	[-9.82; 0.17]	[-8.12;-1.57]

2.1.4.1 Marginal effect plot

Paternal age effect on number of children The shaded areas show the 95% and 80% credibility intervals for the reference individuals and include uncertainty related to covariate effect sizes.

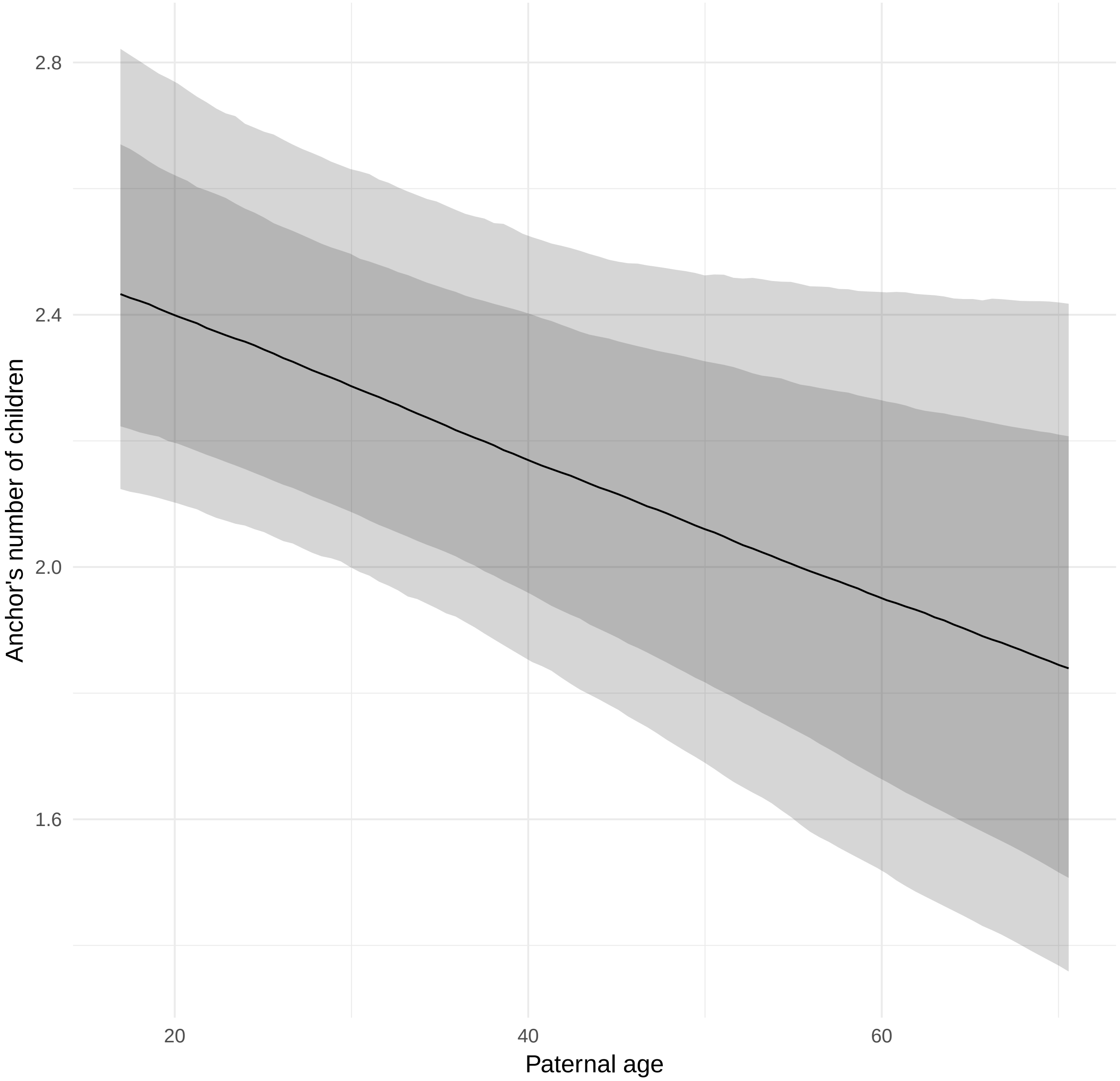

2.2 *m2*: Sibling comparison, no paternal age effect

Here, we compared siblings by including a random intercept for the family, but we modelled no effect for paternal age differences among siblings.

2.2.1 Model summary

Data: 9447 individuals nested in 2186 mother-father dyads.

Formula (Wilkinson notation): children ~ birth_cohort + male + maternalage.factor + paternalage.mean + paternal_loss + maternal_loss + older_siblings + nr.siblings + last_born + (1 | idParents).

- **family**: hurdle_poisson
- **link**: log

2.2.2 Priors

prior	class
normal(0,5)	b
student_t(3, 0, 5)	sd

2.2.3 Group-level effects

Component	Effect	Hurdle Estimate	Zero-truncated Poisson Estimate
idParents	sd(Intercept)	0.48 [0.39;0.56]	0.23 [0.20;0.25]

2.2.4 Population-level effects

Effect	Hurdle Odds ratio	Zero-truncated Poisson Hazard ratio
birth cohort 1760-1765	1.00 [0.89;1.13]	0.95 [0.68;1.32]
birth cohort 1765-1770	0.89 [0.80;1.00]	0.73 [0.55;0.97]
birth cohort 1770-1775	0.90 [0.80;1.00]	0.94 [0.71;1.25]
birth cohort 1775-1780	0.98 [0.89;1.09]	0.82 [0.61;1.08]
birth cohort 1780-1785	0.90 [0.81;1.01]	0.76 [0.57;1.02]
birth cohort 1785-1790	0.91 [0.82;1.01]	0.65 [0.49;0.86]
birth cohort 1790-1795	0.93 [0.84;1.03]	0.73 [0.56;0.96]
birth cohort 1795-1800	0.90 [0.82;1.00]	0.63 [0.49;0.80]
birth cohort 1800-1805	0.90 [0.82;0.99]	0.58 [0.45;0.74]
birth cohort 1805-1810	0.88 [0.80;0.97]	0.76 [0.60;0.97]
birth cohort 1810-1815	0.91 [0.83;1.00]	0.65 [0.51;0.82]
birth cohort 1815-1820	0.87 [0.80;0.95]	0.50 [0.39;0.62]
birth cohort 1820-1825	0.84 [0.77;0.91]	0.59 [0.47;0.74]
birth cohort 1825-1830	0.82 [0.75;0.89]	0.58 [0.46;0.72]
birth cohort 1830-1835	0.84 [0.76;0.92]	0.58 [0.46;0.73]
Intercept	5.15 [4.41;6.00]	0.69 [0.47;1.03]
last born	0.96 [0.91;1.00]	1.08 [0.96;1.23]
male	1.08 [1.04;1.12]	1.31 [1.20;1.43]
maternal loss 0-1	1.12 [0.96;1.30]	4.98 [3.49;7.17]
maternal loss 1-5	0.99 [0.90;1.08]	1.84 [1.46;2.33]

Effect	Hurdle Odds ratio	Zero-truncated Poisson Hazard ratio
maternal loss 10-15	1.03 [0.95;1.12]	1.62 [1.30;2.01]
maternal loss 15-20	1.01 [0.93;1.09]	1.38 [1.12;1.70]
maternal loss 20-25	1.01 [0.94;1.09]	1.31 [1.08;1.60]
maternal loss 25-30	0.98 [0.92;1.05]	1.22 [1.03;1.47]
maternal loss 30-35	0.95 [0.89;1.01]	1.25 [1.05;1.48]
maternal loss 35-40	0.97 [0.92;1.02]	1.08 [0.92;1.26]
maternal loss 40-45	0.97 [0.91;1.04]	1.32 [1.11;1.57]
maternal loss 5-10	1.08 [1.00;1.17]	1.64 [1.34;2.01]
maternalage factor 14-20	0.94 [0.79;1.13]	1.25 [0.81;1.98]
maternalage factor 35-50	1.01 [0.96;1.06]	1.20 [1.05;1.36]
nr siblings	1.00 [0.99;1.01]	1.08 [1.05;1.11]
older siblings 1	1.04 [0.99;1.09]	1.05 [0.92;1.20]
older siblings 2	0.98 [0.93;1.04]	0.96 [0.84;1.11]
older siblings 3	0.97 [0.91;1.03]	0.98 [0.83;1.15]
older siblings 4	0.97 [0.90;1.04]	1.00 [0.83;1.21]
older siblings 5+	0.99 [0.91;1.07]	0.78 [0.63;0.96]
paternal loss 0-1	0.86 [0.75;1.00]	1.84 [1.32;2.60]
paternal loss 1-5	0.97 [0.88;1.07]	1.75 [1.36;2.25]
paternal loss 10-15	1.01 [0.94;1.09]	1.19 [0.96;1.47]
paternal loss 15-20	0.91 [0.85;0.98]	1.12 [0.92;1.38]
paternal loss 20-25	0.89 [0.83;0.96]	1.18 [0.97;1.43]
paternal loss 25-30	0.99 [0.93;1.06]	1.07 [0.89;1.28]
paternal loss 30-35	0.97 [0.91;1.04]	0.99 [0.82;1.19]
paternal loss 35-40	0.99 [0.93;1.06]	0.99 [0.83;1.18]
paternal loss 40-45	0.99 [0.93;1.06]	1.16 [0.96;1.41]
paternal loss 5-10	0.94 [0.86;1.02]	1.24 [1.00;1.55]
paternalage mean	1.00 [0.96;1.03]	1.08 [0.99;1.18]

2.2.5 Paternal age effect

This is the effect of 10 years of paternal age within families on number of children, combined over the hurdle and Zero-truncated Poisson component, expressed as a change in percentage `((predicted value at t + 10y)/(predicted value at t)) - 1`.

This model did not contain a within family paternal age predictor.

2.3 *m3*: Sibling comparison, linear paternal age effect

Here, we compared siblings by including a random intercept for the family, and we modelled a linear effect for paternal age differences among siblings.

2.3.1 Model summary

Data: 9447 individuals nested in 2186 mother-father dyads.

Formula (Wilkinson notation): children ~ paternalage + birth_cohort + male + maternalage.factor + paternalage.mean + paternal_loss + maternal_loss + older_siblings + nr.siblings + last_born + (1 | idParents).

- **family**: hurdle_poisson
- **link**: log

2.3.2 Priors

prior	class
normal(0,5)	b
student_t(3, 0, 5)	sd

2.3.3 Group-level effects

Component	Effect	Hurdle Estimate	Zero-truncated Poisson Estimate
idParents	sd(Intercept)	0.47 [0.39;0.56]	0.22 [0.20;0.25]

2.3.4 Population-level effects

Effect	Hurdle Odds ratio	Zero-truncated Poisson Hazard ratio
birth cohort 1760-1765	1.00 [0.88;1.13]	0.95 [0.68;1.34]
birth cohort 1765-1770	0.89 [0.79;0.99]	0.73 [0.55;0.99]
birth cohort 1770-1775	0.89 [0.80;1.00]	0.94 [0.69;1.26]
birth cohort 1775-1780	0.98 [0.87;1.09]	0.82 [0.61;1.09]
birth cohort 1780-1785	0.90 [0.80;1.01]	0.76 [0.57;1.01]
birth cohort 1785-1790	0.91 [0.82;1.02]	0.66 [0.49;0.86]
birth cohort 1790-1795	0.93 [0.84;1.03]	0.73 [0.57;0.95]
birth cohort 1795-1800	0.90 [0.81;1.00]	0.63 [0.50;0.81]
birth cohort 1800-1805	0.89 [0.81;0.99]	0.58 [0.46;0.75]
birth cohort 1805-1810	0.88 [0.79;0.97]	0.76 [0.59;0.97]
birth cohort 1810-1815	0.91 [0.83;1.00]	0.65 [0.51;0.82]
birth cohort 1815-1820	0.87 [0.80;0.96]	0.49 [0.39;0.63]
birth cohort 1820-1825	0.83 [0.76;0.91]	0.59 [0.47;0.74]
birth cohort 1825-1830	0.81 [0.74;0.89]	0.58 [0.46;0.73]
birth cohort 1830-1835	0.83 [0.76;0.92]	0.57 [0.45;0.73]
Intercept	5.23 [4.49;6.07]	0.72 [0.49;1.05]
last born	0.96 [0.91;1.00]	1.08 [0.96;1.23]
male	1.08 [1.05;1.12]	1.32 [1.20;1.44]
maternal loss 0-1	1.10 [0.94;1.28]	4.85 [3.46;6.97]
maternal loss 1-5	0.98 [0.89;1.07]	1.80 [1.44;2.27]
maternal loss 10-15	1.03 [0.95;1.11]	1.60 [1.30;1.99]
maternal loss 15-20	1.00 [0.93;1.09]	1.37 [1.11;1.68]
maternal loss 20-25	1.00 [0.93;1.08]	1.30 [1.05;1.59]
maternal loss 25-30	0.98 [0.92;1.05]	1.21 [1.01;1.45]
maternal loss 30-35	0.95 [0.89;1.01]	1.24 [1.05;1.47]
maternal loss 35-40	0.97 [0.91;1.02]	1.07 [0.92;1.26]
maternal loss 40-45	0.97 [0.91;1.03]	1.32 [1.12;1.56]
maternal loss 5-10	1.07 [0.99;1.16]	1.62 [1.32;1.99]
maternalage factor 14-20	0.95 [0.79;1.13]	1.27 [0.80;2.00]
maternalage factor 35-50	1.00 [0.95;1.05]	1.14 [0.99;1.31]
nr siblings	1.01 [1.00;1.02]	1.11 [1.07;1.15]
older siblings 1	1.03 [0.97;1.08]	0.99 [0.85;1.15]
older siblings 2	0.95 [0.89;1.02]	0.86 [0.71;1.04]
older siblings 3	0.93 [0.84;1.02]	0.83 [0.65;1.06]
older siblings 4	0.91 [0.81;1.02]	0.80 [0.58;1.08]

Effect	Hurdle Odds ratio	Zero-truncated Poisson Hazard ratio
older siblings 5+	0.91 [0.78;1.05]	0.57 [0.38;0.85]
paternal loss 0-1	0.86 [0.74;0.99]	1.78 [1.26;2.55]
paternal loss 1-5	0.96 [0.87;1.06]	1.70 [1.32;2.21]
paternal loss 10-15	1.01 [0.93;1.09]	1.17 [0.95;1.45]
paternal loss 15-20	0.91 [0.84;0.98]	1.11 [0.92;1.35]
paternal loss 20-25	0.89 [0.82;0.96]	1.17 [0.96;1.41]
paternal loss 25-30	0.99 [0.92;1.06]	1.06 [0.88;1.27]
paternal loss 30-35	0.97 [0.91;1.04]	0.98 [0.82;1.17]
paternal loss 35-40	0.99 [0.93;1.05]	0.98 [0.83;1.18]
paternal loss 40-45	0.99 [0.92;1.06]	1.15 [0.95;1.39]
paternal loss 5-10	0.93 [0.86;1.02]	1.21 [0.97;1.51]
paternalage	1.07 [0.97;1.19]	1.30 [0.99;1.69]
paternalage mean	0.93 [0.83;1.04]	0.84 [0.64;1.11]

2.3.5 Paternal age effect

This is the effect of 10 years of paternal age within families on number of children, combined over the hurdle and Zero-truncated Poisson component, expressed as a change in percentage `((predicted value at t + 10y)/(predicted value at t)) - 1`.

effect	median_estimate	ci_95	ci_80
percentage change	-8.41	[-24.83; 12.03]	[-19.50; 3.89]

2.3.5.1 Marginal effect plot

Paternal age effect on number of children The shaded areas show the 95% and 80% credibility intervals for the reference individuals and include uncertainty related to covariate effect sizes.

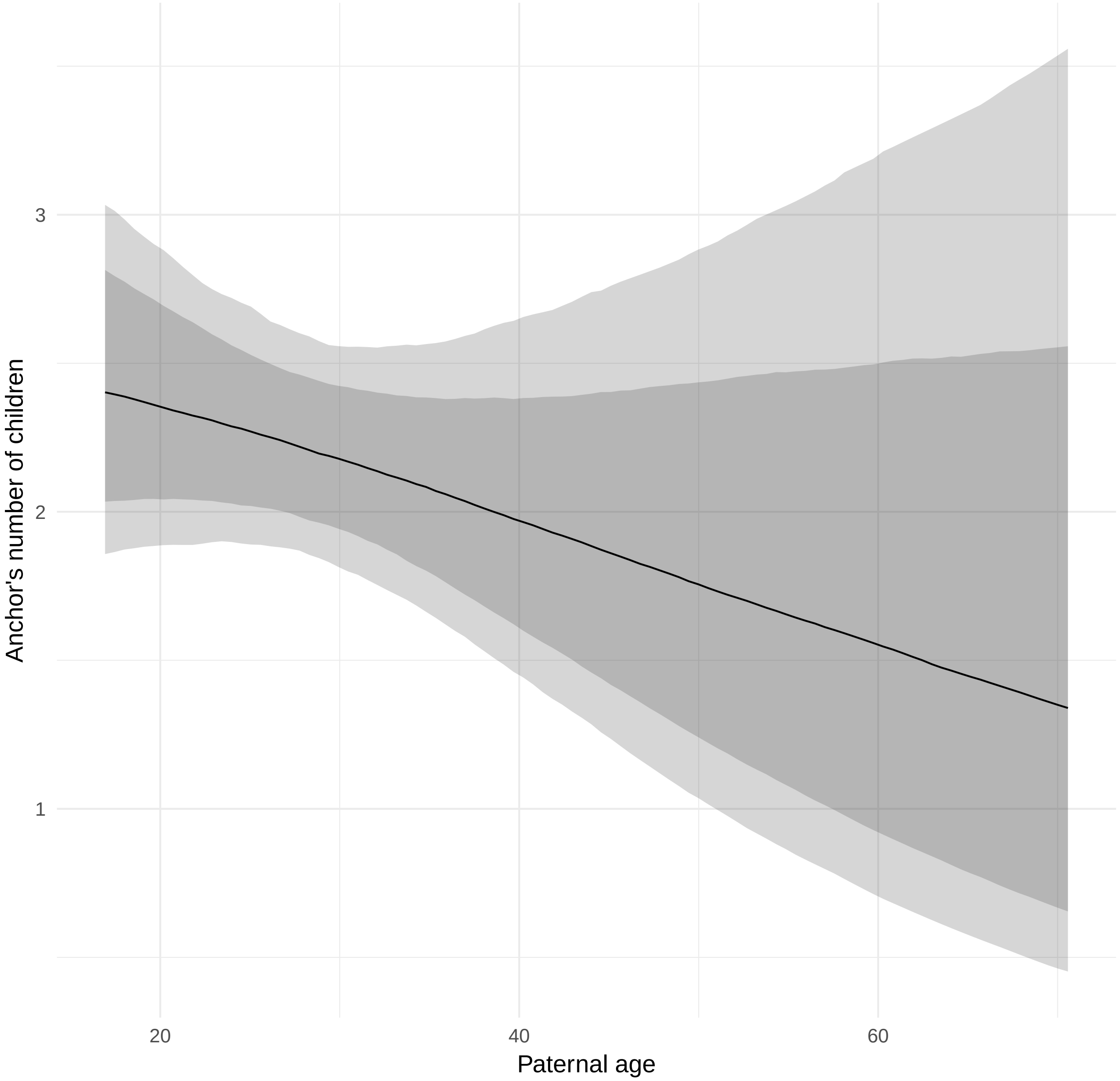

2.4 *m4*: Sibling comparison, nonlinear paternal age effect

Here, we compared siblings by including a random intercept for the family, and we modelled a possibly nonlinear effect for paternal age differences among siblings.

2.4.1 Model summary

Data: 9447 individuals nested in 2186 mother-father dyads.

Formula (Wilkinson notation): children ~ s(paternalage) + birth_cohort + male + maternalage.factor + paternalage.mean + paternal_loss + maternal_loss + older_siblings + nr.siblings + last_born + (1 | idParents).

- **family**: hurdle_poisson
- **link**: log

2.4.2 Priors

prior	class
normal(0,5)	b
student_t(3, 0, 5)	sd
student_t(3, 0, 10)	sds

2.4.3 Group-level effects

Component	Effect	Hurdle Estimate	Zero-truncated Poisson Estimate
idParents	sd(Intercept)	0.47 [0.38;0.56]	0.22 [0.20;0.25]

2.4.3.1 Splines

Effect	Hurdle Estimate	Zero-truncated Poisson Estimate
sds(spaternalage_1)	0.65 [0.02;2.32]	0.26 [0.01;0.93]

2.4.4 Population-level effects

Effect	Hurdle Odds ratio	Zero-truncated Poisson Hazard ratio
birth cohort 1760-1765	1.00 [0.88;1.12]	0.95 [0.67;1.33]
birth cohort 1765-1770	0.89 [0.79;0.99]	0.73 [0.55;0.98]
birth cohort 1770-1775	0.89 [0.80;1.00]	0.94 [0.70;1.25]
birth cohort 1775-1780	0.97 [0.88;1.09]	0.82 [0.61;1.08]
birth cohort 1780-1785	0.89 [0.80;1.00]	0.76 [0.57;1.02]
birth cohort 1785-1790	0.91 [0.82;1.01]	0.66 [0.49;0.87]
birth cohort 1790-1795	0.93 [0.84;1.03]	0.73 [0.55;0.96]
birth cohort 1795-1800	0.90 [0.82;0.99]	0.63 [0.49;0.81]
birth cohort 1800-1805	0.89 [0.81;0.98]	0.58 [0.45;0.74]
birth cohort 1805-1810	0.87 [0.79;0.96]	0.76 [0.60;0.98]
birth cohort 1810-1815	0.91 [0.83;0.99]	0.64 [0.50;0.82]

Effect	Hurdle Odds ratio	Zero-truncated Poisson Hazard ratio
birth cohort 1815-1820	0.87 [0.79;0.94]	0.49 [0.39;0.62]
birth cohort 1820-1825	0.83 [0.76;0.91]	0.59 [0.46;0.74]
birth cohort 1825-1830	0.81 [0.74;0.88]	0.58 [0.46;0.72]
birth cohort 1830-1835	0.83 [0.76;0.91]	0.58 [0.45;0.73]
Intercept	6.45 [4.37;9.53]	1.74 [0.58;4.99]
last born	0.96 [0.92;1.00]	1.08 [0.97;1.22]
male	1.08 [1.05;1.12]	1.32 [1.20;1.44]
maternal loss 0-1	1.10 [0.95;1.29]	4.83 [3.38;7.03]
maternal loss 1-5	0.98 [0.89;1.07]	1.79 [1.42;2.27]
maternal loss 10-15	1.03 [0.95;1.11]	1.59 [1.29;1.96]
maternal loss 15-20	1.00 [0.92;1.08]	1.35 [1.10;1.66]
maternal loss 20-25	1.00 [0.93;1.08]	1.29 [1.05;1.58]
maternal loss 25-30	0.98 [0.91;1.05]	1.20 [1.00;1.43]
maternal loss 30-35	0.95 [0.89;1.01]	1.24 [1.05;1.48]
maternal loss 35-40	0.97 [0.91;1.02]	1.06 [0.91;1.25]
maternal loss 40-45	0.97 [0.91;1.03]	1.31 [1.12;1.54]
maternal loss 5-10	1.07 [0.99;1.16]	1.61 [1.31;1.98]
maternalage factor 14-20	0.95 [0.79;1.13]	1.26 [0.80;1.97]
maternalage factor 35-50	1.00 [0.94;1.05]	1.14 [0.99;1.31]
nr siblings	1.01 [0.99;1.02]	1.11 [1.07;1.15]
older siblings 1	1.03 [0.98;1.08]	1.00 [0.86;1.16]
older siblings 2	0.96 [0.89;1.03]	0.88 [0.72;1.06]
older siblings 3	0.94 [0.86;1.03]	0.85 [0.67;1.09]
older siblings 4	0.92 [0.82;1.03]	0.82 [0.60;1.11]
older siblings 5+	0.92 [0.79;1.07]	0.58 [0.39;0.86]
paternal loss 0-1	0.86 [0.74;1.00]	1.76 [1.24;2.54]
paternal loss 1-5	0.96 [0.87;1.06]	1.69 [1.31;2.19]
paternal loss 10-15	1.01 [0.93;1.08]	1.17 [0.95;1.44]
paternal loss 15-20	0.91 [0.84;0.98]	1.10 [0.90;1.35]
paternal loss 20-25	0.89 [0.82;0.95]	1.16 [0.96;1.41]
paternal loss 25-30	0.99 [0.93;1.06]	1.06 [0.88;1.27]
paternal loss 30-35	0.97 [0.91;1.04]	0.98 [0.82;1.17]
paternal loss 35-40	0.99 [0.93;1.06]	0.99 [0.83;1.18]
paternal loss 40-45	0.99 [0.92;1.06]	1.16 [0.95;1.40]
paternal loss 5-10	0.93 [0.86;1.01]	1.21 [0.97;1.50]
paternalage mean	0.94 [0.85;1.04]	0.84 [0.64;1.12]
spaternalage	1.02 [0.88;1.14]	1.20 [0.87;1.66]

2.4.5 Paternal age effect

This is the effect of 10 years of paternal age within families on number of children, combined over the hurdle and Zero-truncated Poisson component, expressed as a change in percentage `((predicted value at t + 10y)/(predicted value at t)) - 1`.

effect	median_estimate	ci_95	ci_80
percentage change	-6.74	[-25.17; 14.85]	[-19.34; 6.80]

2.4.5.1 Marginal effect plot

Paternal age effect on number of children The shaded areas show the 95% and 80% credibility intervals for the reference individuals and include uncertainty related to covariate effect sizes.

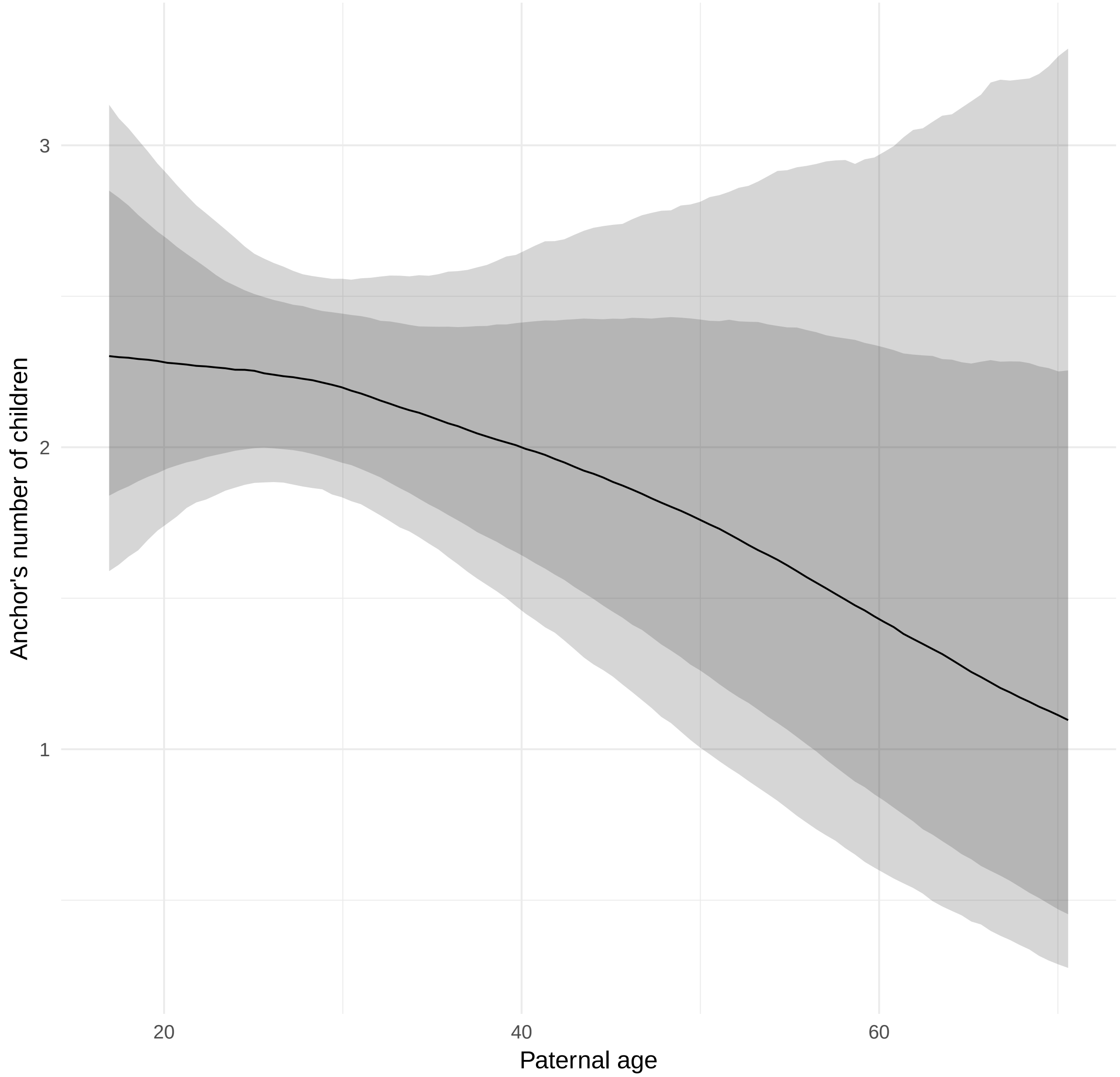

2.5 Main model comparison

We compare the four models using an approximate leave-one-out cross-validation information criterion as implemented in `brms` and `loo` and the Watanabe-Akaike information criterion.

2.5.1 Approximate leave-one-out (LOO) cross-validation

	LOOIC	SE
**m1**	30344	288.3
**m2**	30240	285
**m3**	30237	285.1
**m4**	30243	285.1
**m1 - m2**	104.1	40.85
**m1 - m3**	107.1	40.81
**m1 - m4**	101.2	40.9
**m2 - m3**	3.03	5.65
**m2 - m4**	-2.88	6.18
**m3 - m4**	-5.91	3.86

2.5.2 Watanabe-Akaike information criterion

	WAIC	SE
**m1**	30344	288.3
**m2**	30201	284.2
**m3**	30198	284.2
**m4**	30204	284.2
**m1 - m2**	143.2	41.13
**m1 - m3**	145.9	41.11
**m1 - m4**	140.3	41.21
**m2 - m3**	2.72	5.32
**m2 - m4**	-2.85	5.86
**m3 - m4**	-5.57	3.47

2.6 *e1*: Selective episode: offspring survival of the first year

In the first selective episode model, we tested how much of the paternal age effect happens in the first selective episode, i.e. in the offspring's survival of the first year.

2.6.1 Model summary

Data: 9447 individuals nested in 2186 mother-father dyads.

Formula (Wilkinson notation): survive1y ~ paternalage + birth_cohort + male + maternalage.factor + paternalage.mean + paternal_loss + maternal_loss + older_siblings + nr.siblings + last_born + (1 | idParents).

- **family**: bernoulli
- **link**: cauchit

2.6.2 Priors

prior	class
normal(0,5)	b
student_t(3, 0, 5)	sd

2.6.3 Group-level effects

Component	Effect	Zero-truncated Poisson Estimate
idParents	sd(Intercept)	0.84 [0.53;1.10]

2.6.4 Population-level effects

Effect	Hurdle Odds ratio
birth cohort 1760-1765	0.96 [0.44; 2.29]
birth cohort 1765-1770	0.84 [0.39; 1.82]
birth cohort 1770-1775	1.02 [0.46; 2.29]
birth cohort 1775-1780	0.63 [0.33; 1.18]
birth cohort 1780-1785	0.67 [0.33; 1.30]
birth cohort 1785-1790	0.86 [0.41; 1.98]
birth cohort 1790-1795	1.72 [0.75; 4.36]
birth cohort 1795-1800	0.68 [0.36; 1.26]
birth cohort 1800-1805	1.46 [0.69; 3.26]
birth cohort 1805-1810	0.60 [0.32; 1.08]
birth cohort 1810-1815	0.90 [0.48; 1.68]
birth cohort 1815-1820	2.01 [0.93; 4.60]
birth cohort 1820-1825	2.42 [1.06; 6.23]
birth cohort 1825-1830	2.32 [1.05; 5.39]
birth cohort 1830-1835	1.38 [0.70; 2.83]
Intercept	56.94 [16.78;189.11]
last born	0.94 [0.65; 1.40]
male	0.71 [0.54; 0.92]
maternal loss 0-1	0.08 [0.04; 0.14]

Effect	Hurdle Odds ratio
maternal loss 1-5	0.49 [0.25; 1.00]
maternal loss 10-15	0.76 [0.39; 1.55]
maternal loss 15-20	0.86 [0.45; 1.84]
maternal loss 20-25	0.82 [0.45; 1.54]
maternal loss 25-30	1.12 [0.62; 2.19]
maternal loss 30-35	0.67 [0.41; 1.13]
maternal loss 35-40	0.72 [0.44; 1.21]
maternal loss 40-45	0.78 [0.46; 1.35]
maternal loss 5-10	0.62 [0.32; 1.23]
maternalage factor 14-20	1.02 [0.39; 3.59]
maternalage factor 35-50	0.75 [0.48; 1.17]
nr siblings	0.72 [0.66; 0.78]
older siblings 1	1.66 [1.10; 2.53]
older siblings 2	2.26 [1.36; 3.82]
older siblings 3	3.37 [1.81; 6.43]
older siblings 4	3.79 [1.87; 8.22]
older siblings 5+	10.79 [3.96; 31.27]
paternal loss 0-1	0.34 [0.14; 0.94]
paternal loss 1-5	0.41 [0.21; 0.81]
paternal loss 10-15	0.63 [0.34; 1.19]
paternal loss 15-20	0.51 [0.29; 0.89]
paternal loss 20-25	0.74 [0.40; 1.31]
paternal loss 25-30	0.73 [0.40; 1.29]
paternal loss 30-35	0.91 [0.52; 1.65]
paternal loss 35-40	0.93 [0.52; 1.71]
paternal loss 40-45	0.46 [0.27; 0.79]
paternal loss 5-10	0.77 [0.37; 1.65]
paternalage	0.46 [0.23; 0.92]
paternalage mean	2.50 [1.19; 5.14]

2.6.5 Paternal age effect

This is the effect of 10 years of paternal age within families on probability of survival of the first year, combined over the hurdle and Zero-truncated Poisson component, expressed as a change in percentage `((predicted value at t + 10y)/(predicted value at t)) - 1`.

effect	median_estimate	ci_95	ci_80
percentage change	-2.15	[-5.35;-0.21]	[-4.03;-0.82]

2.6.5.1 Marginal effect plot

Paternal age effect on probability of survival of the first year The shaded areas show the 95% and 80% credibility intervals for the reference individuals and include uncertainty related to covariate effect sizes.

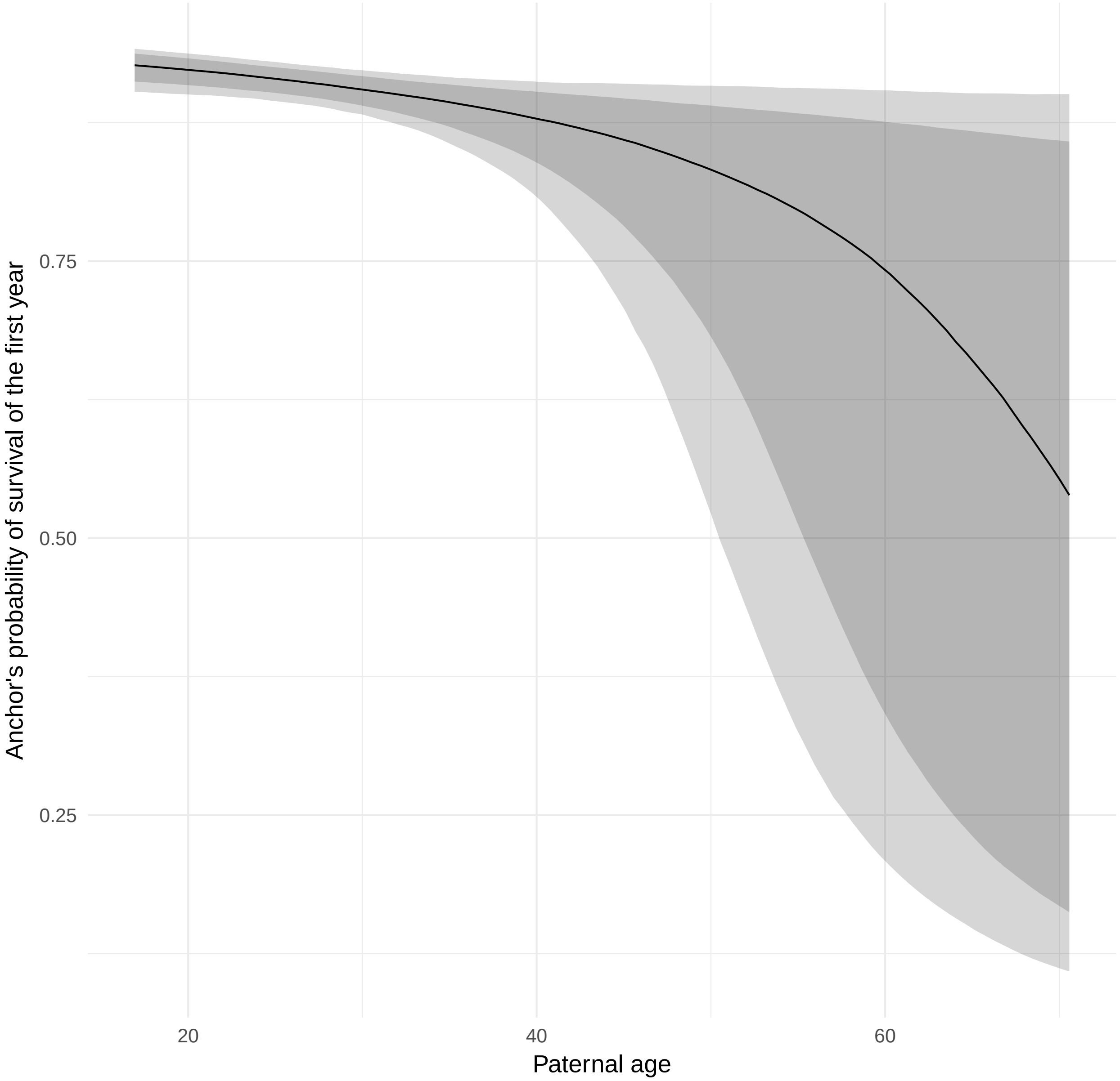

3 Québec

3.1 *m1*: No sibling comparison

Here, we ignore the pedigree structure of the data to see whether it matters for the estimation of the paternal age effect.

3.1.1 Model summary

Data: 68724 individuals.

Formula (Wilkinson notation): children ~ paternalage + birth_cohort + male + maternalage.factor + paternal_loss + maternal_loss + older_siblings + nr.siblings + last_born.

- **family**: hurdle_poisson
- **link**: log

3.1.2 Priors

prior	class
normal(0,5)	b

3.1.3 Population-level effects

Effect	Hurdle Odds ratio	Zero-truncated Poisson Hazard ratio
birth cohort 1675-1680	1.01 [0.98;1.04]	1.07 [0.94;1.21]
birth cohort 1680-1685	1.03 [1.01;1.06]	1.22 [1.08;1.38]
birth cohort 1685-1690	1.04 [1.01;1.07]	1.35 [1.19;1.53]
birth cohort 1690-1695	1.03 [1.01;1.06]	1.08 [0.96;1.21]
birth cohort 1695-1700	1.03 [1.00;1.05]	1.10 [0.98;1.23]
birth cohort 1700-1705	1.01 [0.99;1.04]	1.26 [1.13;1.40]
birth cohort 1705-1710	0.99 [0.97;1.02]	1.15 [1.03;1.28]
birth cohort 1710-1715	0.99 [0.96;1.01]	1.48 [1.33;1.64]
birth cohort 1715-1720	0.96 [0.93;0.98]	1.32 [1.19;1.47]
birth cohort 1720-1725	0.96 [0.94;0.98]	1.35 [1.22;1.50]
birth cohort 1725-1730	0.94 [0.91;0.96]	1.89 [1.70;2.09]
birth cohort 1730-1735	0.96 [0.93;0.98]	1.98 [1.80;2.18]
birth cohort 1735-1740	0.95 [0.92;0.97]	1.70 [1.54;1.87]
Intercept	8.47 [8.20;8.75]	0.42 [0.37;0.48]
last born	1.01 [0.99;1.02]	1.02 [0.97;1.08]
male	1.12 [1.11;1.13]	1.49 [1.45;1.54]
maternal loss 0-1	0.97 [0.93;1.01]	2.84 [2.50;3.24]
maternal loss 1-5	0.98 [0.96;1.01]	1.52 [1.41;1.65]
maternal loss 10-15	0.99 [0.97;1.01]	1.30 [1.21;1.40]
maternal loss 15-20	1.00 [0.98;1.02]	1.22 [1.13;1.30]
maternal loss 20-25	0.97 [0.96;0.99]	1.18 [1.10;1.26]
maternal loss 25-30	0.98 [0.96;0.99]	1.07 [1.00;1.14]
maternal loss 30-35	0.98 [0.97;1.00]	1.09 [1.03;1.15]
maternal loss 35-40	0.99 [0.98;1.00]	1.08 [1.02;1.15]
maternal loss 40-45	1.00 [0.98;1.01]	1.00 [0.95;1.06]

Effect	Hurdle Odds ratio	Zero-truncated Poisson Hazard ratio
maternal loss 5-10	1.01 [0.99;1.02]	1.34 [1.25;1.44]
maternal loss unclear	0.98 [0.96;0.99]	1.21 [1.15;1.28]
maternalage factor 14-20	0.99 [0.98;1.01]	1.00 [0.93;1.06]
maternalage factor 35-50	1.01 [1.00;1.02]	1.07 [1.02;1.12]
nr siblings	1.01 [1.01;1.01]	1.02 [1.01;1.02]
older siblings 1	0.98 [0.97;1.00]	0.95 [0.90;1.01]
older siblings 2	0.99 [0.97;1.00]	0.93 [0.87;0.99]
older siblings 3	0.98 [0.96;0.99]	0.89 [0.84;0.95]
older siblings 4	1.00 [0.98;1.01]	0.89 [0.83;0.95]
older siblings 5+	0.99 [0.97;1.00]	0.86 [0.81;0.92]
paternal loss 0-1	0.99 [0.96;1.03]	1.81 [1.59;2.06]
paternal loss 1-5	1.00 [0.98;1.02]	1.40 [1.27;1.53]
paternal loss 10-15	0.99 [0.97;1.00]	1.21 [1.12;1.31]
paternal loss 15-20	0.98 [0.96;0.99]	1.32 [1.23;1.42]
paternal loss 20-25	0.98 [0.96;0.99]	1.20 [1.12;1.28]
paternal loss 25-30	0.99 [0.97;1.00]	1.20 [1.13;1.28]
paternal loss 30-35	0.98 [0.96;0.99]	1.15 [1.08;1.22]
paternal loss 35-40	0.98 [0.96;0.99]	1.11 [1.04;1.18]
paternal loss 40-45	1.00 [0.98;1.01]	1.06 [0.99;1.14]
paternal loss 5-10	1.00 [0.98;1.02]	1.37 [1.27;1.49]
paternal loss unclear	0.96 [0.95;0.98]	1.42 [1.34;1.51]
paternalage	0.98 [0.98;0.99]	1.00 [0.97;1.02]

3.1.4 Paternal age effect

This is the effect of 10 years of paternal age within families on number of children, combined over the hurdle and Zero-truncated Poisson component, expressed as a change in percentage `((predicted value at t + 10y)/(predicted value at t)) - 1`.

effect	median_estimate	ci_95	ci_80
percentage change	-1.57	[-2.51;-0.59]	[-2.17;-0.90]

3.1.4.1 Marginal effect plot

Paternal age effect on number of children The shaded areas show the 95% and 80% credibility intervals for the reference individuals and include uncertainty related to covariate effect sizes.

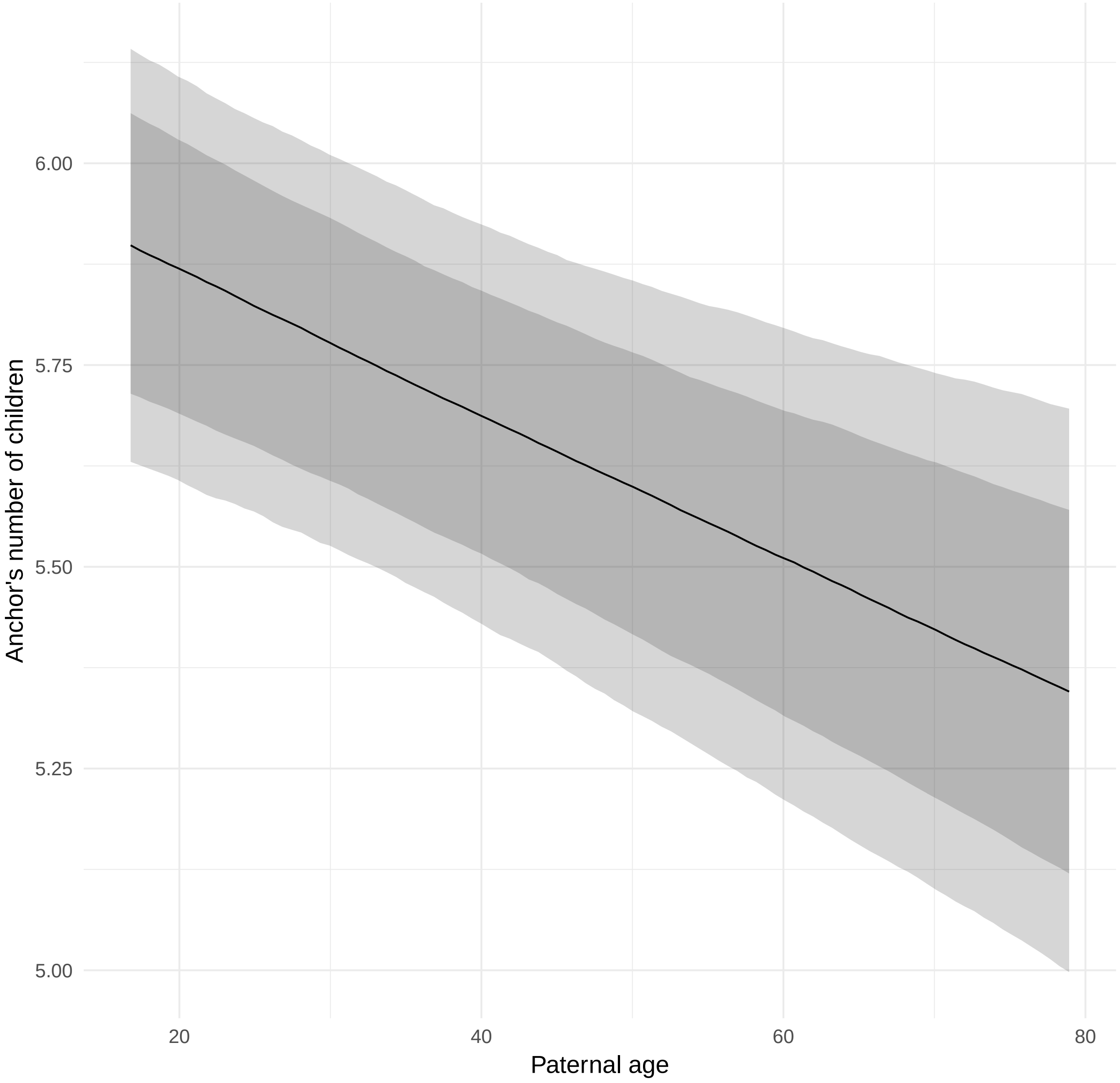

3.2 *m2*: Sibling comparison, no paternal age effect

Here, we compared siblings by including a random intercept for the family, but we modelled no effect for paternal age differences among siblings.

3.2.1 Model summary

Data: 68724 individuals nested in 12205 mother-father dyads.

Formula (Wilkinson notation): children ~ birth_cohort + male + maternalage.factor + paternalage.mean + paternal_loss + maternal_loss + older_siblings + nr.siblings + last_born + (1 | idParents).

- **family**: hurdle_poisson
- **link**: log

3.2.2 Priors

prior	class
normal(0,5)	b
student_t(3, 0, 5)	sd

3.2.3 Group-level effects

Component	Effect	Hurdle Estimate	Zero-truncated Poisson Estimate
idParents	sd(Intercept)	0.63 [0.60;0.66]	0.27 [0.27;0.28]

3.2.4 Population-level effects

Effect	Hurdle Odds ratio	Zero-truncated Poisson Hazard ratio
birth cohort 1675-1680	1.02 [0.99;1.05]	1.08 [0.94;1.26]
birth cohort 1680-1685	1.05 [1.02;1.08]	1.26 [1.10;1.45]
birth cohort 1685-1690	1.05 [1.02;1.09]	1.41 [1.23;1.62]
birth cohort 1690-1695	1.05 [1.02;1.08]	1.09 [0.96;1.26]
birth cohort 1695-1700	1.04 [1.00;1.07]	1.11 [0.98;1.27]
birth cohort 1700-1705	1.02 [0.99;1.06]	1.30 [1.14;1.48]
birth cohort 1705-1710	1.00 [0.97;1.03]	1.17 [1.03;1.33]
birth cohort 1710-1715	0.99 [0.96;1.02]	1.55 [1.37;1.77]
birth cohort 1715-1720	0.96 [0.93;1.00]	1.34 [1.19;1.53]
birth cohort 1720-1725	0.96 [0.93;0.99]	1.37 [1.21;1.55]
birth cohort 1725-1730	0.94 [0.91;0.97]	1.97 [1.75;2.24]
birth cohort 1730-1735	0.95 [0.93;0.98]	2.08 [1.85;2.35]
birth cohort 1735-1740	0.94 [0.92;0.97]	1.77 [1.58;2.00]
Intercept	8.26 [7.82;8.71]	0.43 [0.36;0.51]
last born	1.00 [0.99;1.02]	1.01 [0.95;1.07]
male	1.12 [1.11;1.13]	1.55 [1.50;1.60]
maternal loss 0-1	0.95 [0.91;1.00]	2.99 [2.59;3.45]
maternal loss 1-5	0.97 [0.95;1.00]	1.52 [1.38;1.67]
maternal loss 10-15	0.99 [0.97;1.02]	1.31 [1.21;1.43]
maternal loss 15-20	1.01 [0.98;1.03]	1.23 [1.14;1.34]

Effect	Hurdle Odds ratio	Zero-truncated Poisson Hazard ratio
maternal loss 20-25	0.98 [0.96;1.00]	1.20 [1.11;1.30]
maternal loss 25-30	0.99 [0.97;1.01]	1.07 [0.99;1.15]
maternal loss 30-35	0.99 [0.97;1.01]	1.10 [1.03;1.18]
maternal loss 35-40	1.00 [0.98;1.02]	1.09 [1.02;1.16]
maternal loss 40-45	1.00 [0.99;1.02]	0.99 [0.93;1.07]
maternal loss 5-10	1.01 [0.99;1.04]	1.32 [1.21;1.44]
maternal loss unclear	0.98 [0.96;1.01]	1.26 [1.17;1.36]
maternalage factor 14-20	0.99 [0.97;1.01]	1.04 [0.97;1.11]
maternalage factor 35-50	1.01 [0.99;1.02]	1.09 [1.04;1.14]
nr siblings	1.01 [1.00;1.01]	1.01 [1.01;1.02]
older siblings 1	0.98 [0.97;0.99]	0.96 [0.90;1.02]
older siblings 2	0.98 [0.96;0.99]	0.93 [0.87;1.00]
older siblings 3	0.97 [0.96;0.99]	0.90 [0.84;0.96]
older siblings 4	0.98 [0.97;1.00]	0.90 [0.84;0.97]
older siblings 5+	0.97 [0.96;0.99]	0.87 [0.82;0.93]
paternal loss 0-1	0.97 [0.93;1.01]	1.85 [1.61;2.13]
paternal loss 1-5	0.99 [0.96;1.02]	1.41 [1.27;1.57]
paternal loss 10-15	0.99 [0.96;1.01]	1.22 [1.12;1.33]
paternal loss 15-20	0.98 [0.96;1.00]	1.35 [1.24;1.47]
paternal loss 20-25	0.98 [0.96;1.00]	1.21 [1.12;1.30]
paternal loss 25-30	0.99 [0.97;1.01]	1.21 [1.12;1.31]
paternal loss 30-35	0.98 [0.96;1.00]	1.16 [1.08;1.25]
paternal loss 35-40	0.98 [0.96;1.00]	1.11 [1.03;1.19]
paternal loss 40-45	1.00 [0.98;1.02]	1.07 [0.99;1.16]
paternal loss 5-10	0.99 [0.97;1.02]	1.38 [1.26;1.52]
paternal loss unclear	0.95 [0.93;0.97]	1.47 [1.37;1.59]
paternalage mean	0.98 [0.97;0.99]	0.98 [0.95;1.01]

3.2.5 Paternal age effect

This is the effect of 10 years of paternal age within families on number of children, combined over the hurdle and Zero-truncated Poisson component, expressed as a change in percentage `((predicted value at t + 10y)/(predicted value at t)) - 1`.

This model did not contain a within family paternal age predictor.

3.3 *m3*: Sibling comparison, linear paternal age effect

Here, we compared siblings by including a random intercept for the family, and we modelled a linear effect for paternal age differences among siblings.

3.3.1 Model summary

Data: 68724 individuals nested in 12205 mother-father dyads.

Formula (Wilkinson notation): children ~ paternalage + birth_cohort + male + maternalage.factor + paternalage.mean + paternal_loss + maternal_loss + older_siblings + nr.siblings + last_born + (1 | idParents).

- **family**: hurdle_poisson
- **link**: log

3.3.2 Priors

prior	class
normal(0,5)	b
student_t(3, 0, 5)	sd

3.3.3 Group-level effects

Component	Effect	Hurdle Estimate	Zero-truncated Poisson Estimate
idParents	sd(Intercept)	0.63 [0.60;0.66]	0.27 [0.27;0.28]

3.3.4 Population-level effects

Effect	Hurdle Odds ratio	Zero-truncated Poisson Hazard ratio
birth cohort 1675-1680	1.02 [0.99;1.05]	1.08 [0.95;1.24]
birth cohort 1680-1685	1.05 [1.02;1.08]	1.26 [1.10;1.44]
birth cohort 1685-1690	1.05 [1.02;1.09]	1.40 [1.22;1.60]
birth cohort 1690-1695	1.05 [1.02;1.08]	1.08 [0.95;1.24]
birth cohort 1695-1700	1.04 [1.00;1.07]	1.10 [0.98;1.26]
birth cohort 1700-1705	1.02 [0.99;1.05]	1.29 [1.14;1.46]
birth cohort 1705-1710	1.00 [0.97;1.03]	1.17 [1.03;1.32]
birth cohort 1710-1715	0.99 [0.96;1.02]	1.54 [1.37;1.75]
birth cohort 1715-1720	0.96 [0.93;0.99]	1.33 [1.18;1.50]
birth cohort 1720-1725	0.96 [0.93;0.99]	1.36 [1.21;1.54]
birth cohort 1725-1730	0.94 [0.91;0.96]	1.95 [1.73;2.21]
birth cohort 1730-1735	0.95 [0.92;0.98]	2.06 [1.84;2.33]
birth cohort 1735-1740	0.94 [0.91;0.97]	1.76 [1.57;1.98]
Intercept	8.30 [7.86;8.77]	0.45 [0.38;0.53]
last born	1.00 [0.99;1.02]	1.00 [0.94;1.05]
male	1.12 [1.11;1.13]	1.55 [1.50;1.60]
maternal loss 0-1	0.95 [0.91;0.99]	2.88 [2.51;3.35]
maternal loss 1-5	0.97 [0.94;1.00]	1.48 [1.35;1.64]
maternal loss 10-15	0.99 [0.97;1.02]	1.29 [1.19;1.40]
maternal loss 15-20	1.01 [0.98;1.03]	1.22 [1.13;1.33]
maternal loss 20-25	0.98 [0.96;1.00]	1.19 [1.09;1.29]
maternal loss 25-30	0.99 [0.97;1.01]	1.05 [0.98;1.14]
maternal loss 30-35	0.99 [0.97;1.01]	1.09 [1.01;1.17]
maternal loss 35-40	1.00 [0.98;1.02]	1.08 [1.02;1.16]
maternal loss 40-45	1.00 [0.99;1.02]	0.99 [0.92;1.06]
maternal loss 5-10	1.01 [0.99;1.04]	1.29 [1.19;1.41]
maternal loss unclear	0.98 [0.96;1.01]	1.25 [1.16;1.34]
maternalage factor 14-20	0.99 [0.97;1.01]	1.05 [0.97;1.13]
maternalage factor 35-50	1.00 [0.99;1.02]	1.03 [0.97;1.09]
nr siblings	1.01 [1.00;1.01]	1.02 [1.01;1.03]
older siblings 1	0.98 [0.96;0.99]	0.94 [0.89;1.00]
older siblings 2	0.97 [0.96;0.99]	0.90 [0.84;0.96]
older siblings 3	0.97 [0.95;0.99]	0.85 [0.79;0.91]
older siblings 4	0.98 [0.96;1.00]	0.83 [0.76;0.90]
older siblings 5+	0.96 [0.94;0.99]	0.76 [0.69;0.85]

Effect	Hurdle Odds ratio	Zero-truncated Poisson Hazard ratio
paternal loss 0-1	0.96 [0.92;1.01]	1.78 [1.54;2.05]
paternal loss 1-5	0.98 [0.95;1.02]	1.37 [1.24;1.52]
paternal loss 10-15	0.98 [0.96;1.01]	1.19 [1.09;1.29]
paternal loss 15-20	0.98 [0.95;1.00]	1.33 [1.22;1.45]
paternal loss 20-25	0.98 [0.96;1.00]	1.19 [1.10;1.29]
paternal loss 25-30	0.99 [0.97;1.01]	1.20 [1.11;1.29]
paternal loss 30-35	0.98 [0.96;1.00]	1.15 [1.07;1.24]
paternal loss 35-40	0.98 [0.96;1.00]	1.11 [1.03;1.19]
paternal loss 40-45	1.00 [0.98;1.02]	1.07 [0.99;1.16]
paternal loss 5-10	0.99 [0.96;1.02]	1.34 [1.22;1.47]
paternal loss unclear	0.95 [0.92;0.97]	1.45 [1.34;1.57]
paternalage	1.01 [0.99;1.03]	1.14 [1.05;1.23]
paternalage mean	0.97 [0.95;0.99]	0.87 [0.80;0.94]

3.3.5 Paternal age effect

This is the effect of 10 years of paternal age within families on number of children, combined over the hurdle and Zero-truncated Poisson component, expressed as a change in percentage `((predicted value at t + 10y)/(predicted value at t)) - 1`.

effect	median_estimate	ci_95	ci_80
percentage change	-3.00	[-6.08; 0.24]	[-4.97;-0.90]

3.3.5.1 Marginal effect plot

Paternal age effect on number of children The shaded areas show the 95% and 80% credibility intervals for the reference individuals and include uncertainty related to covariate effect sizes.

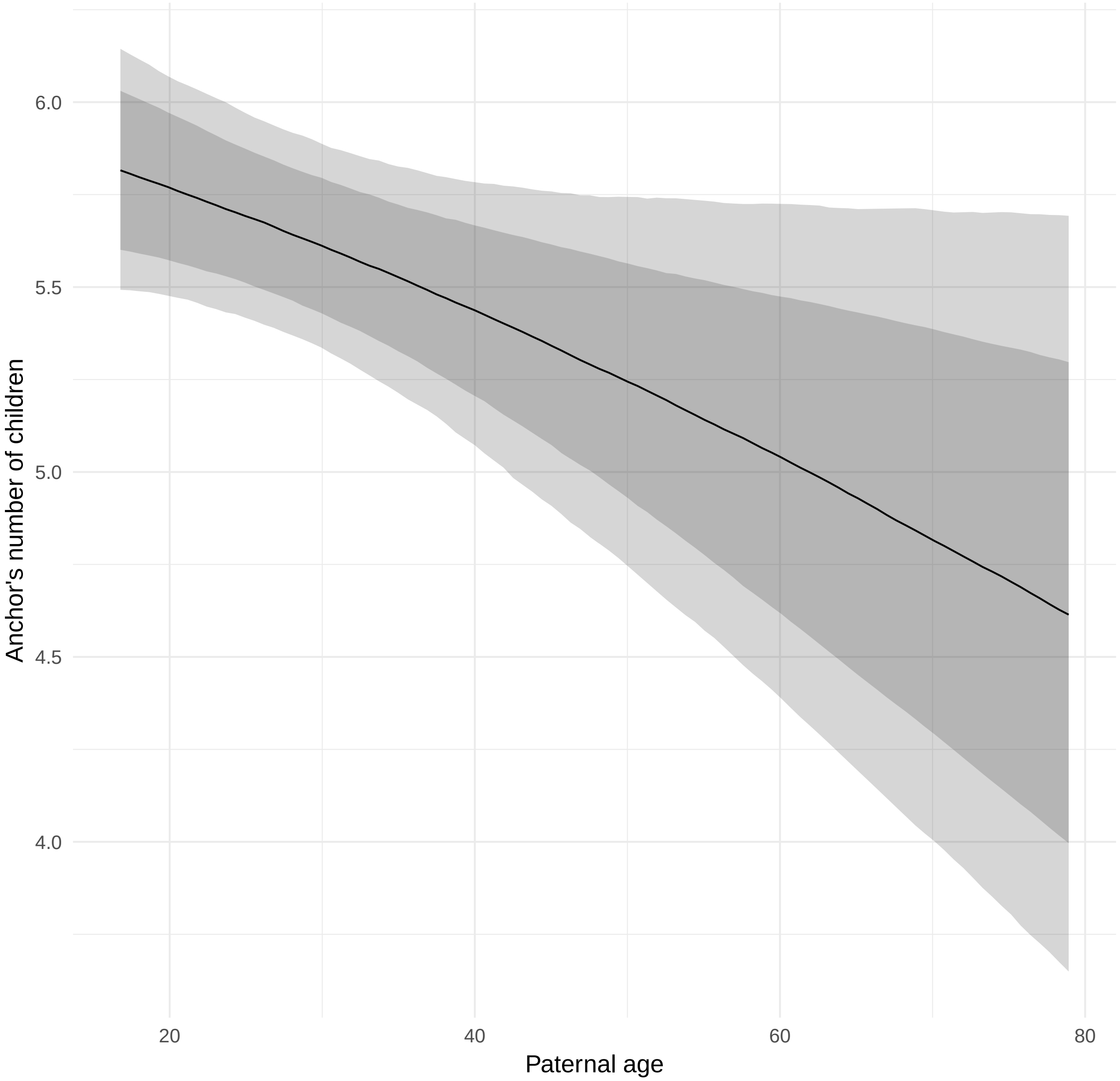

3.4 *m4*: Sibling comparison, nonlinear paternal age effect

Here, we compared siblings by including a random intercept for the family, and we modelled a possibly nonlinear effect for paternal age differences among siblings.

3.4.1 Model summary

Data: 68724 individuals nested in 12205 mother-father dyads.

Formula (Wilkinson notation): children ~ s(paternalage) + birth_cohort + male + maternalage.factor + paternalage.mean + paternal_loss + maternal_loss + older_siblings + nr.siblings + last_born + (1 | idParents).

- **family**: hurdle_poisson
- **link**: log

3.4.2 Priors

prior	class
normal(0,5)	b
student_t(3, 0, 5)	sd
student_t(3, 0, 10)	sds

3.4.3 Group-level effects

Component	Effect	Hurdle Estimate	Zero-truncated Poisson Estimate
idParents	sd(Intercept)	0.63 [0.60;0.65]	0.27 [0.27;0.28]

3.4.3.1 Splines

Effect	Hurdle Estimate	Zero-truncated Poisson Estimate
sds(spaternalage_1)	0.80 [0.24;1.97]	0.10 [0.00;0.32]

3.4.4 Population-level effects

Effect	Hurdle Odds ratio	Zero-truncated Poisson Hazard ratio
birth cohort 1675-1680	1.02 [0.99;1.05]	1.08 [0.95;1.23]
birth cohort 1680-1685	1.05 [1.02;1.08]	1.26 [1.10;1.44]
birth cohort 1685-1690	1.05 [1.02;1.09]	1.41 [1.23;1.62]
birth cohort 1690-1695	1.05 [1.02;1.08]	1.10 [0.97;1.26]
birth cohort 1695-1700	1.04 [1.00;1.07]	1.12 [0.99;1.28]
birth cohort 1700-1705	1.02 [0.99;1.05]	1.31 [1.16;1.48]
birth cohort 1705-1710	1.00 [0.97;1.03]	1.18 [1.04;1.34]
birth cohort 1710-1715	0.99 [0.96;1.02]	1.56 [1.38;1.76]
birth cohort 1715-1720	0.96 [0.93;0.99]	1.35 [1.20;1.52]
birth cohort 1720-1725	0.96 [0.93;0.99]	1.38 [1.23;1.55]
birth cohort 1725-1730	0.94 [0.91;0.96]	1.98 [1.77;2.23]

Effect	Hurdle Odds ratio	Zero-truncated Poisson Hazard ratio
birth cohort 1730-1735	0.95 [0.92;0.98]	2.09 [1.87;2.35]
birth cohort 1735-1740	0.94 [0.91;0.97]	1.78 [1.58;2.00]
Intercept	8.63 [7.82;9.56]	0.82 [0.58;1.18]
last born	1.00 [0.99;1.02]	1.00 [0.94;1.06]
male	1.12 [1.11;1.13]	1.55 [1.50;1.60]
maternal loss 0-1	0.95 [0.91;0.99]	2.89 [2.54;3.32]
maternal loss 1-5	0.97 [0.94;1.00]	1.48 [1.35;1.64]
maternal loss 10-15	0.99 [0.97;1.02]	1.30 [1.19;1.41]
maternal loss 15-20	1.01 [0.98;1.03]	1.22 [1.13;1.34]
maternal loss 20-25	0.98 [0.96;1.00]	1.19 [1.10;1.28]
maternal loss 25-30	0.99 [0.97;1.01]	1.05 [0.98;1.14]
maternal loss 30-35	0.99 [0.97;1.01]	1.09 [1.01;1.17]
maternal loss 35-40	1.00 [0.98;1.02]	1.08 [1.02;1.15]
maternal loss 40-45	1.00 [0.99;1.02]	0.99 [0.93;1.05]
maternal loss 5-10	1.01 [0.99;1.04]	1.30 [1.19;1.41]
maternal loss unclear	0.98 [0.96;1.01]	1.25 [1.16;1.35]
maternalage factor 14-20	0.99 [0.97;1.01]	1.06 [0.98;1.14]
maternalage factor 35-50	1.00 [0.99;1.02]	1.04 [0.98;1.10]
nr siblings	1.01 [1.00;1.01]	1.02 [1.01;1.03]
older siblings 1	0.98 [0.96;0.99]	0.92 [0.87;0.98]
older siblings 2	0.98 [0.96;0.99]	0.86 [0.81;0.93]
older siblings 3	0.97 [0.95;0.99]	0.80 [0.74;0.87]
older siblings 4	0.98 [0.96;1.00]	0.77 [0.71;0.84]
older siblings 5+	0.96 [0.94;0.99]	0.70 [0.63;0.78]
paternal loss 0-1	0.96 [0.92;1.00]	1.78 [1.56;2.05]
paternal loss 1-5	0.98 [0.95;1.01]	1.37 [1.23;1.52]
paternal loss 10-15	0.98 [0.96;1.01]	1.18 [1.08;1.29]
paternal loss 15-20	0.98 [0.95;1.00]	1.32 [1.21;1.43]
paternal loss 20-25	0.98 [0.96;1.00]	1.18 [1.09;1.27]
paternal loss 25-30	0.99 [0.97;1.01]	1.18 [1.09;1.27]
paternal loss 30-35	0.98 [0.96;1.00]	1.13 [1.06;1.22]
paternal loss 35-40	0.98 [0.96;1.00]	1.09 [1.02;1.17]
paternal loss 40-45	1.00 [0.98;1.02]	1.06 [0.98;1.14]
paternal loss 5-10	0.99 [0.96;1.02]	1.34 [1.22;1.47]
paternal loss unclear	0.95 [0.92;0.97]	1.44 [1.34;1.56]
paternalage mean	0.97 [0.95;0.99]	0.84 [0.77;0.91]
spaternalage	1.02 [0.99;1.10]	1.12 [0.89;1.41]

3.4.5 Paternal age effect

This is the effect of 10 years of paternal age within families on number of children, combined over the hurdle and Zero-truncated Poisson component, expressed as a change in percentage `((predicted value at t + 10y)/(predicted value at t)) - 1`.

effect	median_estimate	ci_95	ci_80
percentage change	-7.98	[-11.85; -4.12]	[-10.49; -5.23]

3.4.5.1 Marginal effect plot

Paternal age effect on number of children The shaded areas show the 95% and 80% credibility intervals for the reference individuals and include uncertainty related to covariate effect sizes.

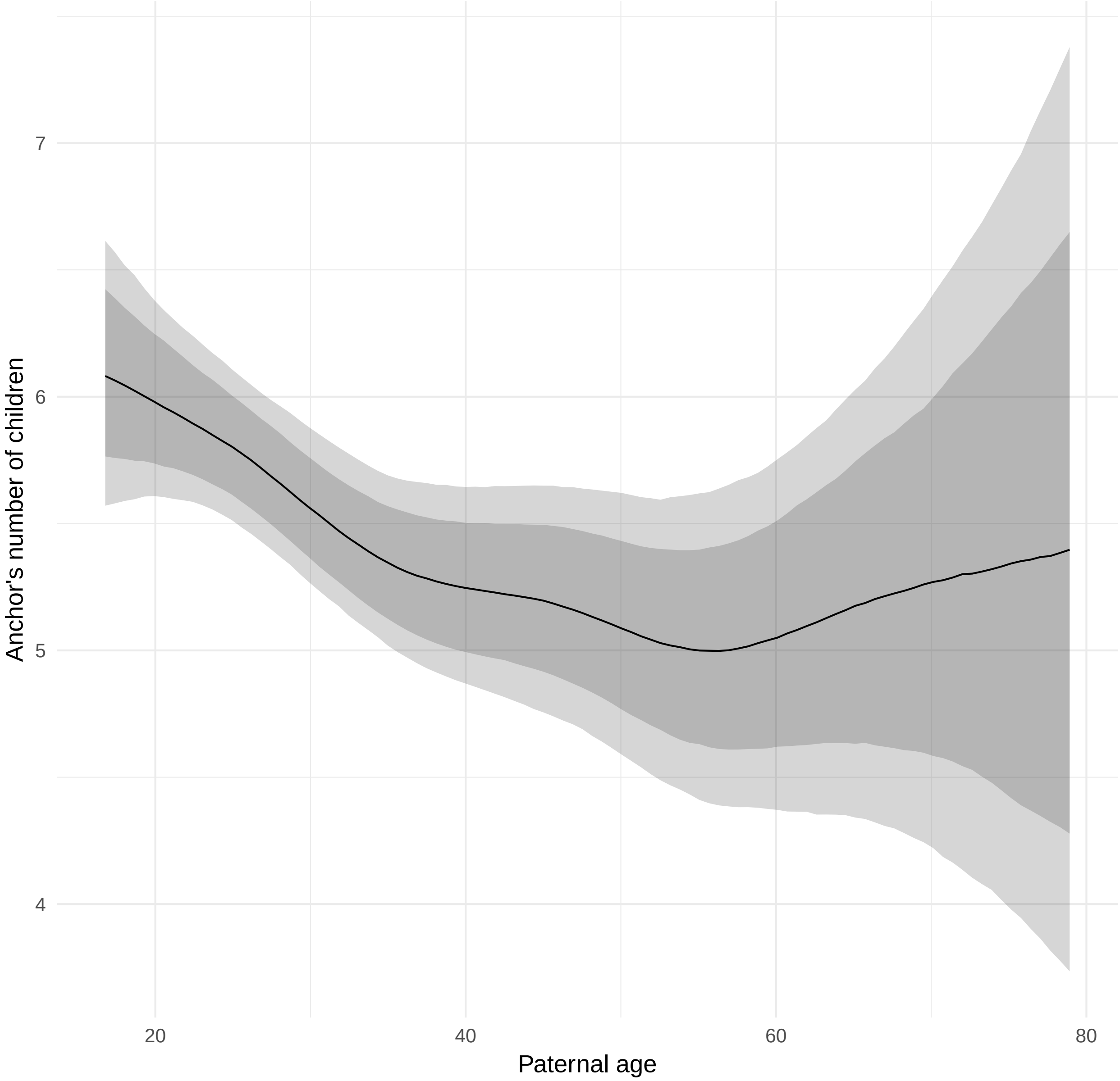

3.5 Main model comparison

We compare the four models using an approximate leave-one-out cross-validation information criterion as implemented in `brms` and `loo` and the Watanabe-Akaike information criterion.

3.5.1 Approximate leave-one-out (LOO) cross-validation

	LOOIC	SE
**m1**	299887	992.2
**m2**	293615	957.5
**m3**	293619	957.7
**m4**	293606	957.7
**m1 - m2**	6272	299.1
**m1 - m3**	6268	299.3
**m1 - m4**	6281	299.3
**m2 - m3**	-3.66	17.39
**m2 - m4**	9.83	19.87
**m3 - m4**	13.49	18.8

3.5.2 Watanabe-Akaike information criterion

	WAIC	SE
**m1**	299887	992.2
**m2**	292565	950.5
**m3**	292572	950.7
**m4**	292556	950.7
**m1 - m2**	7322	299.6
**m1 - m3**	7315	299.9
**m1 - m4**	7331	299.9
**m2 - m3**	-7.05	13.1
**m2 - m4**	8.81	16.28
**m3 - m4**	15.86	14.79

3.6 *e1*: Selective episode: offspring survival of the first year

In the first selective episode model, we tested how much of the paternal age effect happens in the first selective episode, i.e. in the offspring's survival of the first year.

3.6.1 Model summary

Data: 61493 individuals nested in 11940 mother-father dyads.

Formula (Wilkinson notation): survive1y ~ paternalage + birth_cohort + male + maternalage.factor + paternalage.mean + paternal_loss + maternal_loss + older_siblings + nr.siblings + last_born + (1 | idParents).

- **family**: bernoulli
- **link**: cauchit

3.6.2 Priors

prior	class
normal(0,5)	b
student_t(3, 0, 5)	sd

3.6.3 Group-level effects

Component	Effect	Zero-truncated Poisson Estimate
idParents	sd(Intercept)	1.06 [1.00;1.12]

3.6.4 Population-level effects

Effect	Hurdle Odds ratio
birth cohort 1675-1680	1.22 [0.69; 2.17]
birth cohort 1680-1685	0.57 [0.34; 0.92]
birth cohort 1685-1690	0.23 [0.15; 0.35]
birth cohort 1690-1695	0.43 [0.28; 0.67]
birth cohort 1695-1700	0.44 [0.28; 0.66]
birth cohort 1700-1705	0.27 [0.17; 0.41]
birth cohort 1705-1710	0.46 [0.30; 0.70]
birth cohort 1710-1715	0.24 [0.15; 0.36]
birth cohort 1715-1720	0.28 [0.18; 0.41]
birth cohort 1720-1725	0.28 [0.18; 0.41]
birth cohort 1725-1730	0.19 [0.13; 0.28]
birth cohort 1730-1735	0.15 [0.10; 0.23]
birth cohort 1735-1740	0.19 [0.12; 0.28]
Intercept	42.30 [25.95;69.83]
last born	1.00 [0.90; 1.10]
male	0.66 [0.62; 0.70]
maternal loss 0-1	0.11 [0.09; 0.13]
maternal loss 1-5	0.50 [0.42; 0.59]
maternal loss 10-15	0.67 [0.57; 0.78]

Effect	Hurdle Odds ratio
maternal loss 15-20	0.68 [0.59; 0.79]
maternal loss 20-25	0.76 [0.66; 0.89]
maternal loss 25-30	0.85 [0.73; 0.97]
maternal loss 30-35	0.81 [0.71; 0.92]
maternal loss 35-40	0.79 [0.70; 0.89]
maternal loss 40-45	0.97 [0.85; 1.11]
maternal loss 5-10	0.64 [0.55; 0.76]
maternal loss unclear	0.76 [0.66; 0.88]
maternalage factor 14-20	0.84 [0.73; 0.96]
maternalage factor 35-50	0.92 [0.82; 1.02]
nr siblings	0.91 [0.90; 0.92]
older siblings 1	1.56 [1.39; 1.75]
older siblings 2	1.72 [1.52; 1.94]
older siblings 3	2.21 [1.92; 2.56]
older siblings 4	2.29 [1.96; 2.67]
older siblings 5+	2.78 [2.33; 3.32]
paternal loss 0-1	0.36 [0.29; 0.45]
paternal loss 1-5	0.62 [0.51; 0.75]
paternal loss 10-15	0.80 [0.68; 0.94]
paternal loss 15-20	0.72 [0.62; 0.84]
paternal loss 20-25	0.86 [0.74; 0.99]
paternal loss 25-30	0.78 [0.68; 0.90]
paternal loss 30-35	0.83 [0.72; 0.95]
paternal loss 35-40	0.85 [0.75; 0.97]
paternal loss 40-45	0.97 [0.83; 1.12]
paternal loss 5-10	0.70 [0.59; 0.84]
paternal loss unclear	0.70 [0.60; 0.81]
paternalage	0.64 [0.56; 0.74]
paternalage mean	1.72 [1.49; 1.99]

3.6.5 Paternal age effect

This is the effect of 10 years of paternal age within families on probability of survival of the first year, combined over the hurdle and Zero-truncated Poisson component, expressed as a change in percentage `((predicted value at t + 10y)/(predicted value at t)) - 1`.

effect	median_estimate	ci_95	ci_80
percentage change	-1.03	[-1.51;-0.67]	[-1.32;-0.78]

3.6.5.1 Marginal effect plot

Paternal age effect on probability of survival of the first year The shaded areas show the 95% and 80% credibility intervals for the reference individuals and include uncertainty related to covariate effect sizes.

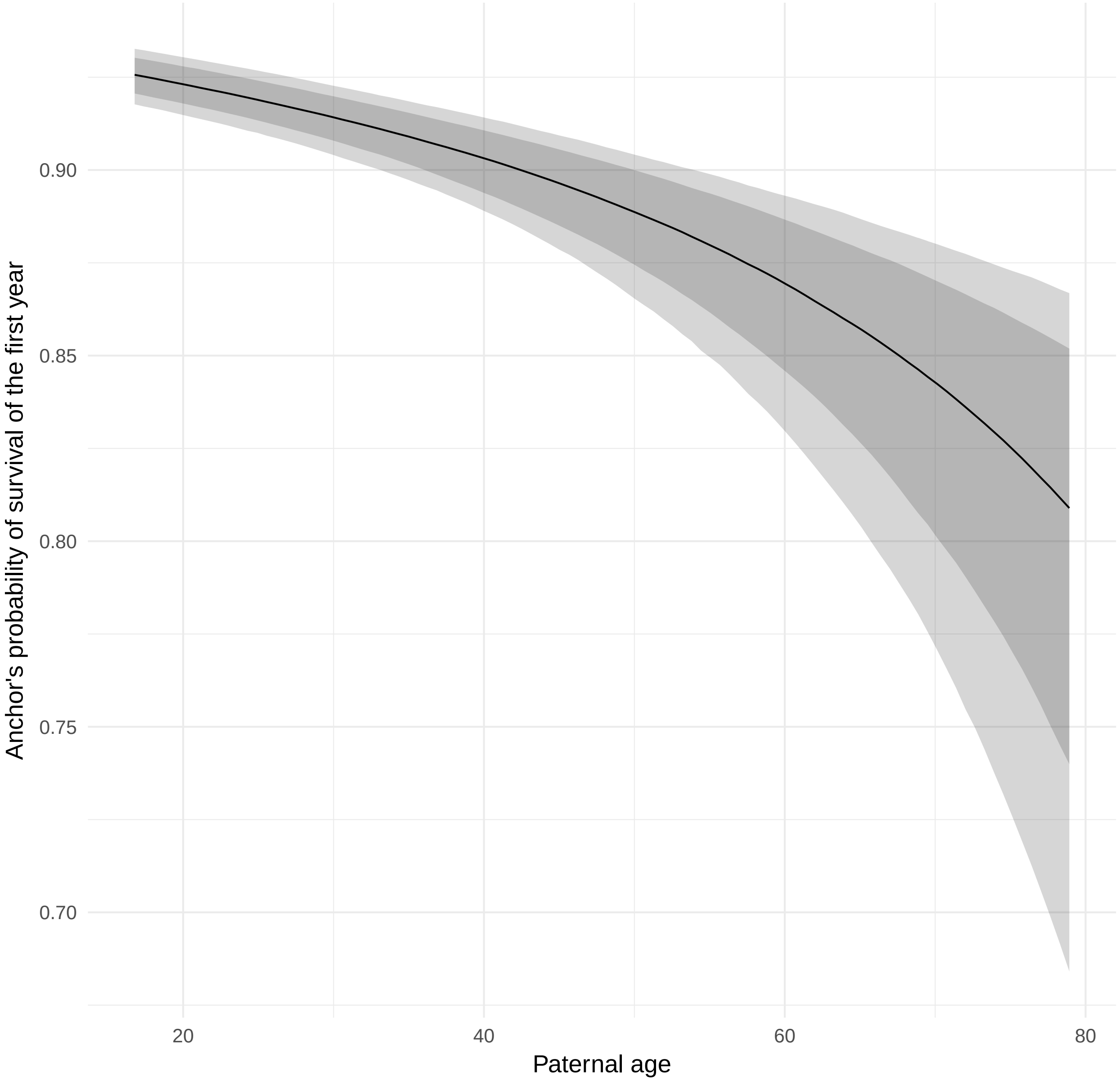

4 Historical Sweden

4.1 *m1*: No sibling comparison

Here, we ignore the pedigree structure of the data to see whether it matters for the estimation of the paternal age effect.

4.1.1 Model summary

Data: 56663 individuals.

Formula (Wilkinson notation): children ~ paternalage + birth_cohort + male + maternalage.factor + paternal_loss + maternal_loss + older_siblings + nr.siblings + last_born.

- **family**: hurdle_poisson
- **link**: log

4.1.2 Priors

prior	class
normal(0,5)	b

4.1.3 Population-level effects

Effect	Hurdle Odds ratio	Zero-truncated Poisson Hazard ratio
birth cohort 1750-1755	0.91 [0.75;1.10]	1.40 [0.87;2.28]
birth cohort 1755-1760	1.11 [0.96;1.29]	1.12 [0.77;1.65]
birth cohort 1760-1765	1.21 [1.07;1.37]	1.02 [0.72;1.44]
birth cohort 1765-1770	1.15 [1.01;1.30]	0.78 [0.56;1.11]
birth cohort 1770-1775	1.13 [0.99;1.28]	0.96 [0.68;1.36]
birth cohort 1775-1780	1.09 [0.97;1.24]	1.12 [0.82;1.55]
birth cohort 1780-1785	1.20 [1.06;1.36]	1.05 [0.76;1.45]
birth cohort 1785-1790	1.19 [1.06;1.34]	1.17 [0.87;1.61]
birth cohort 1790-1795	1.08 [0.97;1.22]	1.41 [1.06;1.87]
birth cohort 1795-1800	1.11 [1.00;1.24]	1.17 [0.89;1.55]
birth cohort 1800-1805	1.05 [0.94;1.18]	1.12 [0.85;1.48]
birth cohort 1805-1810	1.07 [0.96;1.20]	1.06 [0.80;1.41]
birth cohort 1810-1815	1.09 [0.97;1.21]	1.16 [0.89;1.53]
birth cohort 1815-1820	1.16 [1.04;1.29]	0.98 [0.75;1.28]
birth cohort 1820-1825	1.16 [1.05;1.29]	0.89 [0.68;1.16]
birth cohort 1825-1830	1.12 [1.01;1.24]	0.88 [0.68;1.15]
birth cohort 1830-1835	1.14 [1.03;1.27]	0.90 [0.69;1.18]
birth cohort 1835-1840	1.13 [1.02;1.26]	0.90 [0.69;1.17]
birth cohort 1840-1845	1.11 [1.00;1.23]	0.91 [0.70;1.19]
birth cohort 1845-1850	1.12 [1.01;1.24]	0.93 [0.71;1.21]
Intercept	3.83 [3.42;4.28]	0.93 [0.71;1.24]
last born	0.99 [0.97;1.00]	1.00 [0.95;1.05]
male	1.04 [1.02;1.05]	1.05 [1.01;1.08]
maternal loss 0-1	1.08 [0.99;1.18]	5.18 [4.22;6.43]
maternal loss 1-5	1.03 [0.98;1.07]	2.42 [2.17;2.72]

Effect	Hurdle Odds ratio	Zero-truncated Poisson Hazard ratio
maternal loss 10-15	0.95 [0.92;0.98]	2.04 [1.86;2.23]
maternal loss 15-20	0.96 [0.93;0.99]	1.79 [1.65;1.95]
maternal loss 20-25	0.93 [0.90;0.95]	1.50 [1.39;1.62]
maternal loss 25-30	0.96 [0.94;0.98]	1.33 [1.25;1.43]
maternal loss 30-35	0.97 [0.95;0.99]	1.27 [1.19;1.34]
maternal loss 35-40	0.99 [0.97;1.01]	1.16 [1.10;1.23]
maternal loss 40-45	0.98 [0.96;1.00]	1.09 [1.03;1.16]
maternal loss 5-10	0.98 [0.95;1.02]	2.12 [1.93;2.32]
maternalage factor 10-20	1.04 [0.99;1.09]	1.04 [0.90;1.21]
maternalage factor 35-59	1.07 [1.05;1.08]	1.07 [1.02;1.12]
nr siblings	1.03 [1.03;1.03]	1.03 [1.02;1.04]
older siblings 1	1.00 [0.98;1.02]	0.99 [0.94;1.05]
older siblings 2	1.00 [0.98;1.02]	1.03 [0.97;1.09]
older siblings 3	0.99 [0.97;1.01]	1.04 [0.98;1.11]
older siblings 4	0.96 [0.94;0.99]	1.03 [0.95;1.11]
older siblings 5+	0.95 [0.92;0.98]	0.98 [0.90;1.06]
paternal loss 0-1	1.09 [1.03;1.15]	2.12 [1.81;2.49]
paternal loss 1-5	1.02 [0.98;1.06]	1.81 [1.64;2.00]
paternal loss 10-15	0.97 [0.95;1.00]	1.62 [1.50;1.75]
paternal loss 15-20	0.93 [0.91;0.95]	1.47 [1.38;1.58]
paternal loss 20-25	0.97 [0.95;0.99]	1.35 [1.27;1.44]
paternal loss 25-30	0.98 [0.96;1.00]	1.24 [1.17;1.33]
paternal loss 30-35	0.98 [0.96;1.00]	1.18 [1.10;1.25]
paternal loss 35-40	1.02 [1.00;1.04]	1.13 [1.06;1.20]
paternal loss 40-45	1.02 [0.99;1.04]	1.05 [0.98;1.12]
paternal loss 5-10	0.98 [0.95;1.01]	1.87 [1.72;2.03]
paternalage	1.01 [1.00;1.01]	0.96 [0.94;0.99]

4.1.4 Paternal age effect

This is the effect of 10 years of paternal age within families on number of children, combined over the hurdle and Zero-truncated Poisson component, expressed as a change in percentage `((predicted value at t + 10y)/(predicted value at t)) - 1`.

effect	median_estimate	ci_95	ci_80
percentage change	2.37	[0.69;4.08]	[1.27;3.49]

4.1.4.1 Marginal effect plot

Paternal age effect on number of children The shaded areas show the 95% and 80% credibility intervals for the reference individuals and include uncertainty related to covariate effect sizes.

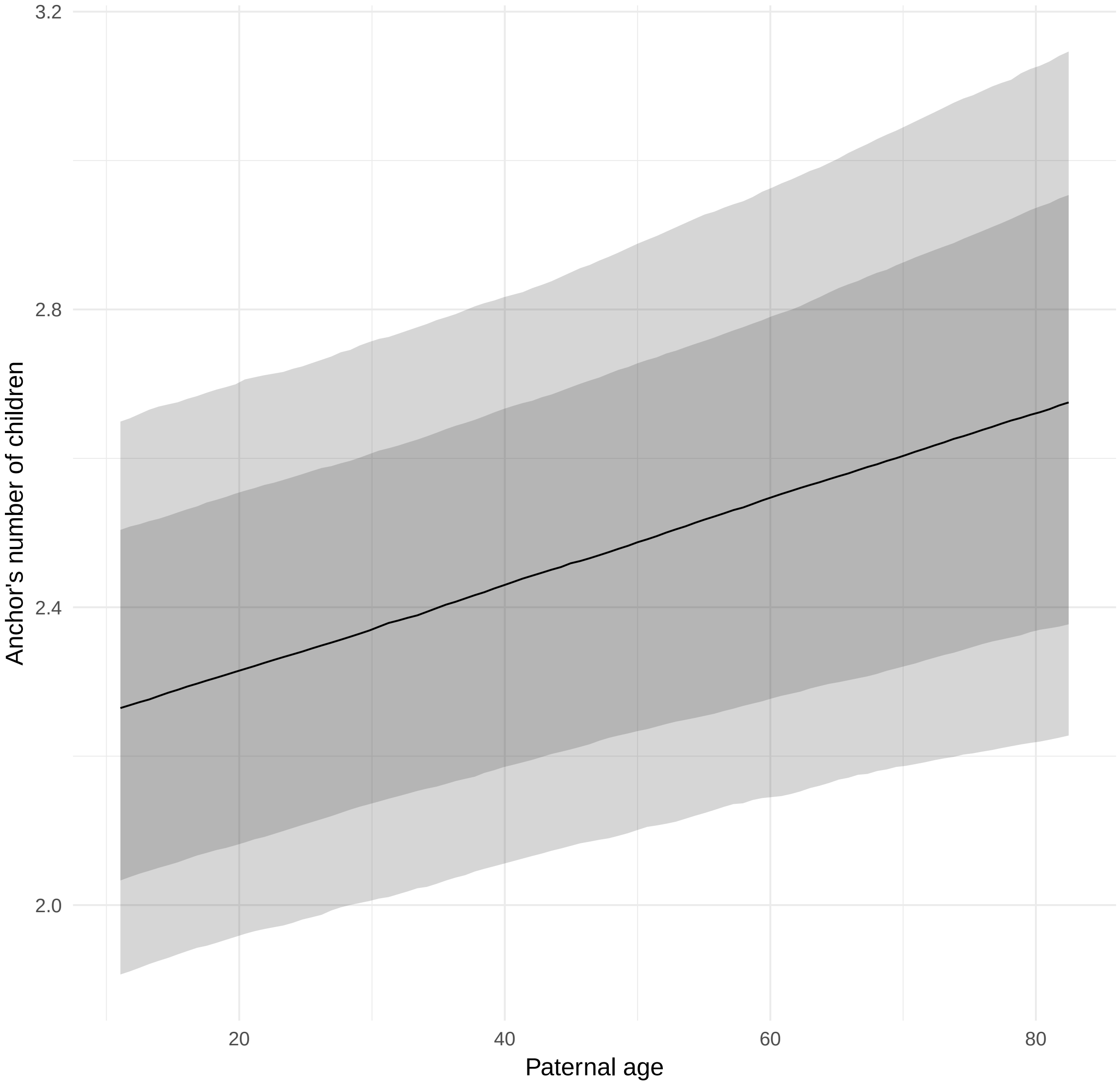

4.2 *m2*: Sibling comparison, no paternal age effect

Here, we compared siblings by including a random intercept for the family, but we modelled no effect for paternal age differences among siblings.

4.2.1 Model summary

Data: 56663 individuals nested in 14746 mother-father dyads.

Formula (Wilkinson notation): children ~ birth_cohort + male + maternalage.factor + paternalage.mean + paternal_loss + maternal_loss + older_siblings + nr.siblings + last_born + (1 | idParents).

- **family**: hurdle_poisson
- **link**: log

4.2.2 Priors

prior	class
normal(0,5)	b
student_t(3, 0, 5)	sd

4.2.3 Group-level effects

Component	Effect	Hurdle Estimate	Zero-truncated Poisson Estimate
idParents	sd(Intercept)	0.82 [0.79;0.85]	0.36 [0.34;0.37]

4.2.4 Population-level effects

Effect	Hurdle Odds ratio	Zero-truncated Poisson Hazard ratio
birth cohort 1750-1755	0.85 [0.67;1.07]	1.43 [0.80;2.57]
birth cohort 1755-1760	1.10 [0.91;1.32]	1.08 [0.64;1.80]
birth cohort 1760-1765	1.16 [0.98;1.38]	0.95 [0.60;1.51]
birth cohort 1765-1770	1.11 [0.93;1.31]	0.71 [0.44;1.13]
birth cohort 1770-1775	1.06 [0.89;1.26]	0.90 [0.56;1.46]
birth cohort 1775-1780	1.05 [0.88;1.23]	1.06 [0.67;1.68]
birth cohort 1780-1785	1.16 [0.97;1.37]	0.99 [0.62;1.58]
birth cohort 1785-1790	1.13 [0.96;1.33]	1.14 [0.74;1.73]
birth cohort 1790-1795	1.03 [0.88;1.21]	1.36 [0.89;2.06]
birth cohort 1795-1800	1.03 [0.87;1.19]	1.12 [0.74;1.67]
birth cohort 1800-1805	0.98 [0.83;1.13]	1.03 [0.68;1.54]
birth cohort 1805-1810	0.99 [0.84;1.15]	0.99 [0.66;1.47]
birth cohort 1810-1815	1.02 [0.87;1.18]	1.07 [0.71;1.57]
birth cohort 1815-1820	1.07 [0.92;1.24]	0.90 [0.60;1.34]
birth cohort 1820-1825	1.09 [0.93;1.27]	0.81 [0.55;1.19]
birth cohort 1825-1830	1.05 [0.90;1.21]	0.81 [0.55;1.21]
birth cohort 1830-1835	1.07 [0.92;1.24]	0.83 [0.56;1.23]
birth cohort 1835-1840	1.07 [0.91;1.24]	0.81 [0.54;1.20]
birth cohort 1840-1845	1.04 [0.90;1.21]	0.82 [0.55;1.22]
birth cohort 1845-1850	1.05 [0.90;1.22]	0.85 [0.57;1.27]

Effect	Hurdle Odds ratio	Zero-truncated Poisson Hazard ratio
Intercept	3.76 [3.21;4.44]	0.94 [0.62;1.44]
last born	0.98 [0.96;1.00]	1.01 [0.96;1.06]
male	1.04 [1.03;1.05]	1.05 [1.01;1.09]
maternal loss 0-1	1.06 [0.96;1.18]	6.41 [5.06;8.11]
maternal loss 1-5	1.00 [0.94;1.05]	2.80 [2.46;3.19]
maternal loss 10-15	0.96 [0.92;1.00]	2.24 [2.02;2.49]
maternal loss 15-20	0.96 [0.92;1.00]	1.96 [1.78;2.15]
maternal loss 20-25	0.93 [0.90;0.96]	1.58 [1.44;1.72]
maternal loss 25-30	0.97 [0.94;1.00]	1.37 [1.27;1.48]
maternal loss 30-35	0.98 [0.95;1.00]	1.28 [1.19;1.37]
maternal loss 35-40	1.00 [0.97;1.02]	1.16 [1.08;1.24]
maternal loss 40-45	0.98 [0.96;1.00]	1.08 [1.01;1.15]
maternal loss 5-10	0.97 [0.93;1.02]	2.38 [2.14;2.66]
maternalage factor 10-20	1.04 [0.99;1.11]	1.05 [0.89;1.26]
maternalage factor 35-59	1.05 [1.03;1.07]	1.08 [1.03;1.14]
nr siblings	1.03 [1.02;1.03]	1.04 [1.03;1.06]
older siblings 1	1.01 [0.99;1.02]	0.97 [0.92;1.02]
older siblings 2	1.01 [0.99;1.03]	0.99 [0.92;1.05]
older siblings 3	1.00 [0.98;1.03]	0.98 [0.91;1.06]
older siblings 4	0.97 [0.94;1.00]	0.96 [0.88;1.05]
older siblings 5+	0.97 [0.94;1.00]	0.91 [0.83;1.00]
paternal loss 0-1	1.05 [0.98;1.13]	2.39 [1.99;2.87]
paternal loss 1-5	1.02 [0.97;1.07]	1.97 [1.74;2.22]
paternal loss 10-15	0.97 [0.94;1.01]	1.69 [1.55;1.86]
paternal loss 15-20	0.92 [0.89;0.96]	1.53 [1.40;1.67]
paternal loss 20-25	0.97 [0.94;1.00]	1.38 [1.27;1.50]
paternal loss 25-30	0.97 [0.95;1.00]	1.26 [1.16;1.36]
paternal loss 30-35	0.98 [0.95;1.01]	1.17 [1.09;1.27]
paternal loss 35-40	1.02 [0.99;1.05]	1.13 [1.05;1.21]
paternal loss 40-45	1.03 [1.00;1.05]	1.04 [0.96;1.12]
paternal loss 5-10	0.97 [0.93;1.01]	2.00 [1.80;2.22]
paternalage mean	1.01 [0.99;1.02]	0.97 [0.94;1.01]

4.2.5 Paternal age effect

This is the effect of 10 years of paternal age within families on number of children, combined over the hurdle and Zero-truncated Poisson component, expressed as a change in percentage `((predicted value at t + 10y)/(predicted value at t)) - 1`.

This model did not contain a within family paternal age predictor.

4.3 *m3*: Sibling comparison, linear paternal age effect

Here, we compared siblings by including a random intercept for the family, and we modelled a linear effect for paternal age differences among siblings.

4.3.1 Model summary

Data: 56663 individuals nested in 14746 mother-father dyads.

Formula (Wilkinson notation): children ~ paternalage + birth_cohort + male + maternalage.factor + paternalage.mean + paternal_loss + maternal_loss + older_siblings + nr.siblings + last_born + (1 | idParents).

- **family**: hurdle_poisson
- **link**: log

4.3.2 Priors

prior	class
normal(0,5)	b
student_t(3, 0, 5)	sd

4.3.3 Group-level effects

Component	Effect	Hurdle Estimate	Zero-truncated Poisson Estimate
idParents	sd(Intercept)	0.82 [0.78;0.85]	0.36 [0.34;0.37]

4.3.4 Population-level effects

Effect	Hurdle Odds ratio	Zero-truncated Poisson Hazard ratio
birth cohort 1750-1755	0.84 [0.67;1.06]	1.45 [0.83;2.54]
birth cohort 1755-1760	1.10 [0.91;1.32]	1.09 [0.68;1.80]
birth cohort 1760-1765	1.16 [0.98;1.37]	0.96 [0.63;1.49]
birth cohort 1765-1770	1.10 [0.93;1.31]	0.72 [0.47;1.10]
birth cohort 1770-1775	1.06 [0.89;1.26]	0.91 [0.58;1.40]
birth cohort 1775-1780	1.05 [0.88;1.24]	1.07 [0.69;1.61]
birth cohort 1780-1785	1.16 [0.99;1.37]	1.00 [0.66;1.53]
birth cohort 1785-1790	1.13 [0.96;1.34]	1.15 [0.77;1.68]
birth cohort 1790-1795	1.03 [0.88;1.20]	1.39 [0.94;1.96]
birth cohort 1795-1800	1.02 [0.88;1.20]	1.14 [0.78;1.65]
birth cohort 1800-1805	0.97 [0.84;1.14]	1.05 [0.71;1.51]
birth cohort 1805-1810	0.99 [0.85;1.15]	1.00 [0.69;1.44]
birth cohort 1810-1815	1.01 [0.87;1.18]	1.08 [0.74;1.54]
birth cohort 1815-1820	1.07 [0.92;1.24]	0.91 [0.63;1.30]
birth cohort 1820-1825	1.09 [0.94;1.27]	0.82 [0.56;1.17]
birth cohort 1825-1830	1.04 [0.90;1.21]	0.82 [0.58;1.18]
birth cohort 1830-1835	1.07 [0.92;1.25]	0.84 [0.58;1.19]
birth cohort 1835-1840	1.07 [0.92;1.24]	0.82 [0.57;1.16]
birth cohort 1840-1845	1.05 [0.90;1.21]	0.83 [0.58;1.18]
birth cohort 1845-1850	1.05 [0.91;1.23]	0.86 [0.60;1.22]
Intercept	3.74 [3.19;4.39]	0.93 [0.65;1.38]
last born	0.98 [0.96;1.00]	1.01 [0.96;1.06]
male	1.04 [1.03;1.05]	1.05 [1.01;1.09]
maternal loss 0-1	1.07 [0.96;1.18]	6.40 [5.08;8.15]
maternal loss 1-5	1.00 [0.95;1.06]	2.78 [2.42;3.20]
maternal loss 10-15	0.96 [0.93;1.01]	2.23 [1.99;2.50]
maternal loss 15-20	0.96 [0.93;1.00]	1.96 [1.78;2.16]
maternal loss 20-25	0.93 [0.90;0.97]	1.58 [1.45;1.73]

Effect	Hurdle Odds ratio	Zero-truncated Poisson Hazard ratio
maternal loss 25-30	0.97 [0.94;1.00]	1.37 [1.26;1.49]
maternal loss 30-35	0.98 [0.95;1.01]	1.28 [1.19;1.38]
maternal loss 35-40	1.00 [0.98;1.03]	1.15 [1.08;1.23]
maternal loss 40-45	0.98 [0.96;1.00]	1.08 [1.01;1.16]
maternal loss 5-10	0.98 [0.94;1.03]	2.37 [2.11;2.67]
maternalage factor 10-20	1.04 [0.98;1.10]	1.06 [0.89;1.27]
maternalage factor 35-59	1.07 [1.04;1.09]	1.07 [1.01;1.14]
nr siblings	1.02 [1.02;1.03]	1.05 [1.03;1.06]
older siblings 1	1.02 [0.99;1.04]	0.96 [0.90;1.02]
older siblings 2	1.03 [1.00;1.06]	0.96 [0.89;1.05]
older siblings 3	1.04 [1.00;1.07]	0.95 [0.86;1.05]
older siblings 4	1.01 [0.97;1.06]	0.92 [0.81;1.04]
older siblings 5+	1.03 [0.97;1.09]	0.86 [0.73;1.01]
paternal loss 0-1	1.06 [0.99;1.14]	2.38 [1.98;2.85]
paternal loss 1-5	1.03 [0.98;1.08]	1.95 [1.73;2.21]
paternal loss 10-15	0.98 [0.94;1.02]	1.69 [1.53;1.86]
paternal loss 15-20	0.93 [0.90;0.96]	1.53 [1.39;1.67]
paternal loss 20-25	0.97 [0.94;1.00]	1.38 [1.27;1.50]
paternal loss 25-30	0.98 [0.95;1.01]	1.25 [1.15;1.35]
paternal loss 30-35	0.99 [0.96;1.01]	1.17 [1.09;1.26]
paternal loss 35-40	1.02 [1.00;1.05]	1.13 [1.05;1.21]
paternal loss 40-45	1.03 [1.00;1.05]	1.04 [0.97;1.12]
paternal loss 5-10	0.98 [0.94;1.03]	1.99 [1.79;2.21]
paternalage	0.95 [0.91;0.98]	1.05 [0.95;1.17]
paternalage mean	1.06 [1.02;1.10]	0.93 [0.83;1.03]

4.3.5 Paternal age effect

This is the effect of 10 years of paternal age within families on number of children, combined over the hurdle and Zero-truncated Poisson component, expressed as a change in percentage `((predicted value at t + 10y)/(predicted value at t)) - 1`.

effect	median_estimate	ci_95	ci_80
percentage change	-7.29	[-13.40; -1.07]	[-11.15; -3.33]

4.3.5.1 Marginal effect plot

Paternal age effect on number of children The shaded areas show the 95% and 80% credibility intervals for the reference individuals and include uncertainty related to covariate effect sizes.

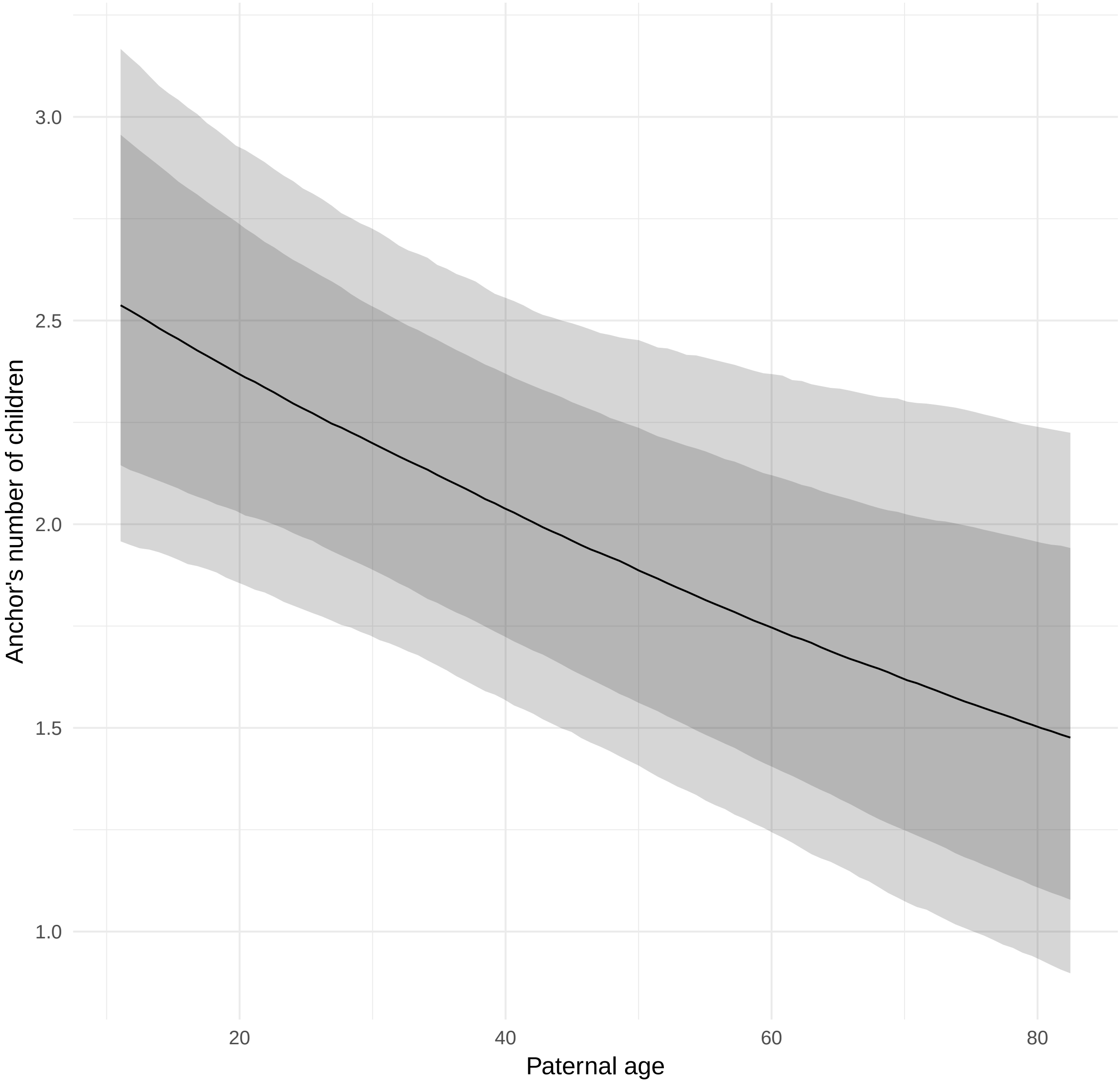

4.4 *m4*: Sibling comparison, nonlinear paternal age effect

Here, we compared siblings by including a random intercept for the family, and we modelled a possibly nonlinear effect for paternal age differences among siblings.

4.4.1 Model summary

Data: 56663 individuals nested in 14746 mother-father dyads.

Formula (Wilkinson notation): children ~ s(paternalage) + birth_cohort + male + maternalage.factor + paternalage.mean + paternal_loss + maternal_loss + older_siblings + nr.siblings + last_born + (1 | idParents).

- **family**: hurdle_poisson
- **link**: log

4.4.2 Priors

prior	class
normal(0,5)	b
student_t(3, 0, 5)	sd
student_t(3, 0, 10)	sds

4.4.3 Group-level effects

Component	Effect	Hurdle Estimate	Zero-truncated Poisson Estimate
idParents	sd(Intercept)	0.82 [0.79;0.85]	0.36 [0.35;0.37]

4.4.3.1 Splines

Effect	Hurdle Estimate	Zero-truncated Poisson Estimate
sds(spaternalage_1)	0.36 [0.01;1.30]	0.28 [0.03;0.75]

4.4.4 Population-level effects

Effect	Hurdle Odds ratio	Zero-truncated Poisson Hazard ratio
birth cohort 1750-1755	0.84 [0.68;1.06]	1.44 [0.86;2.50]
birth cohort 1755-1760	1.10 [0.91;1.33]	1.08 [0.68;1.73]
birth cohort 1760-1765	1.16 [0.98;1.39]	0.96 [0.63;1.46]
birth cohort 1765-1770	1.10 [0.93;1.33]	0.71 [0.46;1.09]
birth cohort 1770-1775	1.06 [0.90;1.28]	0.90 [0.58;1.39]
birth cohort 1775-1780	1.05 [0.89;1.25]	1.06 [0.70;1.56]
birth cohort 1780-1785	1.17 [0.99;1.38]	0.99 [0.66;1.50]
birth cohort 1785-1790	1.14 [0.97;1.34]	1.15 [0.78;1.68]
birth cohort 1790-1795	1.03 [0.88;1.21]	1.38 [0.95;1.99]
birth cohort 1795-1800	1.02 [0.87;1.20]	1.13 [0.79;1.59]
birth cohort 1800-1805	0.98 [0.84;1.15]	1.04 [0.73;1.49]

Effect	Hurdle Odds ratio	Zero-truncated Poisson Hazard ratio
birth cohort 1805-1810	0.99 [0.85;1.16]	1.00 [0.70;1.40]
birth cohort 1810-1815	1.02 [0.88;1.19]	1.07 [0.74;1.50]
birth cohort 1815-1820	1.07 [0.92;1.25]	0.91 [0.64;1.26]
birth cohort 1820-1825	1.09 [0.94;1.27]	0.82 [0.57;1.14]
birth cohort 1825-1830	1.05 [0.90;1.22]	0.82 [0.58;1.14]
birth cohort 1830-1835	1.07 [0.92;1.25]	0.83 [0.58;1.16]
birth cohort 1835-1840	1.07 [0.92;1.25]	0.81 [0.58;1.13]
birth cohort 1840-1845	1.05 [0.90;1.23]	0.83 [0.58;1.15]
birth cohort 1845-1850	1.06 [0.91;1.24]	0.86 [0.60;1.20]
Intercept	3.15 [2.54;3.85]	1.09 [0.64;1.90]
last born	0.98 [0.96;1.00]	1.01 [0.96;1.06]
male	1.04 [1.03;1.06]	1.05 [1.01;1.09]
maternal loss 0-1	1.07 [0.96;1.19]	6.37 [5.03;8.00]
maternal loss 1-5	1.00 [0.95;1.06]	2.78 [2.43;3.17]
maternal loss 10-15	0.97 [0.93;1.01]	2.24 [2.00;2.49]
maternal loss 15-20	0.97 [0.93;1.00]	1.96 [1.78;2.16]
maternal loss 20-25	0.93 [0.90;0.97]	1.58 [1.44;1.73]
maternal loss 25-30	0.97 [0.95;1.00]	1.37 [1.27;1.49]
maternal loss 30-35	0.98 [0.95;1.01]	1.28 [1.18;1.38]
maternal loss 35-40	1.00 [0.98;1.03]	1.15 [1.08;1.24]
maternal loss 40-45	0.98 [0.96;1.00]	1.08 [1.01;1.15]
maternal loss 5-10	0.98 [0.94;1.03]	2.38 [2.12;2.66]
maternalage factor 10-20	1.04 [0.98;1.10]	1.06 [0.89;1.27]
maternalage factor 35-59	1.06 [1.04;1.09]	1.07 [1.01;1.14]
nr siblings	1.02 [1.02;1.03]	1.05 [1.03;1.06]
older siblings 1	1.01 [0.99;1.04]	0.96 [0.91;1.03]
older siblings 2	1.02 [1.00;1.05]	0.97 [0.89;1.06]
older siblings 3	1.03 [0.99;1.07]	0.96 [0.86;1.07]
older siblings 4	1.00 [0.96;1.05]	0.93 [0.82;1.07]
older siblings 5+	1.02 [0.96;1.08]	0.88 [0.73;1.03]
paternal loss 0-1	1.06 [0.99;1.14]	2.38 [1.98;2.85]
paternal loss 1-5	1.03 [0.98;1.08]	1.95 [1.73;2.20]
paternal loss 10-15	0.98 [0.94;1.02]	1.68 [1.53;1.85]
paternal loss 15-20	0.93 [0.90;0.96]	1.53 [1.39;1.66]
paternal loss 20-25	0.97 [0.94;1.00]	1.38 [1.27;1.50]
paternal loss 25-30	0.98 [0.95;1.01]	1.26 [1.16;1.36]
paternal loss 30-35	0.98 [0.95;1.01]	1.17 [1.09;1.27]
paternal loss 35-40	1.02 [0.99;1.04]	1.13 [1.05;1.21]
paternal loss 40-45	1.03 [1.00;1.05]	1.04 [0.97;1.13]
paternal loss 5-10	0.98 [0.94;1.02]	1.99 [1.81;2.21]
paternalage mean	1.06 [1.01;1.10]	0.93 [0.83;1.04]
spaternalage	0.95 [0.87;1.03]	1.03 [0.88;1.23]

4.4.5 Paternal age effect

This is the effect of 10 years of paternal age within families on number of children, combined over the hurdle and Zero-truncated Poisson component, expressed as a change in percentage `((predicted value at t + 10y)/(predicted value at t)) - 1`.

effect	median_estimate	ci_95	ci_80
percentage change	-4.43	[-11.65; 2.93]	[-9.10; 0.47]

4.4.5.1 Marginal effect plot

Paternal age effect on number of children The shaded areas show the 95% and 80% credibility intervals for the reference individuals and include uncertainty related to covariate effect sizes.

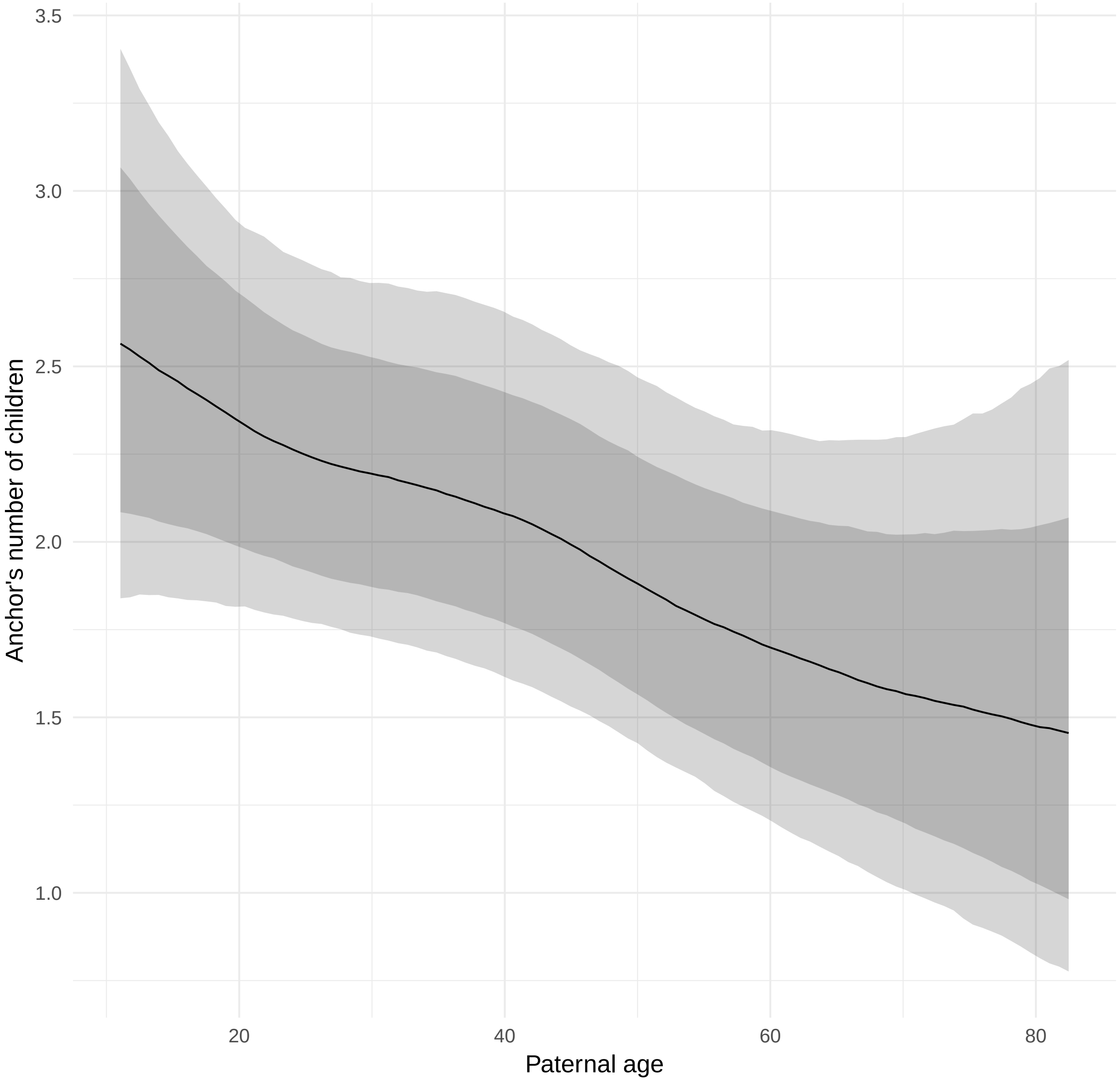

4.5 Main model comparison

We compare the four models using an approximate leave-one-out cross-validation information criterion as implemented in `brms` and `loo` and the Watanabe-Akaike information criterion.

4.5.1 Approximate leave-one-out (LOO) cross-validation

	LOOIC	SE
**m1**	191798	758.7
**m2**	185251	723
**m3**	185226	722.9
**m4**	185248	723
**m1 - m2**	6547	217.8
**m1 - m3**	6572	218.1
**m1 - m4**	6550	218.3
**m2 - m3**	25.39	15.36
**m2 - m4**	3.12	16.38
**m3 - m4**	-22.27	14.58

4.5.2 Watanabe-Akaike information criterion

	WAIC	SE
**m1**	191798	758.7
**m2**	184258	715.3
**m3**	184250	715.4
**m4**	184259	715.4
**m1 - m2**	7540	219.5
**m1 - m3**	7548	219.8
**m1 - m4**	7539	219.9
**m2 - m3**	7.65	11.75
**m2 - m4**	-1.11	12.89
**m3 - m4**	-8.76	10.85

4.6 *e1*: Selective episode: offspring survival of the first year

In the first selective episode model, we tested how much of the paternal age effect happens in the first selective episode, i.e. in the offspring's survival of the first year.

4.6.1 Model summary

Data: 56010 individuals nested in 14708 mother-father dyads.

Formula (Wilkinson notation): survive1y ~ paternalage + birth_cohort + male + maternalage.factor + paternalage.mean + paternal_loss + maternal_loss + older_siblings + nr.siblings + last_born + (1 | idParents).

- **family**: bernoulli
- **link**: cauchit

4.6.2 Priors

prior	class
normal(0,5)	b
student_t(3, 0, 5)	sd

4.6.3 Group-level effects

Component	Effect	Zero-truncated Poisson Estimate
idParents	sd(Intercept)	1.41 [1.33;1.49]

4.6.4 Population-level effects

Effect	Hurdle Odds ratio
birth cohort 1750-1755	0.83 [0.11; 12.83]
birth cohort 1755-1760	0.35 [0.08; 1.60]
birth cohort 1760-1765	0.25 [0.06; 0.87]
birth cohort 1765-1770	0.77 [0.17; 3.51]
birth cohort 1770-1775	0.67 [0.15; 3.03]
birth cohort 1775-1780	0.14 [0.04; 0.44]
birth cohort 1780-1785	0.14 [0.04; 0.42]
birth cohort 1785-1790	0.19 [0.05; 0.59]
birth cohort 1790-1795	0.16 [0.04; 0.48]
birth cohort 1795-1800	0.24 [0.06; 0.71]
birth cohort 1800-1805	0.23 [0.06; 0.69]
birth cohort 1805-1810	0.19 [0.05; 0.57]
birth cohort 1810-1815	0.17 [0.05; 0.52]
birth cohort 1815-1820	0.17 [0.05; 0.52]
birth cohort 1820-1825	0.27 [0.08; 0.79]
birth cohort 1825-1830	0.31 [0.08; 0.91]
birth cohort 1830-1835	0.26 [0.07; 0.80]
birth cohort 1835-1840	0.29 [0.08; 0.84]
birth cohort 1840-1845	0.40 [0.11; 1.21]

Effect	Hurdle Odds ratio
birth cohort 1845-1850	0.45 [0.13; 1.33]
Intercept	165.45 [54.03;604.23]
last born	0.93 [0.83; 1.04]
male	0.69 [0.63; 0.75]
maternal loss 0-1	0.03 [0.02; 0.04]
maternal loss 1-5	0.30 [0.24; 0.38]
maternal loss 10-15	0.58 [0.47; 0.71]
maternal loss 15-20	0.64 [0.52; 0.79]
maternal loss 20-25	0.65 [0.53; 0.78]
maternal loss 25-30	0.64 [0.53; 0.77]
maternal loss 30-35	0.73 [0.62; 0.87]
maternal loss 35-40	0.84 [0.72; 1.00]
maternal loss 40-45	0.93 [0.77; 1.10]
maternal loss 5-10	0.39 [0.32; 0.48]
maternalage factor 10-20	0.91 [0.63; 1.35]
maternalage factor 35-59	0.81 [0.71; 0.92]
nr siblings	0.78 [0.75; 0.80]
older siblings 1	1.83 [1.59; 2.10]
older siblings 2	2.50 [2.09; 2.96]
older siblings 3	2.86 [2.35; 3.52]
older siblings 4	4.02 [3.15; 5.19]
older siblings 5+	7.31 [5.28; 10.31]
paternal loss 0-1	0.37 [0.27; 0.51]
paternal loss 1-5	0.63 [0.50; 0.81]
paternal loss 10-15	0.74 [0.60; 0.91]
paternal loss 15-20	0.82 [0.67; 1.00]
paternal loss 20-25	0.92 [0.76; 1.12]
paternal loss 25-30	0.93 [0.77; 1.13]
paternal loss 30-35	0.87 [0.73; 1.05]
paternal loss 35-40	1.00 [0.84; 1.20]
paternal loss 40-45	1.10 [0.90; 1.34]
paternal loss 5-10	0.67 [0.54; 0.84]
paternalage	0.29 [0.24; 0.36]
paternalage mean	3.53 [2.82; 4.45]

4.6.5 Paternal age effect

This is the effect of 10 years of paternal age within families on probability of survival of the first year, combined over the hurdle and Zero-truncated Poisson component, expressed as a change in percentage `((predicted value at t + 10y)/(predicted value at t)) - 1`.

effect	median_estimate	ci_95	ci_80
percentage change	-1.82	[-3.14;-1.08]	[-2.63;-1.28]

4.6.5.1 Marginal effect plot

Paternal age effect on probability of survival of the first year The shaded areas show the 95% and 80% credibility intervals for the reference individuals and include uncertainty related to covariate effect sizes.

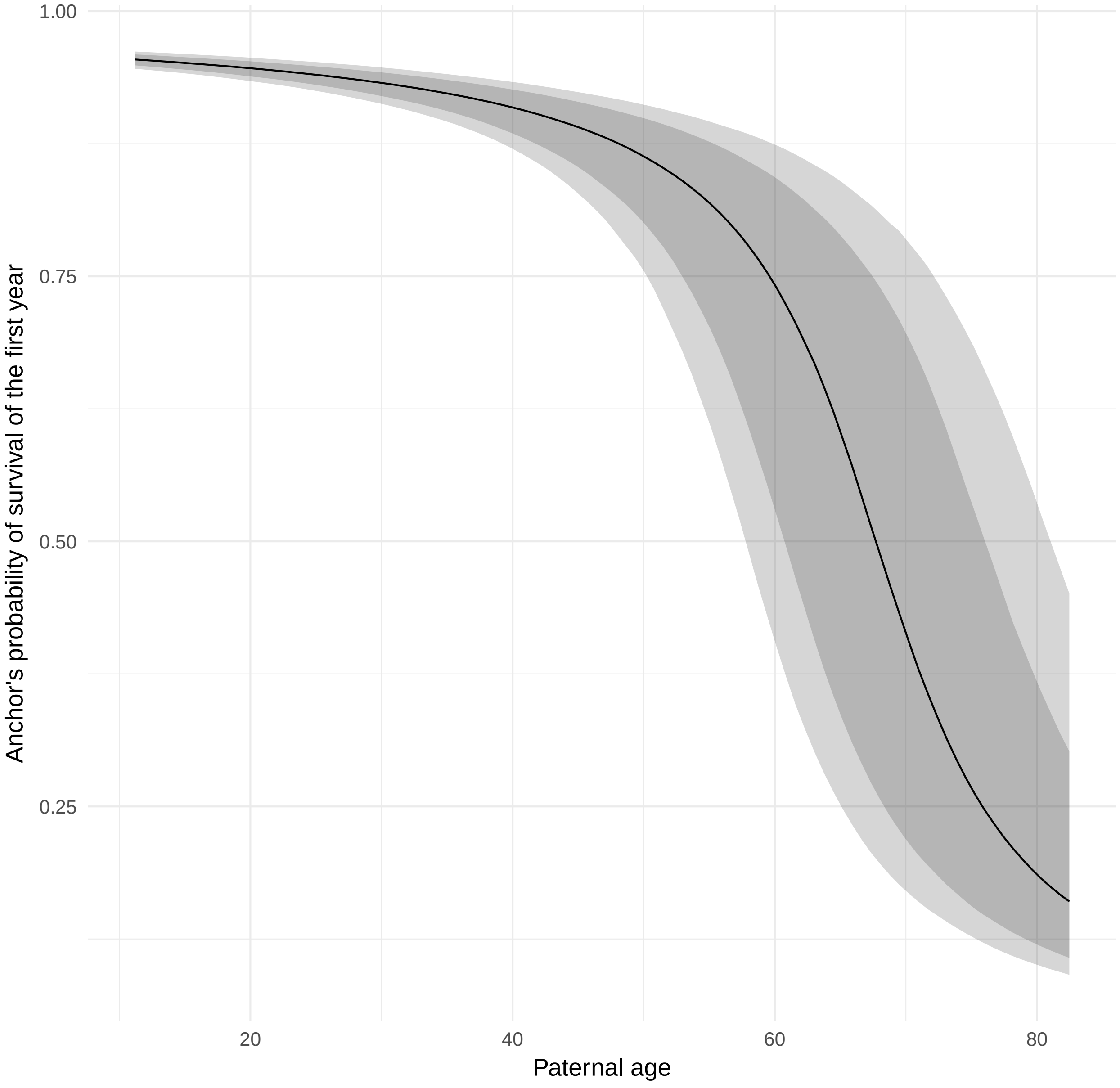

5 20th-century Sweden

5.1 *m1*: No sibling comparison

Here, we ignore the pedigree structure of the data to see whether it matters for the estimation of the paternal age effect.

5.1.1 Model summary

Data: 1408177 individuals.

Formula (Wilkinson notation): children ~ paternalage + birth_cohort + male + maternalage.factor + paternal_loss + maternal_loss + older_siblings + nr.siblings + last_born.

- **family**: poisson
- **link**: log

5.1.2 Priors

prior	class
normal(0,5)	b

5.1.3 Population-level effects

Effect	Hurdle Odds ratio
birth cohort 1950-1955	1.00 [1.00;1.00]
birth cohort 1955-1960	1.00 [1.00;1.01]
Intercept	2.08 [2.06;2.10]
last born	1.01 [1.01;1.01]
male	0.94 [0.94;0.94]
maternal loss 0-1	0.82 [0.71;0.94]
maternal loss 1-5	0.95 [0.89;1.00]
maternal loss 10-15	0.99 [0.98;1.01]
maternal loss 15-20	1.00 [0.98;1.01]
maternal loss 20-25	1.01 [1.00;1.02]
maternal loss 25-30	1.01 [1.00;1.01]
maternal loss 30-35	1.01 [1.00;1.01]
maternal loss 35-40	1.00 [0.99;1.00]
maternal loss 40-45	1.00 [0.99;1.01]
maternal loss 5-10	0.99 [0.96;1.02]
maternal loss unclear	0.98 [0.98;0.98]
maternalage factor 14-20	1.06 [1.05;1.06]
maternalage factor 3-56	1.00 [0.99;1.00]
nr siblings	1.04 [1.04;1.04]
older siblings 1	1.02 [1.01;1.02]
older siblings 2	1.02 [1.01;1.02]
older siblings 3	1.01 [1.00;1.02]
older siblings 4	0.99 [0.98;1.00]
older siblings 5+	0.94 [0.93;0.95]
paternal loss 0-1	1.12 [1.00;1.24]

Effect	Hurdle Odds ratio
paternal loss 1-5	1.03 [1.00;1.07]
paternal loss 10-15	1.00 [0.99;1.01]
paternal loss 15-20	1.00 [1.00;1.01]
paternal loss 20-25	1.00 [0.99;1.00]
paternal loss 25-30	1.00 [1.00;1.01]
paternal loss 30-35	1.00 [1.00;1.00]
paternal loss 35-40	1.00 [0.99;1.00]
paternal loss 40-45	0.99 [0.99;1.00]
paternal loss 5-10	0.98 [0.97;1.00]
paternal loss unclear	0.95 [0.94;0.95]
paternalage	0.95 [0.95;0.96]

5.1.4 Paternal age effect

This is the effect of 10 years of paternal age within families on number of children, combined over the hurdle and Zero-truncated Poisson component, expressed as a change in percentage `((predicted value at t + 10y)/(predicted value at t)) - 1`.

effect	median_estimate	ci_95	ci_80
percentage change	-4.60	[-4.83;-4.36]	[-4.75;-4.44]

5.1.4.1 Marginal effect plot

Paternal age effect on number of children The shaded areas show the 95% and 80% credibility intervals for the reference individuals and include uncertainty related to covariate effect sizes.

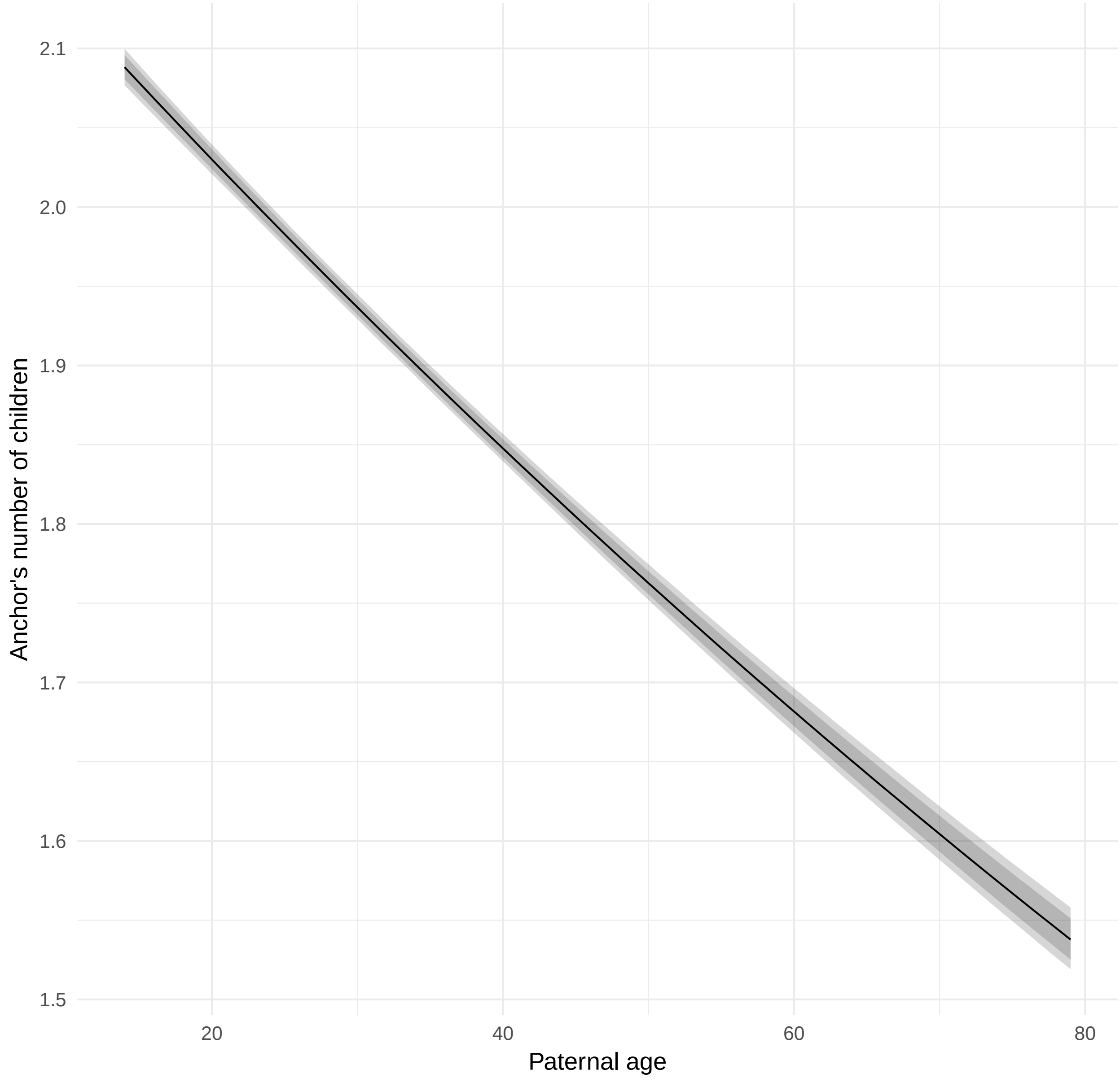

5.2 *m2*: Sibling comparison, no paternal age effect

Here, we compared siblings by including a random intercept for the family, but we modelled no effect for paternal age differences among siblings.

5.2.1 Model summary

Data: 1408177 individuals nested in 884975 mother-father dyads.

Formula (Wilkinson notation): children ~ birth_cohort + male + maternalage.factor + paternalage.mean + paternal_loss + maternal_loss + older_siblings + nr.siblings + last_born + (1 | idParents).

- **family**: poisson
- **link**: log

5.2.2 Priors

prior	class
normal(0,5)	b
student_t(3, 0, 5)	sd

5.2.3 Group-level effects

Component	Effect	Zero-truncated Poisson Estimate
idParents	sd(Intercept)	0.00 [0.00;0.01]

5.2.4 Population-level effects

Effect	Hurdle Odds ratio
birth cohort 1950-1955	1.00 [1.00;1.00]
birth cohort 1955-1960	1.00 [1.00;1.00]
Intercept	2.07 [2.05;2.09]
last born	1.01 [1.01;1.02]
male	0.94 [0.94;0.94]
maternal loss 0-1	0.82 [0.71;0.94]
maternal loss 1-5	0.94 [0.89;1.00]
maternal loss 10-15	0.99 [0.98;1.01]
maternal loss 15-20	1.00 [0.98;1.01]
maternal loss 20-25	1.01 [1.00;1.02]
maternal loss 25-30	1.01 [1.00;1.01]
maternal loss 30-35	1.01 [1.00;1.01]
maternal loss 35-40	1.00 [0.99;1.00]
maternal loss 40-45	1.00 [0.99;1.00]
maternal loss 5-10	0.99 [0.96;1.01]
maternal loss unclear	0.98 [0.98;0.98]
maternalage factor 14-20	1.06 [1.06;1.07]
maternalage factor 3-56	0.99 [0.99;0.99]
nr siblings	1.05 [1.04;1.05]
older siblings 1	1.00 [1.00;1.00]

Effect	Hurdle Odds ratio
older siblings 2	0.99 [0.98;0.99]
older siblings 3	0.97 [0.96;0.97]
older siblings 4	0.94 [0.93;0.95]
older siblings 5+	0.87 [0.86;0.88]
paternal loss 0-1	1.12 [1.02;1.24]
paternal loss 1-5	1.03 [0.99;1.07]
paternal loss 10-15	0.99 [0.98;1.00]
paternal loss 15-20	1.00 [0.99;1.01]
paternal loss 20-25	1.00 [0.99;1.00]
paternal loss 25-30	1.00 [1.00;1.01]
paternal loss 30-35	1.00 [0.99;1.00]
paternal loss 35-40	0.99 [0.99;1.00]
paternal loss 40-45	0.99 [0.99;1.00]
paternal loss 5-10	0.98 [0.96;1.00]
paternal loss unclear	0.95 [0.94;0.95]
paternalage mean	0.96 [0.95;0.96]

5.2.5 Paternal age effect

This is the effect of 10 years of paternal age within families on number of children, combined over the hurdle and Zero-truncated Poisson component, expressed as a change in percentage `((predicted value at t + 10y)/(predicted value at t)) - 1`.

This model did not contain a within family paternal age predictor.

5.3 *m3*: Sibling comparison, linear paternal age effect

Here, we compared siblings by including a random intercept for the family, and we modelled a linear effect for paternal age differences among siblings.

5.3.1 Model summary

Data: 1408177 individuals nested in 884975 mother-father dyads.

Formula (Wilkinson notation): children ~ paternalage + birth_cohort + male + maternalage.factor + paternalage.mean + paternal_loss + maternal_loss + older_siblings + nr.siblings + last_born + (1 | idParents).

- **family**: poisson
- **link**: log

5.3.2 Priors

prior	class
normal(0,5)	b
student_t(3, 0, 5)	sd

5.3.3 Group-level effects

Component	Effect	Zero-truncated Poisson Estimate
idParents	sd(Intercept)	0.00 [0.00;0.01]

5.3.4 Population-level effects

Effect	Hurdle Odds ratio
birth cohort 1950-1955	1.00 [1.00;1.00]
birth cohort 1955-1960	1.00 [1.00;1.01]
Intercept	2.08 [2.06;2.10]
last born	1.01 [1.01;1.01]
male	0.94 [0.94;0.94]
maternal loss 0-1	0.82 [0.71;0.95]
maternal loss 1-5	0.95 [0.89;1.00]
maternal loss 10-15	0.99 [0.98;1.01]
maternal loss 15-20	1.00 [0.98;1.01]
maternal loss 20-25	1.01 [1.00;1.02]
maternal loss 25-30	1.01 [1.00;1.01]
maternal loss 30-35	1.01 [1.00;1.01]
maternal loss 35-40	1.00 [0.99;1.00]
maternal loss 40-45	1.00 [0.99;1.00]
maternal loss 5-10	0.99 [0.96;1.02]
maternal loss unclear	0.98 [0.98;0.98]
maternalage factor 14-20	1.06 [1.05;1.06]
maternalage factor 3-56	1.00 [1.00;1.00]
nr siblings	1.04 [1.04;1.04]
older siblings 1	1.02 [1.01;1.02]
older siblings 2	1.02 [1.02;1.03]
older siblings 3	1.02 [1.01;1.03]
older siblings 4	1.00 [0.99;1.02]
older siblings 5+	0.95 [0.93;0.97]
paternal loss 0-1	1.12 [1.00;1.25]
paternal loss 1-5	1.03 [1.00;1.07]
paternal loss 10-15	1.00 [0.99;1.01]
paternal loss 15-20	1.00 [1.00;1.01]
paternal loss 20-25	1.00 [0.99;1.00]
paternal loss 25-30	1.00 [1.00;1.01]
paternal loss 30-35	1.00 [1.00;1.01]
paternal loss 35-40	1.00 [0.99;1.00]
paternal loss 40-45	0.99 [0.99;1.00]
paternal loss 5-10	0.98 [0.97;1.00]
paternal loss unclear	0.95 [0.94;0.95]
paternalage	0.95 [0.94;0.96]
paternalage mean	1.01 [1.00;1.01]

5.3.5 Paternal age effect

This is the effect of 10 years of paternal age within families on number of children, combined over the hurdle and Zero-truncated Poisson component, expressed as a change in percentage `((predicted value at t + 10y)/(predicted value at t)) - 1`.

effect	median_estimate	ci_95	ci_80
percentage change	-5.15	[-5.94;-4.36]	[-5.67;-4.63]

5.3.5.1 Marginal effect plot

Paternal age effect on number of children The shaded areas show the 95% and 80% credibility intervals for the reference individuals and include uncertainty related to covariate effect sizes.

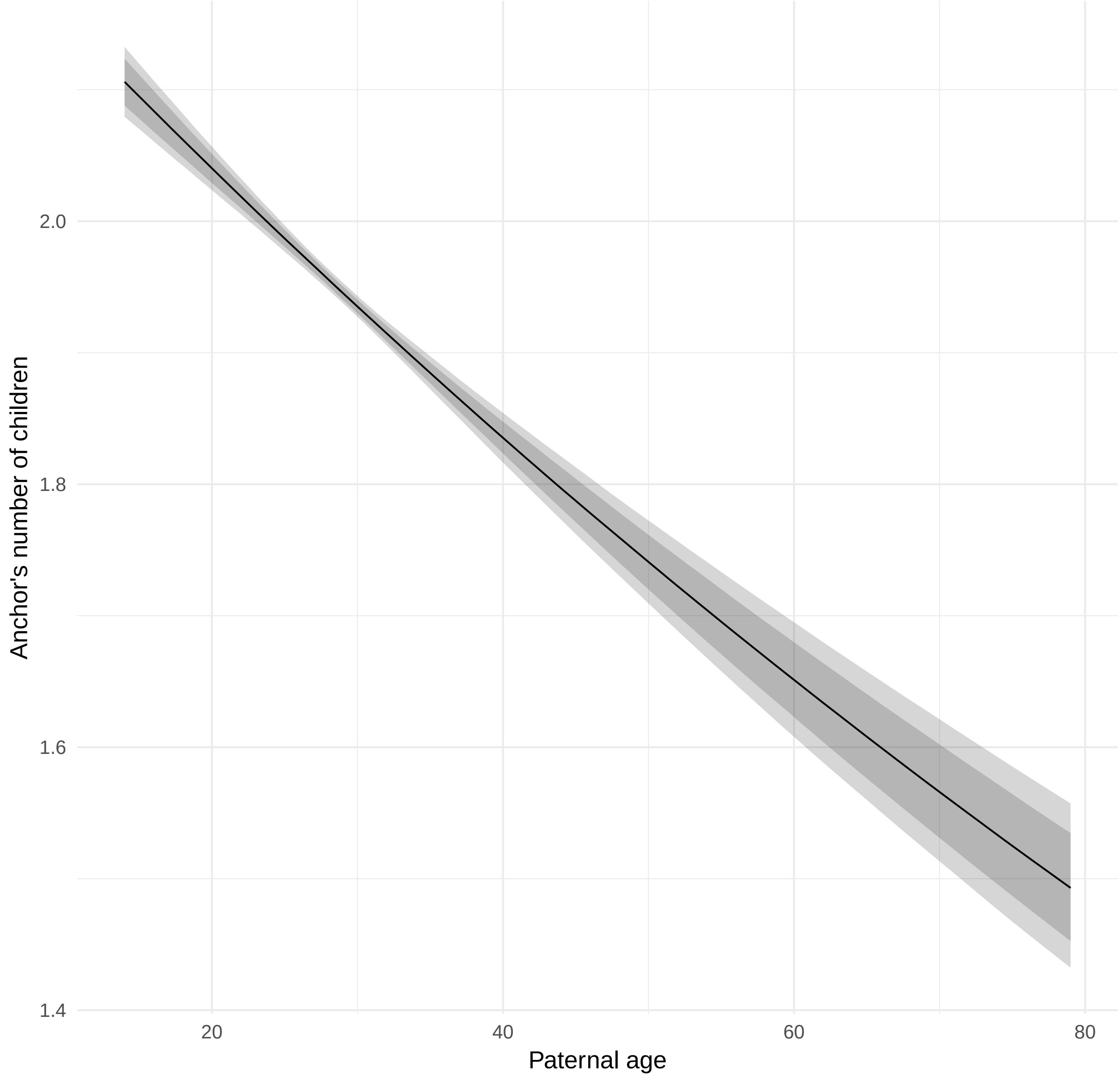

5.4 *m4*: Sibling comparison, nonlinear paternal age effect

Here, we compared siblings by including a random intercept for the family, and we modelled a possibly nonlinear effect for paternal age differences among siblings.

5.4.1 Model summary

Data: 1408177 individuals nested in 884975 mother-father dyads.

Formula (Wilkinson notation): children ~ s(paternalage) + birth_cohort + male + maternalage.factor + paternalage.mean + paternal_loss + maternal_loss + older_siblings + nr.siblings + last_born + (1 | idParents).

- **family**: poisson
- **link**: log

5.4.2 Priors

prior	class
normal(0,5)	b
student_t(3, 0, 5)	sd
student_t(3, 0, 10)	sds

5.4.3 Group-level effects

Component	Effect	Zero-truncated Poisson Estimate
idParents	sd(Intercept)	0.00 [0.00;0.01]

5.4.3.1 Splines

Effect	Zero-truncated Poisson Estimate
sds(spaternalage_1)	0.13 [0.06;0.37]

5.4.4 Population-level effects

Effect	Hurdle Odds ratio
birth cohort 1950-1955	1.00 [1.00;1.00]
birth cohort 1955-1960	1.00 [1.00;1.01]
Intercept	1.75 [1.71;1.80]
last born	1.01 [1.01;1.01]
male	0.94 [0.94;0.94]
maternal loss 0-1	0.82 [0.71;0.94]
maternal loss 1-5	0.94 [0.89;1.00]
maternal loss 10-15	0.99 [0.98;1.01]
maternal loss 15-20	0.99 [0.98;1.01]
maternal loss 20-25	1.01 [1.00;1.02]
maternal loss 25-30	1.00 [1.00;1.01]

Effect	Hurdle Odds ratio
maternal loss 30-35	1.00 [1.00;1.01]
maternal loss 35-40	1.00 [0.99;1.00]
maternal loss 40-45	1.00 [0.99;1.00]
maternal loss 5-10	0.99 [0.96;1.02]
maternal loss unclear	0.98 [0.98;0.98]
maternalage factor 14-20	1.05 [1.04;1.05]
maternalage factor 3-56	0.99 [0.99;1.00]
nr siblings	1.04 [1.04;1.04]
older siblings 1	1.02 [1.02;1.03]
older siblings 2	1.03 [1.02;1.04]
older siblings 3	1.02 [1.01;1.03]
older siblings 4	1.01 [0.99;1.02]
older siblings 5+	0.95 [0.94;0.97]
paternal loss 0-1	1.11 [1.00;1.22]
paternal loss 1-5	1.03 [0.99;1.06]
paternal loss 10-15	0.99 [0.98;1.00]
paternal loss 15-20	1.00 [0.99;1.01]
paternal loss 20-25	0.99 [0.99;1.00]
paternal loss 25-30	1.00 [0.99;1.01]
paternal loss 30-35	1.00 [0.99;1.00]
paternal loss 35-40	1.00 [0.99;1.00]
paternal loss 40-45	0.99 [0.99;1.00]
paternal loss 5-10	0.98 [0.96;0.99]
paternal loss unclear	0.94 [0.94;0.95]
paternalage mean	1.01 [1.00;1.02]
spaternalage	0.96 [0.92;1.00]

5.4.5 Paternal age effect

This is the effect of 10 years of paternal age within families on number of children, combined over the hurdle and Zero-truncated Poisson component, expressed as a change in percentage `((predicted value at t + 10y)/(predicted value at t)) - 1`.

effect	median_estimate	ci_95	ci_80
percentage change	-6.88	[-7.62;-6.03]	[-7.40;-6.32]

5.4.5.1 Marginal effect plot

Paternal age effect on number of children The shaded areas show the 95% and 80% credibility intervals for the reference individuals and include uncertainty related to covariate effect sizes.

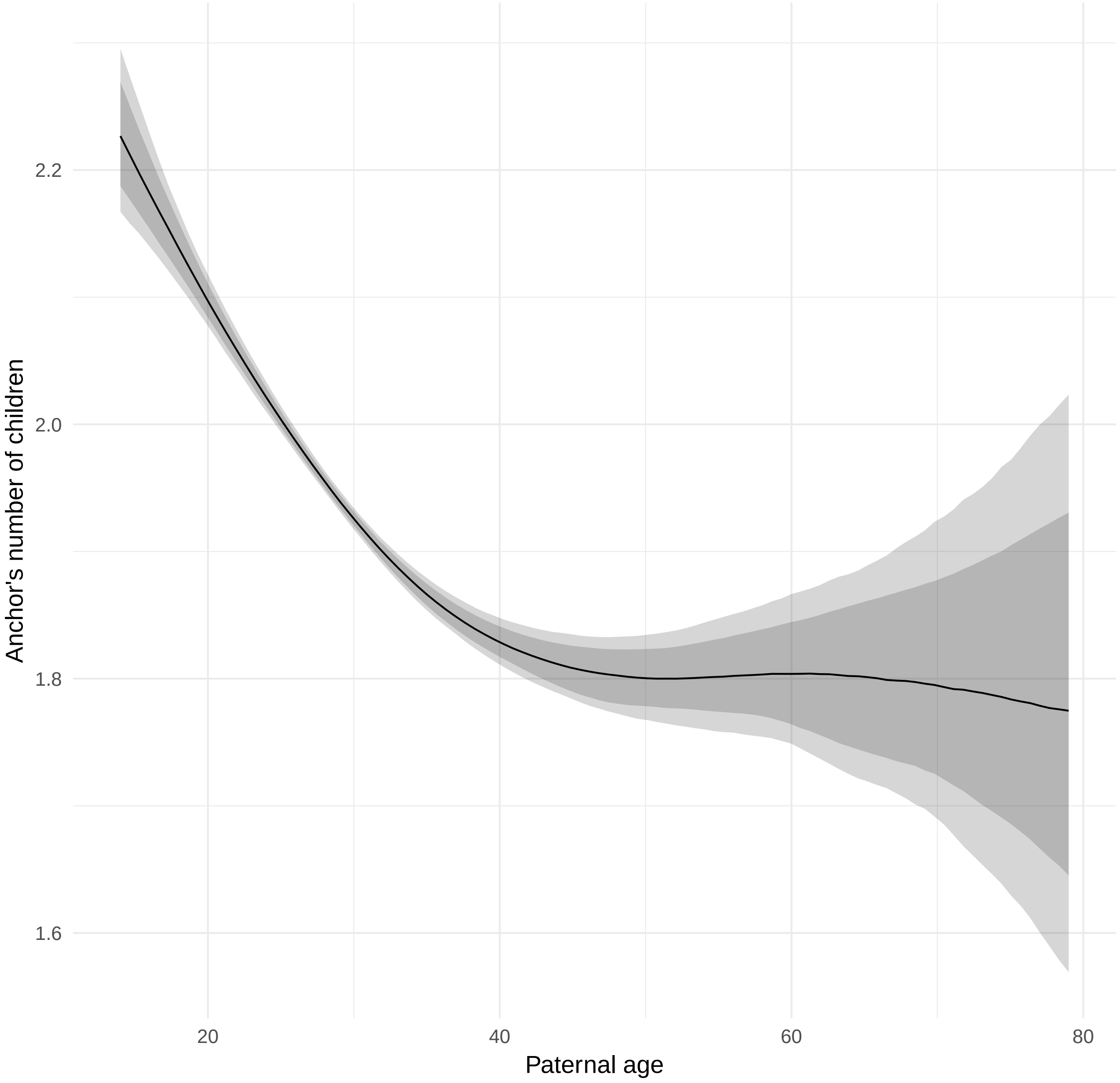

5.5 Main model comparison

Because of computational limitations, we could not compute LOO and WAIC for the complete dataset models in this population. Instead, we computed the main models and their LOOs and WAICs using a randomly drawn subset of 100,000 families (the same that was used for the robustness analyses).

We compare the four models using an approximate leave-one-out cross-validation information criterion as implemented in `brms` and `loo` and the Watanabe-Akaike information criterion.

5.5.1 Approximate leave-one-out (LOO) cross-validation

	LOOIC	SE
**m1**	415187	388.2
**m2**	415196	388
**m3**	415186	388.1
**m4**	415165	387.9
**m1 - m2**	-9.13	6.31
**m1 - m3**	0.93	0.42
**m1 - m4**	22.09	9.37
**m2 - m3**	10.07	6.47
**m2 - m4**	31.22	11.3
**m3 - m4**	21.15	9.36

5.5.2 Watanabe-Akaike information criterion

	WAIC	SE
**m1**	415187	388.2
**m2**	415196	388
**m3**	415186	388.1
**m4**	415165	387.9
**m1 - m2**	-9.1	6.31
**m1 - m3**	0.89	0.42
**m1 - m4**	22.04	9.37
**m2 - m3**	9.98	6.47
**m2 - m4**	31.14	11.3
**m3 - m4**	21.16	9.36

5.6 *e1*: Selective episode: offspring survival of the first year

In the first selective episode model, we tested how much of the paternal age effect happens in the first selective episode, i.e. in the offspring's survival of the first year.

5.6.1 Model summary

Data: 363744 individuals nested in 200000 mother-father dyads.

Formula (Wilkinson notation): survive1y ~ paternalage + birth_cohort + male + maternalage.factor + paternalage.mean + paternal_loss + maternal_loss + older_siblings + nr.siblings + last_born + (1 | idParents).

- **family**: bernoulli
- **link**: cauchit

5.6.2 Priors

prior	class
normal(0,5)	b
student_t(3, 0, 5)	sd

5.6.3 Group-level effects

Component	Effect	Zero-truncated Poisson Estimate
idParents	sd(Intercept)	21.41 [19.76;22.96]

5.6.4 Population-level effects

Effect	Hurdle Odds ratio
birth cohort 1970-1975	0.0e+00 [0.0e+00;1.7e-01]
birth cohort 1975-1980	4.0e-02 [0.0e+00;2.1e+00]
birth cohort 1980-1985	1.1e+02 [1.4e+00;7.2e+03]
birth cohort 1985-1990	5.0e+01 [6.6e-01;3.7e+03]
birth cohort 1990-1995	3.9e+02 [5.6e+00;3.3e+04]
birth cohort 1995-2000	2.6e+06 [7.5e+03;1.0e+09]
Intercept	5.2e+41 [7.2e+37;7.2e+45]
last born	0.0e+00 [0.0e+00;0.0e+00]
male	1.0e-02 [0.0e+00;6.0e-02]
maternal loss 0-1	1.0e-02 [0.0e+00;2.6e+02]
maternal loss 1-5	6.7e+00 [0.0e+00;7.3e+04]
maternal loss 10-15	7.0e-01 [0.0e+00;3.6e+03]
maternal loss 15-20	0.0e+00 [0.0e+00;1.7e+00]
maternal loss 20-25	0.0e+00 [0.0e+00;1.4e+00]
maternal loss 25-30	1.0e-02 [0.0e+00;6.9e+00]
maternal loss 30-35	5.0e-02 [0.0e+00;5.4e+01]
maternal loss 35-40	2.0e-01 [0.0e+00;7.1e+02]
maternal loss 40-45	2.6e+00 [0.0e+00;2.5e+04]
maternal loss 5-10	4.7e+00 [0.0e+00;3.8e+04]

Effect	Hurdle Odds ratio
maternalage factor 14-20	1.5e+00 [3.0e-02;1.4e+02]
maternalage factor 3-56	1.0e-02 [0.0e+00;2.3e-01]
nr siblings	0.0e+00 [0.0e+00;0.0e+00]
older siblings 1	0.0e+00 [0.0e+00;2.0e-02]
older siblings 2	1.0e+00 [2.0e-02;5.3e+01]
older siblings 3	2.0e+06 [1.7e+04;2.8e+08]
older siblings 4	1.5e+07 [6.0e+04;3.0e+09]
older siblings 5+	1.3e+11 [1.2e+08;1.5e+14]
paternal loss 0-1	1.4e+00 [0.0e+00;2.2e+04]
paternal loss 1-5	0.0e+00 [0.0e+00;5.7e+01]
paternal loss 10-15	3.5e+01 [3.0e-02;7.1e+04]
paternal loss 15-20	3.0e-02 [0.0e+00;1.1e+01]
paternal loss 20-25	7.0e-02 [0.0e+00;3.9e+01]
paternal loss 25-30	2.4e+00 [2.0e-02;5.9e+02]
paternal loss 30-35	7.5e+00 [4.0e-02;2.9e+03]
paternal loss 35-40	1.0e-02 [0.0e+00;2.7e+00]
paternal loss 40-45	2.7e-01 [0.0e+00;7.8e+03]
paternal loss 5-10	5.2e+01 [2.0e-02;3.5e+05]
paternalage	0.0e+00 [0.0e+00;0.0e+00]
paternalage mean	2.8e+04 [4.9e+02;1.4e+06]

5.6.5 Paternal age effect

This is the effect of 10 years of paternal age within families on probability of survival of the first year, combined over the hurdle and Zero-truncated Poisson component, expressed as a change in percentage `((predicted value at t + 10y)/(predicted value at t)) - 1`.

effect	median_estimate	ci_95	ci_80
percentage change	-0.05	[-0.06;-0.03]	[-0.06;-0.03]

5.6.5.1 Marginal effect plot

Paternal age effect on probability of survival of the first year The shaded areas show the 95% and 80% credibility intervals for the reference individuals and include uncertainty related to covariate effect sizes.

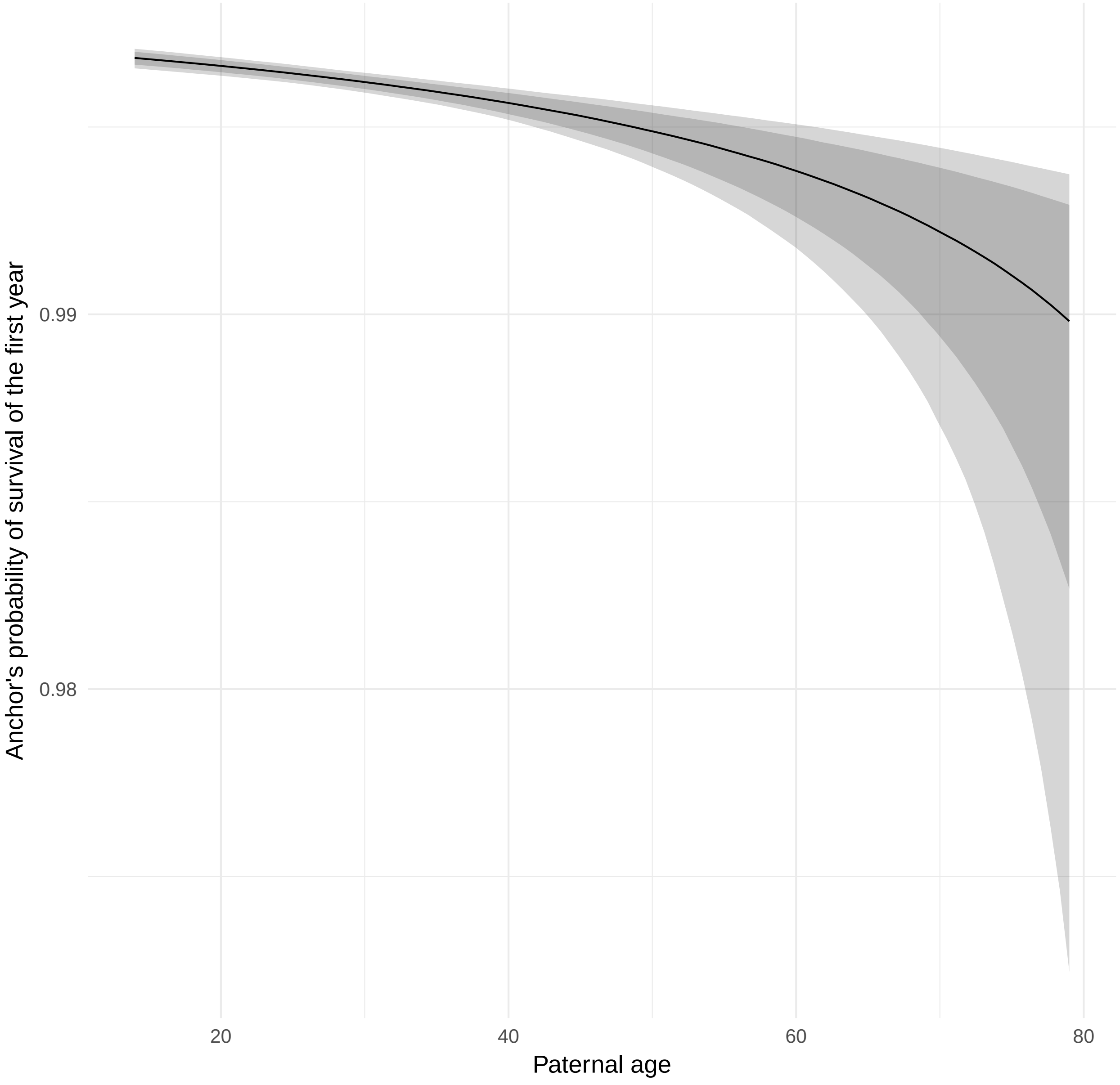

6 Robustness analyses documentation

All of the following models are the same as our main model m3, except for the noted changes to test robustness.

6.1 Table

Estimates of the effect of 10 years paternal age on number of children within families (comparing siblings) are given in percentage change. 95% credibility interval are given in brackets. Click the model name to be taken to further details on the supplementary website.

Population	Model	Estimate
20th-century Sweden	m3 model with 95% CI	-5.15 [-5.94;-4.36]
Historical Sweden	m3 model with 95% CI	-7.29 [-13.40;-1.07]
Krummhörn	m3 model with 95% CI	-8.41 [-24.83;12.03]
Québec	m3 model with 95% CI	-3.00 [-6.08;0.24]
20th-century Sweden	r1 relaxed exclusion criteria	-4.96 [-7.67;-2.18]
Historical Sweden	r1 relaxed exclusion criteria	-17.32 [-22.85;-11.80]
Krummhörn	r1 relaxed exclusion criteria	-7.11 [-23.86; 6.73]
Québec	r1 relaxed exclusion criteria	-1.67 [-4.39;0.62]
20th-century Sweden	r2 few controls	-3.20 [-4.37;-2.00]
Historical Sweden	r2 few controls	-20.42 [-23.68;-17.37]
Krummhörn	r2 few controls	-16.37 [-22.17;-10.38]
Québec	r2 few controls	-6.62 [-7.96;-5.24]
20th-century Sweden	r3 birth order continuous	-2.08 [-2.87;-1.29]
Historical Sweden	r3 birth order continuous	-6.02 [-12.94; 1.64]
Krummhörn	r3 birth order continuous	-23.54 [-37.92;-5.40]
Québec	r3 birth order continuous	-1.68 [-6.18;3.55]
20th-century Sweden	r4 control dependent sibs	-3.97 [-5.37;-2.56]
Historical Sweden	r4 control dependent sibs	-2.58 [-6.01;1.07]
Krummhörn	r4 control dependent sibs	0.68 [-8.48;10.93]
Québec	r4 control dependent sibs	0.53 [-1.33;2.46]
20th-century Sweden	r5 birth order interact siblings	-4.52 [-7.05;-1.85]
Historical Sweden	r5 birth order interact siblings	-6.80 [-12.77;-0.16]
Krummhörn	r5 birth order interact siblings	-10.72 [-28.05; 9.92]
Québec	r5 birth order interact siblings	-0.93 [-4.21;2.60]
20th-century Sweden	r6 no birth order control	-3.70 [-5.13;-2.29]
Historical Sweden	r6 no birth order control	-2.34 [-5.7802;1.3217]
Krummhörn	r6 no birth order control	1.05 [-8.48;11.09]
Québec	r6 no birth order control	0.28 [-1.73;2.28]
20th-century Sweden	r7 less parental loss control	-4.84 [-7.21;-2.19]
Historical Sweden	r7 less parental loss control	-24.13 [-30.14;-18.17]
Krummhörn	r7 less parental loss control	-18.19 [-34.37;-0.24]
Québec	r7 less parental loss control	-7.42 [-10.47;-4.22]
20th-century Sweden	r8 adjust for first born adult	-4.80 [-7.20;-2.42]
Historical Sweden	r8 adjust for first born adult	-11.03 [-16.11;-5.49]
Krummhörn	r8 adjust for first born adult	-7.93 [-21.26; 8.17]
Québec	r8 adjust for first born adult	-8.63 [-12.34;-4.75]
20th-century Sweden	r9 continuous byear adjustment	-4.60 [-7.24;-1.98]
Historical Sweden	r9 continuous byear adjustment	-7.04 [-12.40;-1.31]
Krummhörn	r9 continuous byear adjustment	-6.35 [-21.47;11.08]
Québec	r9 continuous byear adjustment	-3.86 [-7.35;-0.26]
20th-century Sweden	r10 add random slope	-4.57 [-7.15;-2.11]

Population	Model	Estimate
Historical Sweden	r10 add random slope	-23.70 [-29.41;-17.92]
Krummhörn	r10 add random slope	-8.56 [-25.31;11.57]
Québec	r10 add random slope	-5.17 [-7.98;-1.30]
20th-century Sweden	r11 separate random effects for parents	-4.61 [-7.14;-1.90]
Historical Sweden	r11 separate random effects for parents	-7.30 [-13.43;-0.80]
Krummhörn	r11 separate random effects for parents	-8.41 [-25.14;11.23]
Québec	r11 separate random effects for parents	-2.68 [-5.95;0.52]
20th-century Sweden	r12 sex moderation	-3.94 [-6.61;-1.18]
Historical Sweden	r12 sex moderation	-7.88 [-13.91;-1.34]
Krummhörn	r12 sex moderation	-9.05 [-25.49;11.82]
Québec	r12 sex moderation	-3.24 [-6.43;0.31]
20th-century Sweden	r13 control paternal afb	-4.15 [-6.65;-1.65]
Historical Sweden	r13 control paternal afb	-8.34 [-14.90;-2.41]
Krummhörn	r13 control paternal afb	-8.15 [-24.89;12.04]
Québec	r13 control paternal afb	-2.19 [-5.22986;1.19428]
20th-century Sweden	r14 compare lfe	
Historical Sweden	r14 compare lfe	
Krummhörn	r14 compare lfe	
Québec	r14 compare lfe	
Historical Sweden	r15 region moderator parish ranef	-6.89 [-12.58;-1.25]
Krummhörn	r15 region moderator parish ranef	-8.31 [-24.61;10.99]
Québec	r15 region moderator parish ranef	-2.17 [-5.16;0.79]
Historical Sweden	r16 restrict to skelleftea	-10.22 [-22.12; 4.20]
20th-century Sweden	r17 simulate downs	-4.09 [-6.66;-1.24]
Historical Sweden	r17 simulate downs	0.41 [-6.23;7.48]
Krummhörn	r17 simulate downs	2.67 [-15.43;25.56]
Québec	r17 simulate downs	0.96 [-2.48;4.45]
20th-century Sweden	r18 hurdle poisson	-3.14 [-5.48;-0.71]
Historical Sweden	r18 hurdle poisson	-8.54 [-12.54;-4.62]
Krummhörn	r18 control paternal afb	-7.10 [-24.21;13.41]
Krummhörn	r18 hurdle poisson	-2.09 [-13.04;10.40]
Québec	r18 hurdle poisson	-2.61 [-5.0581;0.0065]
20th-century Sweden	r19 normal distribution	-3.73 [-6.14;-1.40]
Historical Sweden	r19 normal distribution	-6.48 [-12.63;-0.85]
Krummhörn	r19 normal distribution	-7.69 [-21.51; 8.34]
Québec	r19 normal distribution	-4.32 [-7.39;-1.11]
20th-century Sweden	r20 no maternalage control	-5.28 [-7.65;-2.98]
Historical Sweden	r20 no maternalage control	-5.55 [-11.28; 0.50]
Krummhörn	r20 no maternalage control	-13.61 [-29.41; 3.73]
Québec	r20 no maternalage control	-3.49 [-6.27;-0.66]
20th-century Sweden	r21 continuous maternalage	-0.94 [-1.74;-0.13]
Historical Sweden	r21 continuous maternalage	-8.94 [-15.31;-2.32]
Krummhörn	r21 continuous maternalage	-10.86 [-28.22; 8.72]
Québec	r21 continuous maternalage	-1.75 [-5.37;1.84]
Historical Sweden	r22 relaxed exclusion censoring	-25.50 [-29.91;-21.11]
Krummhörn	r22 relaxed exclusion censoring	-6.76 [-23.07; 6.41]
Québec	r22 relaxed exclusion censoring	-1.74 [-4.43;0.41]
20th-century Sweden	r23 student cauchy priors	-4.60 [-7.26;-2.03]
Historical Sweden	r23 student cauchy priors	-7.27 [-13.28;-1.01]
Krummhörn	r23 student cauchy priors	-8.39 [-25.46;10.78]
Québec	r23 student cauchy priors	-3.04 [-6.17;0.21]
20th-century Sweden	r24 uniform priors	-4.71 [-7.13;-2.07]

Population	Model	Estimate
Historical Sweden	r24 uniform priors	-7.25 [-13.17;-1.03]
Krummhörn	r24 uniform priors	-8.90 [-26.92;11.29]
Québec	r24 uniform priors	-3.00 [-6.25;0.18]
Historical Sweden	r25 migration status	-10.44 [-16.68;-3.48]
Krummhörn	r25 migration status	-1.98 [-28.80;31.32]
Québec	r25 migration status	-2.98 [-6.22;0.38]
20th-century Sweden	r26 separate parental age contributions	-2.22 [-3.88;-0.44]

6.2 Model descriptions

6.2.1 *r1*: Relaxed exclusion criteria

For the three historical populations, we imposed quite stringent exclusion criteria to ensure sufficient data quality for our intended analysis. This was not necessary for the modern Swedish data, because there were no exclusion criteria to relax.

6.2.2 *r2*: Fewer covariates

Adding covariates increases the complexity of the model and makes it harder to interpret. We chose to adjust for many potential confounds because we are interested in causal isolation of the paternal age effect. Here we show what happens when only birth cohort and average paternal age in the family are adjusted for.

6.2.3 *r3*: Continuous birth order control

We chose to control for birth order/number of older siblings as a categorical variable, lumping all those who had more than 5 in the category 5+. Because a continuous covariate is also plausible, we tested this alternative model as well.

6.2.4 *r4*: Control number of dependent siblings

Birth order is usually used as a proxy variable for parental investment, the assumption being that older siblings require parental attention. However, there are are reasons to doubt this, as fully-grown siblings probably do not compete for the same resources. To compute a clearer proxy variable of competing siblings, we computed and adjusted for the number of siblings who were alive and younger than five at the time of birth of the anchor child.

6.2.5 *r5*: Birth order interacted with number of siblings

Plausibly, being first-born has a different effect, when one is an only child as opposed to having two siblings, etc. Here, we allow for such an interaction effect.

6.2.6 *r6*: No birth order control

Paternal age and birth order are highly collinear with each other and with maternal age. Therefore, the choice to include this predictor widens standard errors for each predictor and may be disputed. Here we show what happens when we simply omit the birth order control.

6.2.7 *r7*: Less control for parental loss

We adjusted for parental loss very stringently, including covariates for parental loss up to age 45. Here we show what happens, when we only control for parental loss in the first, and the first five years of life.

6.2.8 *r8*: Adjust for being first-/last-born adult son

Inheritance is linked to birth order and being male in several of the historical populations. Here, we adjust for the anchor being the first or last born adult son in a family. This implies that we control for our outcome to a certain extent, as "adult sons" cannot have died before adulthood, but a paternal age effect on mortality could still be detected for siblings other than the first- and last-born adults.

6.2.9 *r9*: Continuous birth year adjustment

In our main model, we control for birth cohort in 5-year-bins (lumping small bins). We chose to do so, because nonlinear and even sharply spiking effects of birth cohort are plausible (due to e.g. epidemics). This decision may be disputed, as it summarises 5-year-bins. Here, we instead allow for a thin-splate spline on the continuous birth year variable. This allows for smooth nonlinear (but not spiking) birth cohort effects.

6.2.10 *r10*: Group-level slope added

Paternal age effects may vary between different families. Although we did not explore between-family moderators of paternal age effects in our study, we tested whether modelling an additional group-level slope for paternal age differences within the family, would change the results by allowing for shrinkage and to examine the amount of inter-family differences to be explained for potential future moderator analysis.

6.2.11 *r11*: Separate group-level effects for each parent

Most anchors in our sample are full biological siblings and especially in the historical populations, divorce and remarriage was rare. Therefore, we chose to include only one group-level effect, for the parent couple (i.e. one group-level effect per father-mother-dyad). Including one intercept per parent is potentially a better way to adjust for genetic propensities inherited from either parent and allows estimating this propensity also from half-siblings, while half-sibling relationships were ignored in our main models. This comes at the cost of modelling complexity.

6.2.12 *r12*: Sex moderation

It need not be the case that paternal age has the same effect on male and female children. For example, male children inherit only the small Y chromosome from the father, but female children inherit the larger X chromosome, so that paternal age predicts X-chromosomal de novo mutations in females but not in males (Francioli et al., 2016). At the same time, the autism literature suggests that males are less robust to heritable and de novo autism risk variants and that these effects are not simply due to having only one X chromosome (Werling & Geschwind, 2015). Here we let a dummy variable for being male moderate the paternal age effect.

6.2.13 *r13*: Control paternal age at first birth

We already control for the average paternal age at which the children in a family were born. The mean is a more complete summary of the reproductive timing of the father than the age at first birth. However, far more literature has examined age at first birth and it has the advantage of never being censored (although we

of course try to rule out censoring by choosing appropriate subsets). Therefore, we added age at first birth as a covariate in this model.

6.2.14 *r14*: Compare lfe

Most of the previous literature has not used multilevel modelling, but linear group fixed effects (essentially dummy variables on the many thousands of families in the model). We believe our multilevel modelling approach has the advantage of allowing us to examine the effect of including predictors at the level of the family in the same model.

This allows us to
a) appropriately model a zero-inflated outcome such as number of children including those who died young (we're not aware of a linear group fixed effect approach that handles hurdle or zero-inflated models)
b) examine group-level slopes for paternal age and potentially to examine moderators at the level of the family (though we did not do this)
c) explicitly model confounders at the level of the family (e.g. number of siblings).

Nevertheless, the prevalence of this approach in the literature mandates that we show how our approach compares. We fit this model using the R package "lfe" and the function felm. All covariates that were not estimable in principle were removed (i.e. number of siblings, paternalage.mean).

Because we cannot extract an effect size comparable to the other models from these models, these results are viewable only online.

6.2.15 *r15*: Using a moderator by region, group-level effects by parish

In this model we attempted allow for regional variation in paternal age effects and attempted to better control residual variation. Our approach was two-fold: to moderate paternal age by region and to add a random effect for the church parish in which the individual was born. However, for the modern Swedish data, we had no geographic data and no regional information, so this model was not fit.

6.2.16 *r16*: Restrict to Skellefteå

Only in the DDB (historical Swedish data), parishes in some of the regions were still unlinked. This means that individuals could occur in more than one parish and not be linked. However, the region of Skellefteå was fully linked. Here, we test what happens when we restrict our dataset to Skellefteå.

6.2.17 *r17*: Simulating Down syndrome cases

1. We assume that 4 in 1000 births are children with Down syndrome (four times the actual rate).
2. We randomly excluded 33% of all children who had a mother older than 40 and had no children (many times the actual rate at that age).

6.2.18 *r18*: Reversing hurdle_poisson and poisson

To make models computationally feasible and because early mortality was negligible, we fit the very large modern Swedish dataset with a `poisson()` family distribution. All historical datasets had high early mortality, so we thought a `hurdle_poisson()` was more appropriate. Here, we show what happens when we reverse this. The `hurdle_poisson()` model can be fit to the modern Swedish data here, because we only use a subset.

6.2.19 *r19*: Normal distribution

Previous analysts sometimes decided to use the normal distribution to predict (potentially zero-inflated) count data. Here, we refit our models using a normal distribution for the outcome. We show that estimates for the paternal age effect can be estimated to have a substantially different magnitude, because of this, but did not change direction.

6.2.20 *r20*: No adjustment for maternal age

In this model, we test what happens when we do not adjust for maternal age, because it is highly collinear with paternal age.

6.2.21 *r21*: Continuous adjustment for maternal age

In this model, we adjust for maternal age using a continuous variable instead of three bins. This does not allow for nonlinear effects, but also does not aggregate the predictor. We cannot compare full siblings, test the effects of maternal and paternal age and adjust for average maternal and paternal age in the family (because the predictors are redundant), so that it is not perfectly possible to disentangle the contribution of maternal and paternal age and compare full siblings.

6.2.22 *r22*: Relaxed exclusion and censoring criteria

Like *r1*, but we use a 30-years-later cutoff year for our birth cohorts, relaxing our censoring requirements.

6.2.23 *r23*: Student's t and half-Cauchy priors

To demonstrate the robustness of our prior choice we use Student's t priors (fatter tails than normal priors) for our population-level effects and a half-Cauchy prior for our group-level effect for the family.

6.2.24 *r24*: Improper flat priors

To demonstrate the robustness of our prior choice we use improper flat priors. These priors should make the model's results comparable to a frequentist maximum likelihood approach.

6.2.25 *r25*: Adjust for migration status

In the three historical populations, records were kept in the parish. Although records were linked between parishes in all populations, except three out of four provinces in historical Sweden, migration might sometimes lead to censoring of records. Adjusting for migration may however constitute a partial adjustment for the outcome, as lower offspring fitness might make them more likely to migrate. Hence, we show the results of doing so as a robustness analysis. In all analyses, we adjusted for a "migrated"-dummy variable. Migration was differently defined depending on the population. In Québec, we had flags denoting immigrants and emigrants. Few immigrants were included in our analyses anyway, as we needed parental information for our analyses. Emigrants were people who left Québec. In historical Sweden, migration was logged as migration from the parish of birth. In the Krummhörn, we set migrated to true, when the parish of death/burial differed from the parish of birth/baptism.
No migration information was available in 20th-century Sweden, but records there weren't kept in parishes, so this should not pose a problem.

6.2.26 *r26*: Separate parental age contributions

In this model, we adjust for maternal age using a continuous variable. We also adjust for a dummy variable for teenage motherhood, to account for the nonlinearity of the maternal age effect. Moreover, we use separate random intercepts for mothers and fathers and adjust for the mother's mean age at birth and the father's mean age at birth. This model only converges in the 20th-century Sweden data, because there are sufficient numbers of divorces and remarriages and enough data to separate the parents' contributions.

7 Reproductive timing in Sweden

Reproductive timing data showed that average parental ages at birth decreased in 20th-century Sweden until ca. 1970 and increased thereafter. Average contemporary parental ages are still lower than in any of the three historical populations. Ages at first birth in the early periods and ages at last birth in the late periods are censored and hence biased towards the age at all births, which is itself unbiased.

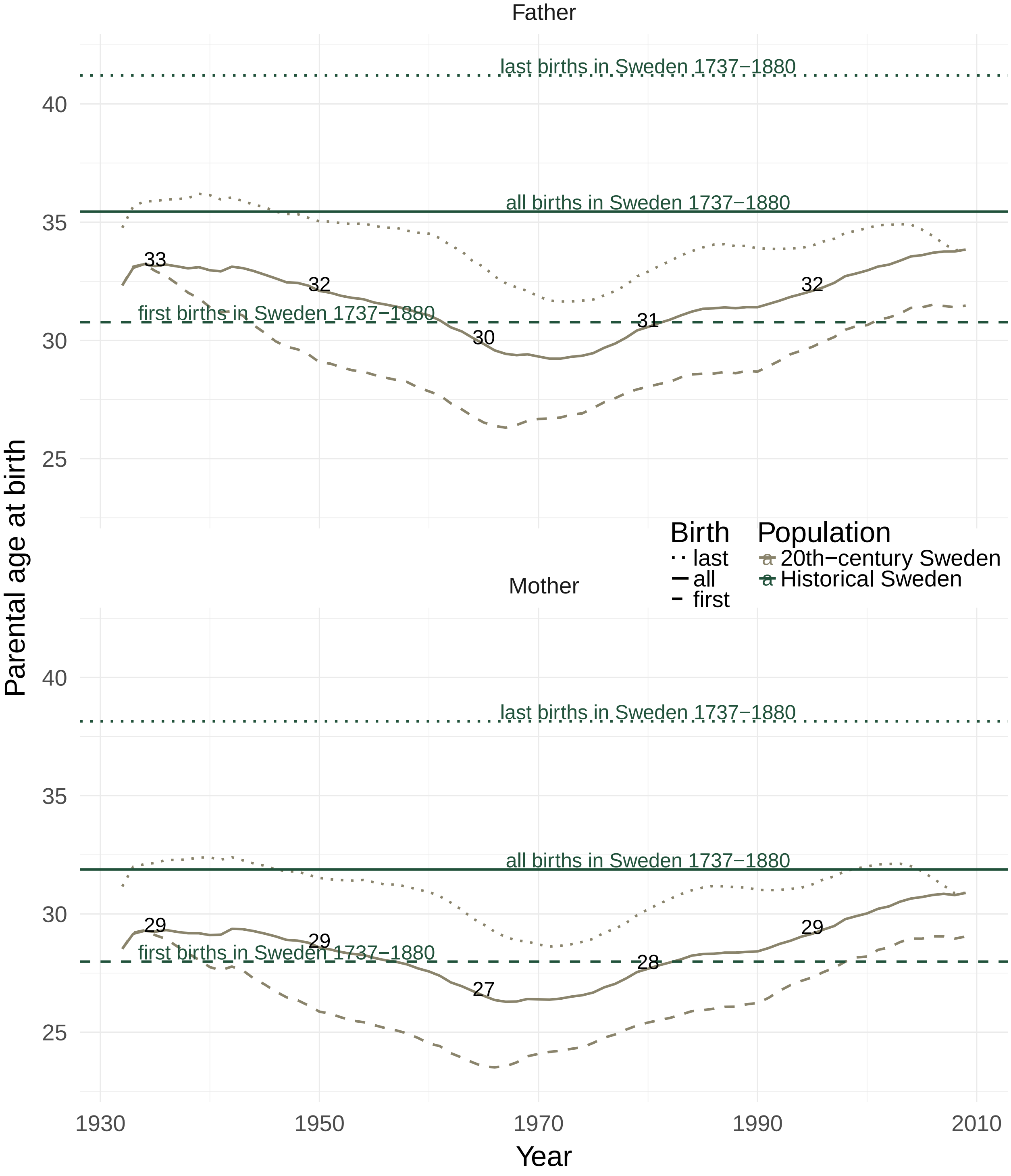

Supplement: Model documentation, reproductive timing [file rspb20171562supp1.pdf]
